# A Nickel/Organoboron-Catalyzed Coupling of Aryl Bromides with Sodium Sulfinates: The Synthesis of Sulfones under Visible Light

**DOI:** 10.3390/molecules29143418

**Published:** 2024-07-21

**Authors:** Siyi Ding, Weina Tian, Qiaohuan Lv, Zongcheng Miao, Liang Xu

**Affiliations:** 1 School of Electronic Information, Technological Institute of Materials & Energy Science (TIMES), Xi’an Key Laboratory of Advanced Photo-Electronics Materials and Energy Conversion Device, Xijing University, Xi’an 710123, China; dingsiyi2009@163.com (S.D.); l13720659398@163.com (Q.L.); 2 School of Chemistry and Chemical Engineering, State Key Laboratory Incubation Base for Green Processing of Chemical Engineering, Shihezi University, Shihezi 832003, China; 15389601065@163.com

**Keywords:** sulfone compounds, sodium sulfinates, dually catalytic system, organoboron photocatalyst

## Abstract

An efficient cross-coupling of aryl bromides with sodium sulfinates, using an organoboron photocatalyst with nickel, is described herein. Under the irradiation of white light, this dually catalytic system enables the synthesis of a series of sulfone compounds in moderate to good yields. A broad range of functional groups and heteroaromatic compounds is tolerated under these reaction conditions. The use of an organoboron photocatalyst highlights a sustainable alternative to iridium or ruthenium complexes. These findings contribute to the field of photochemistry and provide a greener approach to sulfone synthesis.

## 1. Introduction

Sulfone compounds have diverse applications in various fields such as medicine [1], bioactive molecules [2], and materials science [3,4]. The sulfone moiety is pivotal in the synthesis of numerous pharmaceuticals, including eletriptan for migraines [5] and bicalutamide for prostate cancer [6]. Furthermore, sulfone groups find application in important agrochemicals such as triketones [7], pyroxasulfone [8], or cafenstrole [9].

**Scheme 1:**
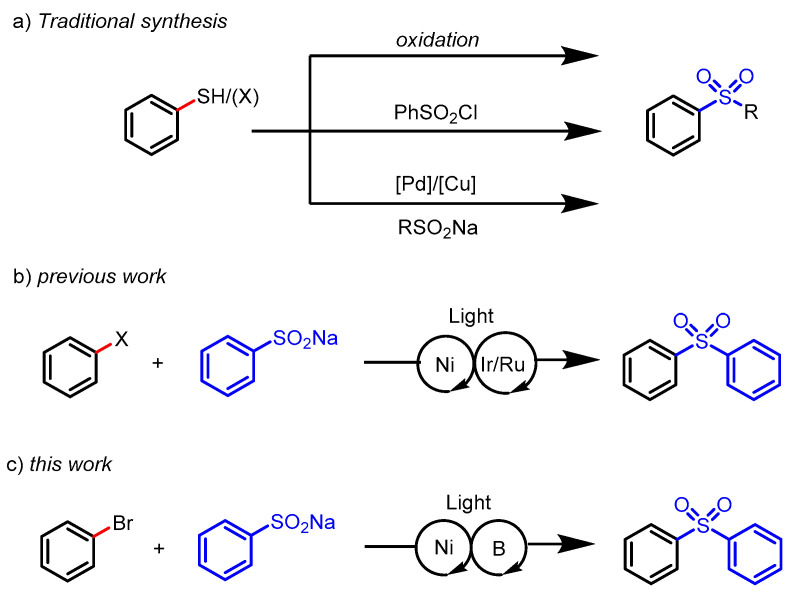
(**a**) Traditional synthesis method for the preparation of sulfone compounds. (**b**) Previous work: bimetallic catalysis of the coupling of aryl halides with sodium benzene sulfinate under blue light. (**c**) This work: nickel/organoboron catalysis of the coupling of aryl bromides with sodium sulfinate under white light.

Several methods for sulfone synthesis have been reported since the 19th century [10]. Traditionally, sulfone compounds have been synthesized through sulfide oxidation [11], the sulfonylation of aromatic hydrocarbons [12,13], and the arylation of sulfonates catalyzed by palladium or copper [14]. However, these reactions suffer from certain drawbacks, including high-temperature environments and harsh acidic treatment methods. These limitations can affect the functional group tolerance and substrate range of the reactions (Scheme 1a).

In recent years, nickel catalysts have gained significant attention in the field of transition-metal catalysis due to their exceptional catalytic performance [15,16,17,18]. The use of photo–nickel dual catalysis for C–C bond coupling has shown remarkable progress since 2014, as demonstrated by Molander [19] and MacMillan [20]. This photo–nickel dual catalysis has also been successfully employed in the synthesis of various C–X bonds [21,22,23], including aryl ethers, aryl esters, arylamines, triarylphosphine oxides, and sulfides. These advancements highlight the tremendous potential of utilizing dually catalytic strategies, not only to enhance existing reactions but also to discover novel synthetic approaches.

In 2018, Rueping, Manolikakes and Molander’s groups employed photocatalysts such as [Ir{(tBu)ppy}_2_{dtbbpy}]PF_6_ [24] or [Ru(bpy)_3_]^2+^ [25,26] to facilitate the nickel-promoted sulfonylation of aryl halides, respectively. More recently, Yang’s group reported the use of copper catalysts for the sulfonation of aryl halides with sulfinates under visible light. However, these reactions necessitate costly photocatalysts and/or ligands (Scheme 1b). In this context, our team has recently developed a boron-based complex called tetracoordinated aminoquinoline diarylboron (AQDAB). This boron-based complex was characterized by a noble-metal-free composition, easy modification of the chelating ligands, and quick convergent assembly of the coordinated structure, which made it more flexible than the traditional organic photocatalysts originated from linear synthesis. AQDAB has demonstrated successful application as a photocatalyst in a range of visible-light-induced transformations. These transformations include the photo–nickel dually catalytic construction of C–O bonds [27], C–S bonds, and C–P bonds [28]. It should be noted that Li’s group has recently disclosed a nickel/organic photocatalyst (2-chloro-thioxanthen-9-one) dual catalytic approach to access sulfones [29]. Herein, we present a method for synthesizing sulfones utilizing AQDAB as the photocatalyst in photo–nickel dual catalysis, employing aryl bromides and sodium sulfinate as the starting materials (Scheme 1c).

## 2. Results 

For C–S bond formation, the reaction was initiated using 4-bromobenzonitrile (**1a**) and sodium benzenesulfinate (**2a**) as the starting materials, aiming to synthesize the desired product **3a** through a photocatalytic strategy. Through a series of experiments, the optimal reaction conditions were identified. The highest yield of **3a** (85%) was achieved when the reaction was conducted under white light in DMSO under an argon atmosphere, using 1 mol% AQDAB as the photocatalyst, NiBr_2_∙3H_2_O as the metallic catalyst, dtbbpy (4,4′-di-tert-butyl-2,2′-bipyridine) as the ligand, and DIPEA as the base (Table 1, entry 1). Control experiments revealed the crucial roles played by light and the photocatalyst in the reaction. In the absence of light (entry 2), the reaction did not proceed. Since long-lasting irradiation could heat the reaction to ~40 °C, to test the possibility of a heating-induced reaction, the reaction was performed in a 40 °C oil bath for 24 or 48 h while shielding any light using aluminum foil: the product could not be observed under these conditions (entry 3). The presence of a photocatalyst was essential, as its absence led to a significant decrease in the yield, with only trace amounts of **3a** being detected (entry 4). Similarly, the nickel catalyst proved to be a critical factor (entry 5). The absence of dtbbpy resulted in a yield of 30%. This demonstrated that while the ligand was not necessary, its participation did improve the overall efficiency of this reaction (entry 6). These results collectively highlight the indispensability of the light, photocatalyst, nickel catalyst, and ligand in the reaction, demonstrating the dually catalytic property of this transformation. Subsequently, we investigated different nickel sources, attempting to replace NiBr_2_∙3H_2_O with alternative nickel compounds. However, such modifications led to a significant decrease in reaction yield (entries 7–12). Among the ligands, 20 mol% dtbbpy proved to be the optimal choice, as reducing its amount resulted in a noticeable decrease in yield (entry 13). Ligand-screening experiments revealed that *N*,*N*-chelating ligands based on the bipyridine structure exhibited superior catalytic effects compared to phenanthroline ligands (entries 14–16). Furthermore, we explored various solvents for the reaction. Substituting DMSO with alcohol-based solvents such as ethylene glycol and methanol inhibited the reaction, preventing the formation of the target product (entries 17, 18). The use of THF and MeCN as solvents did not yield the expected reaction (entries 19, 20). When highly polar DMF was employed as the solvent, the isolated yield was significantly decreased, with only 40% of the target product being obtained (entry 21). In the meanwhile, NMP exhibited limited effectiveness in promoting the reaction, resulting in a yield of only 48% (entry 22).

After obtaining the optimized reaction conditions, we investigated the scope of the reaction using different derivatives of aryl bromides and sodium aryl sulfinate.

**Scheme 2:**
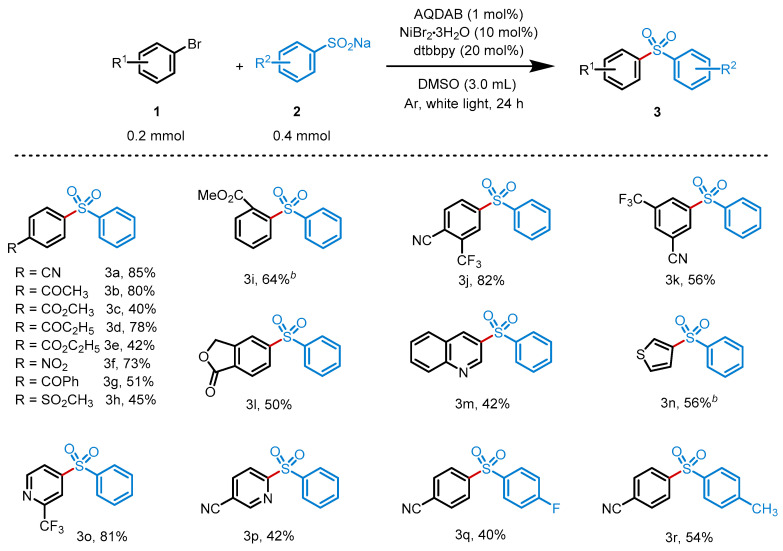
Substrate scope of sulfone compounds *^a^*. *^a^* Under standard reaction conditions: **1** (0.2 mmol, 1.0 equiv.), **2** (0.4 mmol, 2.0 equiv.), AQDAB (0.002 mmol, 1 mol%), NiBr_2_·3H_2_O (0.02 mmol, 10 mol%), dtbbpy (0.04 mmol, 20 mol%), and DMSO (3.0 mL); Kessil A160WE bulb (40 W, white light); Ar; 24 h. *^b^* Using aryl iodide as the substrate.

As shown in Scheme 2, when aryl bromides with *para*-electron-withdrawing groups on the benzene ring were coupled with sodium benzenesulfinate, moderate to good yields of various substituents were obtained. These substituents included cyano (**3a**, 85%), ketone (**3b**, 80%; **3d,** 78%; **3g**, 51%), ester (**3c**, 40%; **3e**, 42%), nitro (**3f**, 73%), and sulfonyl (**3h**, 45%). In the case of an ester group at the *ortho*-position of the halogenated benzene (**3i**), a yield of 64% was obtained, demonstrating that this method is not sensitive towards steric effects. The reaction also demonstrated good efficiency when two substituents were present, resulting in **3j** and **3k** yields of 82% and 56%, respectively. Moreover, the bromides on phthalide (**3l**, 50%), quinoline (**3m**, 42%), thiophene (**3n,** 56%), and pyridine (**3o**, 81%; **3p**, 42%) showed good compatibility towards the reaction conditions as well. This should be useful to synthesize heteroaryl sulfones. Next, we explored the suitability of different sodium aryl sulfinates. It was found that both electron-withdrawing and electron-donating groups could be coupled with **1a**, affording the corresponding sulfones in moderate yields (**3q**, **3r**).

However, it should be noted that electron-rich aryl halides and alkyl sodium sulfonates could not be tolerated under the reaction conditions. When they were treated, no corresponding C–S coupling products were obtained.

**Scheme 3:**
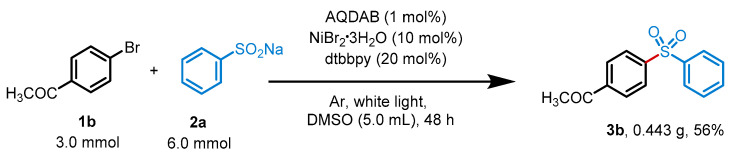
Gram-scale reaction.

To investigate the scalability of the reaction, a gram-scale reaction (Scheme 3) was performed and the reaction scale was increased by 15 times using 3.0 mmol of 1-(4-bromophenyl)ethan-1-one **1b** and 6.0 mmol of **2a**, while keeping the catalyst ratio unchanged. The gram-scale reaction was performed in a condensed solution and a prolonged reaction time was necessary to make the interplay between the photocatalyst, light, and reaction substrates efficient. After 48 h of irradiation and stirring, 1-(4-(phenylsulfonyl)phenyl)ethan-1-one **3b** was obtained with a yield of 56% (0.443 g).

## 3. Discussion 

**Scheme 4:**
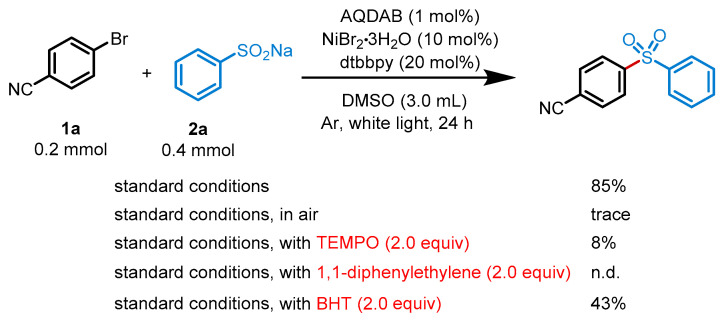
Control experiments.

To probe the reaction mechanism, a series of control experiments was performed (Scheme 4). The reaction exhibited high sensitivity to oxygen and was inhibited under an air atmosphere. It was imagined that the presence of O_2_ might interrupt the Ni-catalyzed cycle by oxidizing the low-valent nickel intermediates to unreactive resting species. Additionally, we examined the impacts of several radical scavengers on the reaction. When TEMPO was present, the isolated yield of **3a** was 8%. Similarly, in the presence of 1,1-diphenylethylene, the desired product could not be obtained. When BHT was employed as the radical scavenger, the reaction yield decreased significantly. These experiments indicated the potential involvement of radical processes in the reaction. Then, on/off irradiation experiments were performed based on the model reaction in Table 1.

**Figure 1:**
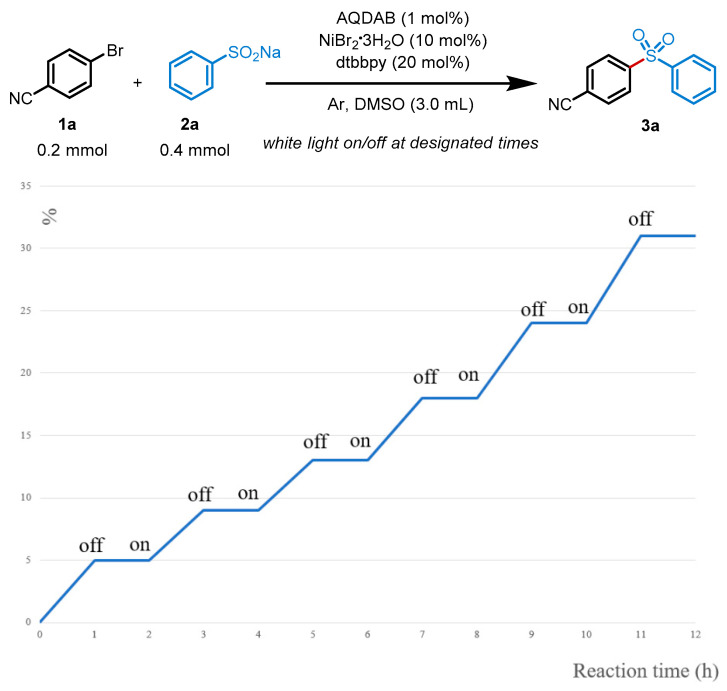
The effect of on/off irradiation on the reaction yield.

As shown in Figure 1, a series of reactions with the same substrates and catalysts was performed parallelly. The effect of on/off irradiation was explored by quenching one reaction and isolating the corresponding product at designated times. When the irradiation was turned off, the formation of the desired product stopped correspondingly. Further stirring in the absence of irradiation did not afford any more product. The reaction would proceed to accumulate product only if the light irradiation was turned on again. For example, after 1 h of stirring under irradiation, the light was turned off and **3a** was isolated at a 5% yield from one reaction. A further 1 h of stirring without light irradiation (2 h’ stirring in total) led to a 5% yield of the reaction, still. Then, the light was turned on to repeat the above-described process.

**Scheme 5:**
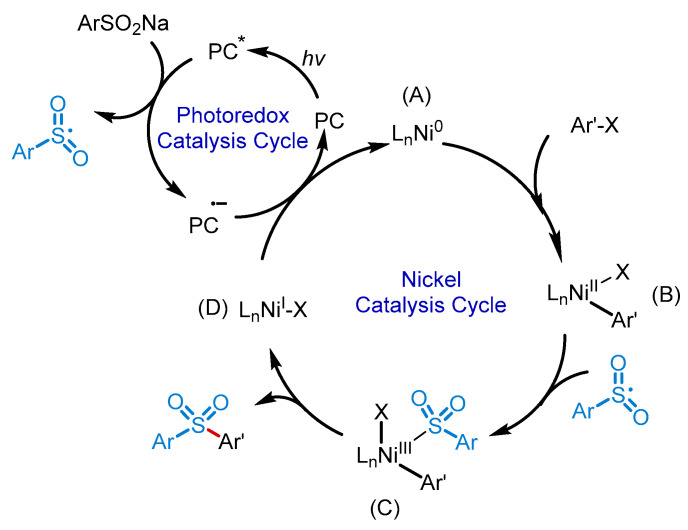
A proposed reaction mechanism for the sulfonylation of aryl bromides.

Based on our experimental findings and relevant works in the literature [24,25,26,27,28,29,30], we propose a plausible mechanism for this reaction, as shown in Scheme 5. Upon illumination, the photocatalyst (**PC**) is excited to form PC*, which undergoes single-electron transfer with the sodium aryl sulfinates, leading to the generation of **ArSO_2_^•^** and **PC^•−^**. Subsequently, oxidative addition occurs between the Ni (0) compound **A** and aryl bromides, resulting in the formation of the Ni (II) intermediate **B**. Further single-electron oxidative addition between **B** and **ArSO_2_^•^** yields complex **C** (**Ni^III^**). Finally, complex **C** undergoes a reduction elimination process, leading to the formation of the desired product and **D** (**Ni^I^**). Compound **D**, along with **PC^•−^**, undergoes single-electron transfer to regenerate **Ni^0^** and **PC**, thus completing both the photocatalytic and Ni-catalyzed reaction cycles.

## 4. Materials, Methods, Reaction Procedure, and Analytical Data

### 4.1. Methods and Materials

**General Information:** Unless otherwise noted, all reactions were carried out under an Ar atmosphere. Analytical thin-layer chromatography (TLC) was performed on glass plates coated with 0.25 mm 230–400-mesh silica gel containing a fluorescent indicator. Visualization was accomplished through exposure to a UV lamp. All of the products mentioned in this article are compatible with standard silica gel chromatography. Column chromatography was performed on the silica gel (200–300 mesh) using standard methods. 

**Structural Analysis:** NMR spectra were measured on a Bruker Ascend 400 spectrometer and chemical shifts (*δ*) are reported in parts per million (ppm). ^1^H NMR spectra were recorded at 400 MHz in NMR solvents and referenced internally to the corresponding solvent resonance, and ^13^C NMR spectra were recorded at 101 MHz and referenced to the corresponding solvent resonance. Coupling constants are reported in Hz with multiplicities denoted as s (singlet), d (doublet), t (triplet), q (quartet), m (multiplet), and br (broad). Infrared spectra were collected on a Thermo Fisher Nicolet 6700 FT-IR spectrometer using the ATR (Attenuated Total Reflectance) method. Absorption maxima (ν max) are reported in wavenumbers (cm^−1^). High-resolution mass spectra (HRMS) were acquired on a Thermo Scientific LTQ Orbitrap XL with an ESI source. 

**Materials:** Commercial reagents and solvents were purchased from Adamas, J&K, Energy, Sigma-Aldrich, Alfa Aesar, Acros Organics, and TCI and were used as received unless otherwise stated.

### 4.2. The Preparation of Sulfone Compounds

#### 4.2.1. General Procedure

A flame-dried 25 mL quartz column reaction tube was placed with a magnetic stir bar. Then, 4-bromobenzonitrile (36.4 mg, 0.2 mmol, 1.0 equiv.), sodium benzene sulfinate (65.6 mg, 0.4 mmol, 2.0 equiv.), **AQDAB** (0.8 mg, 0.002 mmol, 1 mol%), NiBr_2_·3H_2_O (5.4 mg, 0.02 mmol, 10 mol%), dtbbpy (10.7 mg, 0.04 mmol, 20 mol%), and DMSO (3.0 mL) were added under Ar. The reaction tube was placed on a photocatalytic parallel reactor with a Kessil LED light source on the side. A fan was used during the irradiation to cool down the reaction tube (the temperature could reach and stay at ~40 °C). After stirring for 24 h, the crude product was concentrated and purified with column chromatography (silica gel) to obtain the target product, using PE/EtOAc as the eluent. The detail experimental information can be found in Supplementary Materials.

#### 4.2.2. Analytical Data of Products **3a**–**3r**

*(***3a***) 4-(phenylsulfonyl)benzonitrile (CAS: 28525-13-5)* [29]

Following the General Procedure with 4-bromobenzonitrile (36.4 mg, 0.2 mmol, 1.0 equiv.) and sodium benzenesulfinate (65.6 mg, 0.4 mmol, 2.0 equiv.), **3a** was obtained as a white solid (41.3 mg, 85%). This product was purified with silica gel flash chromatography (PE:EA = 5:1).

^1^H NMR (400 MHz, CDCl_3_) δ 8.05 (d, *J* = 8.4 Hz, 2H), 7.94 (d, *J* = 7.6 Hz, 2H), 7.79 (d, *J* = 8.0 Hz, 2H), 7.61 (d, *J* = 7.2 Hz, 1H), 7.54 (t, *J* = 7.6 Hz, 2H).

^13^C NMR (101 MHz, CDCl_3_) δ 145.9, 140.2, 134.1, 133.1, 129.7, 128.3, 128.0, 117.2, 116.9.

*(***3b***) 1-(4-(phenylsulfonyl)phenyl)ethan-1-one (CAS: 65085-83-8)* [29]

Following the General Procedure with 1-(4-bromophenyl)ethan-1-one (39.8 mg, 0.2 mmol, 1.0 equiv.) and sodium benzenesulfinate (65.6 mg, 0.4 mmol, 2.0 equiv.), **3b** was obtained as a white solid (41.6 mg, 80%). This product was purified using silica gel flash chromatography (PE:EA = 5:1).

^1^H NMR (400 MHz, CDCl_3_) δ 8.05 (s, 4H), 7.96 (d, *J* = 8.0 Hz, 2H), 7.59 (d, *J* = 6.0 Hz, 1H), 7.54 (d, *J* = 6.8 Hz, 2H), 2.62 (s, 3H).

^13^C NMR (101 MHz, CDCl_3_) δ 196.7, 145.4, 140.8, 140.4, 133.7, 129.5, 129.1, 128.0, 127.9, 26.9.

*(***3c***) Methyl 4-(phenylsulfonyl)benzoate (CAS: 38337-00-7)* [29]

Following the General Procedure with methyl 4-bromobenzoate (42.8 mg, 0.2 mmol, 1.0 equiv.) and sodium benzenesulfinate (65.6 mg, 0.4 mmol, 2.0 equiv.), **3c** was obtained as a white solid (22.1 mg, 40%). This product was purified using silica gel flash chromatography (PE:EA = 5:1).

^1^H NMR (400 MHz, CDCl_3_) δ 8.14 (d, *J* = 8.4 Hz, 2H), 8.00 (d, *J* = 8.5 Hz, 2H), 7.95 (d, *J* = 7.3 Hz, 2H), 7.58 (d, *J* = 7.2 Hz, 1H), 7.52 (t, *J* = 6.8 Hz, 2H), 3.92 (s, 3H).

^13^C NMR (101 MHz, CDCl_3_) δ 165.5, 145.5, 140.8, 134.3, 133.6, 130.5, 129.5, 127.8, 127.7, 52.7.

*(* **3d** *) 1-(4-(phenylsulfonyl)phenyl)propan-1-one (CAS: 69567-00-6)*

Following the General Procedure with methyl 1-(4-bromophenyl)propan-1-one (45.6 mg, 0.2 mmol, 1.0 equiv.) and sodium benzenesulfinate (65.6 mg, 0.4 mmol, 2.0 equiv.), **3d** was obtained as a white solid (42.7 mg, 78%). This product was purified with silica gel flash chromatography (PE:EA = 5:1).

^1^H NMR (400 MHz, CDCl_3_) δ 8.02 (d, *J* = 5.2 Hz, 4H), 7.94 (d, *J* = 8.4 Hz, 2H), 7.56 (d, *J* = 7.2 Hz, 1H), 7.50 (t, *J* = 7.6 Hz, 2H), 2.97 (d, *J* = 7.2 Hz, 2H), 1.18 (s, 3H).

^13^C NMR (101 MHz, CDCl_3_) δ 199.5, 145.2, 140.8, 140.3, 133.7, 129.5, 128.8, 128.0, 127.8, 32.3, 8.0.

HRMS (ESI) *m*/*z* calcd. for C_15_H_15_O_3_S^+^ M+H^+^: 275.3415; found: 275.3415.

IR: 3070, 2978, 2935, 1697, 1582, 1448, 1155, 730, 600.

Melting point (°C): 57.9–59.1

*(***3e***) Ethyl 4-(phenylsulfonyl)benzoate (CAS: 101094-06-8)* [29]

Following the General Procedure with ethyl 4-bromobenzoate (45.8 mg, 0.2 mmol, 1.0 equiv.) and sodium benzenesulfinate (65.6 mg, 0.4 mmol, 2.0 equiv.), **3e** was obtained as a white solid (24.4 mg, 42%). This product was purified with silica gel flash chromatography (PE:EA = 5:1).

^1^H NMR (400 MHz, CDCl_3_) δ 8.15 (d, *J* = 8.0 Hz, 2H), 8.00 (d, *J* = 8.0 Hz, 2H), 7.95 (d, *J* = 8.0 Hz, 2H), 7.58 (d, *J* = 7.2 Hz, 1H), 7.52 (d, *J* = 7.6 Hz, 2H), 4.38 (d, *J* = 7.2 Hz, 2H), 1.38 (s, 3H).

^13^C NMR (101 MHz, CDCl_3_) δ 165.0, 145.4, 140.9, 134.7, 133.6, 130.4, 129.5, 129.3, 127.8, 127.7, 61.7, 14.2.

*(***3f***) 1-nitro-4-(phenylsulfonyl)benzene (CAS: 1146-39-0)* [29]

Following the General Procedure with methyl 1-bromo-4-nitrobenzene (40.0 mg, 0.2 mmol, 1.0 equiv.) and sodium benzenesulfinate (65.6 mg, 0.4 mmol, 2.0 equiv.), **3f** was obtained as a white solid (38.3 mg, 73%). This product was purified using silica gel flash chromatography (PE:EA = 5:1).

^1^H NMR (400 MHz, CDCl_3_) δ 8.33 (d, *J* = 8.8 Hz, 2H), 8.13 (d, *J* = 6.7 Hz, 2H), 7.97 (d, *J* = 7.9 Hz, 2H), 7.62 (d, *J* = 6.4 Hz, 1H), 7.56 (d, *J* = 6.9 Hz, 2H).

^13^C NMR (101 MHz, CDCl_3_) δ 150.4, 147.4, 140.0, 134.2, 129.7, 129.0, 128.1, 124.6.

*(***3g***) Phenyl(4-(phenylsulfonyl)phenyl)methanone (CAS: 54687-39-7)* [29]

Following the General Procedure with (4-bromophenyl)(phenyl)methanone (52.0 mg, 0.2 mmol, 1.0 equiv.) and sodium benzenesulfinate (65.6 mg, 0.4 mmol, 2.0 equiv.), **3g** was obtained as a white solid (32.8 mg, 51%). This product was purified with silica gel flash chromatography (PE:EA = 5:1).

^1^H NMR (400 MHz, CDCl_3_) δ 8.06 (d, *J* = 6.4 Hz, 2H), 7.99 (d, *J* = 7.6 Hz, 2H), 7.88 (d, *J* = 6.8 Hz, 2H), 7.77 (d, *J* = 7.6 Hz, 2H), 7.66–7.59 (m, 2H), 7.56 (d, *J* = 6.8 Hz, 2H), 7.49 (s, 2H).

^13^C NMR (101 MHz, CDCl_3_) δ 195.2, 144.7, 141.7, 140.9, 136.4, 133.7, 133.3, 130.5, 130.1, 129.5, 128.6, 127.9, 127.7.

*(***3h***) 1-(methylsulfonyl)-4-(phenylsulfonyl)benzene (CAS: 3112-84-3)* [26]

Following the General Procedure with 1-bromo-4-(methylsulfonyl)benzene (46.4 mg, 0.2 mmol, 1.0 equiv.) and sodium benzenesulfinate (65.6 mg, 0.4 mmol, 2.0 equiv.), **3h** was obtained as a white solid (26.6 mg, 45%). This product was purified with silica gel flash chromatography (PE:EA = 5:1).

^1^H NMR (400 MHz, CDCl_3_) δ 8.14 (d, *J* = 8.4 Hz, 2H), 8.08 (d, *J* = 8.4 Hz, 2H), 7.97 (d, *J* = 7.2 Hz, 2H), 7.63 (d, *J* = 6.8 Hz, 1H), 7.57 (d, *J* = 8.0 Hz, 2H), 3.07 (s, 3H).

^13^C NMR (101 MHz, CDCl_3_) δ 146.8, 144.8, 140.2, 134.1, 129.7, 128.7, 128.5, 128.0, 44.3.

*(***3i***) Methyl 2-(phenylsulfonyl)benzoate (CAS: 67373-14-2)* [29]

Following the General Procedure with methyl 2-iodobenzoate (52.4 mg, 0.2 mmol, 1.0 equiv.) and sodium benzenesulfinate (65.6 mg, 0.4 mmol, 2.0 equiv.), **3i** was obtained as a white solid (35.3 mg, 64%). This product was purified with silica gel flash chromatography (PE:EA = 5:1).

^1^H NMR (400 MHz, CDCl_3_) δ 8.15 (m, 1H), 7.97 (d, *J* = 7.2 Hz, 2H), 7.62 (m, 2H), 7.57 (m, 2H), 7.51 (t, *J* = 7.4 Hz, 2H), 3.92 (s, 3H).

^13^C NMR (101 MHz, CDCl_3_) δ 167.7, 141.5, 139.0, 133.3, 130.9, 130.2, 129.2, 129.0, 127.8, 53.0.

*(***3j***) 4-(phenylsulfonyl)-2-(trifluoromethyl)benzonitrile (CAS: 2383030-86-0)* [29]

Following the General Procedure with 4-bromo-2-(trifluoromethyl)benzonitrile (49.8 mg, 0.2 mmol, 1.0 equiv.) and sodium benzenesulfinate (65.6 mg, 0.4 mmol, 2.0 equiv.), **3j** was obtained as a white solid (51.0 mg, 82%). This product was purified with silica gel flash chromatography (PE:EA = 5:1).

^1^H NMR (400 MHz, CDCl_3_) δ 8.34 (s, 1H), 8.25 (d, *J* = 8.0 Hz, 1H), 8.04–7.95 (m, 3H), 7.68 (t, *J* = 7.4 Hz, 1H), 7.59 (t, *J* = 7.6 Hz, 2H).

^13^C NMR (101 MHz, CDCl_3_) δ 146.6, 139.3, 135.9, 134.6, 134.2 (d, *J* = 34.0 Hz), 131.3, 130.0, 128.2, 125.8 (q, *J* = 4.7 Hz), 121.5(d, *J* = 275.8 Hz), 114.5, 114.0.

^19^F NMR (376 MHz, CDCl_3_) δ −62.10 (s).

*(***3k***) 3-(phenylsulfonyl)-5-(trifluoromethyl)benzonitrile* [29]

Following the General Procedure with 3-bromo-5-(trifluoromethyl)benzonitrile (40.0 mg, 0.2 mmol, 1.0 equiv.) and sodium benzenesulfinate (65.6 mg, 0.4 mmol, 2.0 equiv.), **3k** was obtained as a white solid (34.8 mg, 56%). This product was purified using silica gel flash chromatography (PE:EA = 5:1).

^1^H NMR (400 MHz, CDCl_3_) δ 8.40 (d, *J* = 10.4 Hz, 2H), 8.08 (s, 1H), 7.99 (d, *J* = 8.4 Hz, 2H), 7.68 (m, 1H), 7.60 (m, 2H).

^13^C NMR (101 MHz, CDCl_3_) δ 145.1, 139.3, 134.6, 134.3, 133.5 (d, *J* = 35.0 Hz), 133.0 (d, *J* = 3.6 Hz), 130.2, 128.3 (q, *J* = 3.6), 128.2, 127.7, 122.0 (d, *J* = 274.8 Hz), 115.7, 115.2.

^19^F NMR (376 MHz, CDCl_3_) δ −63.06 (s).

*(***3l***) 5-(phenylsulfonyl)isobenzofuran-1(3H)-one (CAS: 2232133-50-3)* [26]

Following the General Procedure with 5-bromoisobenzofuran-1(3H)-one (42.4 mg, 0.2 mmol, 1.0 equiv.) and sodium benzenesulfinate (65.6 mg, 0.4 mmol, 2.0 equiv.), **3l** was obtained as a white solid (27.4 mg, 50%). This product was purified using silica gel flash chromatography (PE:EA = 5:1).

^1^H NMR (400 MHz, CDCl_3_) δ 8.14 (s, 1H), 8.09 (d, *J* = 8.0 Hz, 1H), 8.03 (d, *J* = 8.0 Hz, 1H), 7.99–7.96 (m, 2H), 7.64 (m, 1H), 7.55 (t, *J* = 7.8 Hz, 2H), 5.39 (s, 2H).

^13^C NMR (101 MHz, CDCl_3_) δ 169.1, 147.3, 147.2, 140.3, 134.0, 129.8, 129.7, 128.6, 128.0, 126.9, 122.0, 69.6.

*(***3m***) 3-(phenylsulfonyl)quinoline (CAS: 117620-35-6)* [29]

Following the General Procedure with methyl 1-bromo-4-nitrobenzene (40.0 mg, 0.2 mmol, 1.0 equiv.) and sodium benzenesulfinate (65.6 mg, 0.4 mmol, 2.0 equiv.), **3m** was obtained as a white solid (22.6 mg, 42%). This product was purified with silica gel flash chromatography (PE:EA = 5:1).

^1^H NMR (400 MHz, CDCl_3_) δ 9.28 (d, *J* = 2.0 Hz, 1H), 8.83 (d, *J* = 2.0 Hz, 1H), 8.16 (d, *J* = 8.4 Hz, 1H), 8.07–8.01 (m, 2H), 7.97 (d, *J* = 8.4 Hz, 1H), 7.87 (m, 1H), 7.68 (m, 1H), 7.63–7.57 (m, 1H), 7.54 (m, 2H).

^13^C NMR (101 MHz, CDCl_3_) δ 149.4, 147.1, 141.0, 137.0, 134.7, 133.8, 132.8, 129.6, 129.2, 128.4, 127.8, 126.4.

*(***3n***) 3-(phenylsulfonyl)thiophene (CAS: 89770-30-9)* [31]

Following the General Procedure with 3-iodothiophene (40.0 mg, 0.2 mmol, 1.0 equiv.) and sodium benzenesulfinate (44.6 mg, 0.4 mmol, 2.0 equiv.), **3n** was obtained as a white solid (25.0 mg, 56%). This product was purified using silica gel flash chromatography (PE:EA = 5:1).

^1^H NMR (400 MHz, CDCl_3_) δ 8.13–8.08 (m, 1H), 7.97 (d, *J* = 7.2 Hz, 2H), 7.59 (t, *J* = 6.8 Hz, 1H), 7.52 (t, *J* = 7.4 Hz, 2H), 7.41–7.36 (m, 1H), 7.36–7.32 (m, 1H).

^13^C NMR (101 MHz, CDCl_3_) δ 142.0, 141.6, 133.3, 131.6, 129.3, 128.4, 127.5, 125.9.

*(***3o***) 4-(phenylsulfonyl)-2-(trifluoromethyl)pyridine (CAS: 2412989-04-7)* [32]

Following the General Procedure with 4-bromo-2-(trifluoromethyl)pyridine (44.9 mg, 0.2 mmol, 1.0 equiv.) and sodium benzenesulfinate (65.6 mg, 0.4 mmol, 2.0 equiv.), **3o** was obtained as a white solid (46.5 mg, 81%). This product was purified with silica gel flash chromatography (PE:EA = 5:1).

^1^H NMR (400 MHz, CDCl_3_) δ 8.93 (d, *J* = 4.8 Hz, 1H), 8.13 (s, 1H), 7.99 (d, *J* = 8.0 Hz, 3H), 7.74–7.63 (m, 1H), 7.63–7.53 (m, 2H).

^13^C NMR (101 MHz, CDCl_3_) δ 152.2, 151.7, 150.0 (d, *J =* 36.1 Hz), 138.8, 134.7, 130.0, 128.3, 123.7, 120.7 (d, *J* = 275.8 Hz), 117.79 (d, *J* = 2.7 Hz).

^19^F NMR (376 MHz, CDCl_3_) δ −68.06 (s).

*(* **3p** *) 6-(phenylsulfonyl)nicotinonitrile (205514-29-0)*

Following the General Procedure with 2-Bromo-4-cyanopyridine (36.6 mg, 0.2 mmol, 1.0 equiv.) and sodium benzenesulfinate (65.6 mg, 0.4 mmol, 2.0 equiv.), **3p** was obtained as a white solid (20.5 mg, 42%). This product was purified with silica gel flash chromatography (PE:EA = 5:1).

^1^H NMR (400 MHz, CDCl_3_) δ 8.89 (d, *J* = 1.1 Hz, 1H), 8.34 (d, *J* = 8.0 Hz, 1H), 8.23 (dd, *J* = 8.4, 2.0 Hz, 1H), 8.06 (d, *J* = 7.2 Hz, 2H), 7.68 (t, *J* = 6.8 Hz, 1H), 7.58 (t, *J* = 7.6 Hz, 2H).

^13^C NMR (101 MHz, CDCl_3_) δ 161.7, 152.7, 141.9, 137.5, 134.6, 129.4, 129.4, 121.9, 115.1, 113.0.

HRMS (ESI) *m*/*z* calcd. for C_12_H_9_N_2_O_2_S^+^ M+H^+^: 245.0371; found: 245.0371.

IR: 3065, 2243, 1452, 325, 1161, 628.

Melting point (°C): 119.1–120.1

*(***3q***) 4-((4-fluorophenyl)sulfonyl)benzonitrile (CAS: 1268049-80-4)* [29]

Following the General Procedure with 4-Bromobenzonitrile (36.4 mg, 0.2 mmol, 1.0 equiv.) and 4-flurobenzenesulfinic acid sodium salt (91.0 mg, 0.4 mmol, 2.0 equiv.), **3q** was obtained as a white solid (20.8 mg, 40%). This product was purified with silica gel flash chromatography (PE:EA = 5:1).

^1^H NMR (400 MHz, CDCl_3_) δ 8.05 (d, *J* = 8.4 Hz, 2H), 8.02–7.95 (m, 2H), 7.81 (d, *J* = 8.0 Hz, 2H), 7.24 (dd, *J* = 16.0, 8.0 Hz, 2H).

^13^C NMR (101 MHz, CDCl_3_) δ 166.0 (d, *J* = 258.7 Hz), 145.7, 136.2, 133.2, 130.9 (d, *J* = 9.8 Hz), 128.2, 117.2, 117.1 (d, *J* = 3.7 Hz), 117.0.

^19^F NMR (376 MHz, CDCl_3_) δ −102.34 (s).

*(***3r***) 4-tosylbenzonitrile (CAS: 38111-56-7)* [29]

Following the General Procedure with 4-Bromobenzonitrile (36.4 mg, 0.2 mmol, 1.0 equiv.) and 4-methyl-benzenesulfinic acid sodium salt (71.2 mg, 0.4 mmol, 2.0 equiv.), **3r** was obtained as a white solid (27.8 mg, 54%). This product was purified using silica gel flash chromatography (PE:EA = 5:1).

^1^H NMR (400 MHz, CDCl_3_) δ 8.04 (d, *J* = 6.8 Hz, 2H), 7.83 (d, *J* = 6.8 Hz, 2H), 7.81–7.76 (m, 2H), 7.34 (d, *J* = 7.6 Hz, 2H), 2.42 (s, 3H).

^13^C NMR (101 MHz, CDCl_3_) δ 146.3, 145.3, 137.1, 133.1, 130.3, 128.1, 128.0, 117.2, 116.7, 21.7.

## 5. Conclusions

In conclusion, by employing the organoboron compound AQDAB as a photocatalyst in conjunction with nickel, we have realized a cross-coupling reaction between aryl bromides and sodium aryl sulfinates under relatively mild conditions and afforded various sulfone compounds in moderate to good yields. This procedure tolerates a series of functional groups, including cyanide, nitro, carbonyl, and ester groups. We anticipate that the protocol described herein can serve as an important supplement to the existing strategies for preparing sulfone compounds, and thus find wide application in organic synthesis and related fields.

## Figures and Tables

**Table 1:**
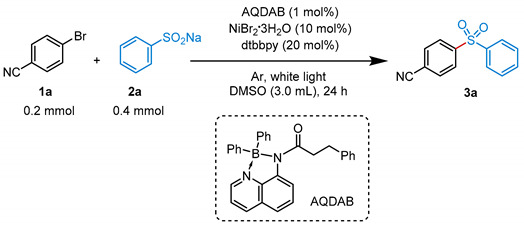
Optimization of reaction conditions *^a^*.

Entry	Variation from Standard Conditions		Yield *^b^*
**1**	Control experiments	None	85%
**2**		Without light or under sunlight	n.d.
**3**		Heating the reaction at 40 °C while shielding any light for 24 h or 48 h	n.d.
**4**		Without AQDAB	trace
**5**		Without NiBr_2_·3H_2_O	n.d.
**6**		Without dtbbpy	30%
**7**	The screening of Ni catalysts	NiBr_2_·dme	35%
**8**		NiBr_2_	71%
**9**		NiCl_2_	58%
**10**		NiCl_2_·dme	40%
**11**		Ni(acac)_2_	trace
**12**		NiCl_2_·6H_2_O	48%
**13**	The screening of ligands	dtbbpy (10 mol%)	55%
**14**		dpy (20 mol%)	70%
**15**		dmedpy (20 mol%)	55%
**16**		1,10-phen (20 mol%)	26%
**17**	The screening of solvents	(CH_2_OH)_2_	n.d.
**18**		CH_3_OH	n.d.
**19**		THF	n.d.
**20**		CH_3_CN	n.d.
**21**		DMF	40%
**22**		NMP	48%

*^a^* Standard reaction conditions: (**1a**) (0.2 mmol, 1.0 equiv.), (**2a**) (0.4 mmol, 2.0 equiv.), AQDAB (0.002 mmol, 1 mol%), NiBr_2_∙3H_2_O (0.02 mmol, 10 mol%), dtbbpy (0.04 mmol, 20 mol%), and DMSO (3.0 mL); Kessil A160WE bulb (40 W, white light); Ar; 24 h. A fan was used during the irradiation to cool down the reaction tube (the temperature could reach and stay at ~40 °C). *^b^* Isolated yield. n.d.: not detected, dtbbpy: 4,4-di-tert-butyl bipyridine, dpy: 2,2′-bipyridine; dmedpy: 4,4′-dimethyl-2,2′-bipyridine; 1,10-phen: 1,10-phenanthroline.

## Data Availability

The original data presented in the study are openly available in Supplementary Materials.

## Supplementary Materials

The following supporting information can be downloaded at: https://www.mdpi.com/article/10.3390/molecules29143418/s1, Experimental procedures and spectral data are available free of charge via the Internet.
