# Supplementary material for: A Nickel/Organoboron-Catalyzed Coupling of Aryl Bromides with Sodium Sulfinates: The Synthesis of Sulfones under Visible Light
Source: Molecules. 2024 Jul 21;29(14):3418. doi: 10.3390/molecules29143418 (PMC11280069; doi:10.3390/molecules29143418)
Supplement: Supplementary file 1 [file molecules-29-03418-s001.zip › molecules-3071106-supplementary.pdf]

A Nickel/Organoboron Catalyzed Coupling of Aryl Bromides with Sodium Sulfinates: Synthesis of Sulfones under Visible Light

Siyi Ding [1,#], Weina Tian [2,#], Qiaohuan Lv [1], Zongcheng Miao [1,*] Liang Xu[2,*]

1 Technological Institute of Materials & Energy Science (TIMES), Xi'an Key Laboratory of Advanced Photo-Electronics Materials and Energy Conversion Device, School of Electronic Information, Xijing University, Xi 'an 710123, China.

2 State Key Laboratory Incubation Base for Green Processing of Chemical Engineering, School of Chemistry and Chemical Engineering, Shihezi University, Shihezi, Xinjiang 832003.

Content

1. General considerations 1

2. Characterization data 2

3. References.. 8

4. Copies of NMR spectra 9

1. General considerations

General. Unless otherwise noted, all reactions were carried out under an air atmosphere. Analytical thin-layer chromatography (TLC) was performed on glass plates coated with 0.25 mm 230–400 mesh silica gel containing a fluorescent indicator. Visualization was accomplished by exposure to a UV lamp. All the products in this article are compatible with standard silica gel chromatography. Column chromatography was performed on silica gel (200–300 mesh) using standard methods.

Structural analysis. NMR spectra were measured on a Bruker Ascend 400 spectrometer and chemical shifts (*δ*) are reported in parts per million (ppm). ^{1}H NMR spectra were recorded at 400 MHz in NMR solvents and referenced internally to corresponding solvent resonance, and ^{13}C NMR spectra were recorded at 101 MHz and referenced to corresponding solvent resonance. Coupling constants are reported in Hz with multiplicities denoted as s (singlet), d (doublet), t (triplet), q (quartet), m (multiplet) and br (broad). Infrared spectra were collected on a Thermo Fisher Nicolet 6700 FT-IR spectrometer using ATR (Attenuated Total Reflectance) method. Absorption maxima (ν max) are reported in wavenumbers (cm^{-1}). High resolution mass spectra (HRMS) were acquired on Thermo Scientific LTQ Orbitrap XL with an ESI source.

Materials. Commercial reagents and solvent were purchased from Adamas, J&K, Energy, Sigma-Aldrich, Alfa Aesar, Acros Organics, TCI and used as received unless otherwise stated.

2. Characterization data

(3a) 4-(phenylsulfonyl)benzonitrile (CAS: 28525-13-5)[1]

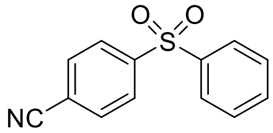

4-(phenylsulfonyl)benzonitrile
Chemical Formula: $C_{13}H_{9}NO_{2}S$
Exact Mass: 243.0354
Molecular Weight: 243.2800

Following the General Procedure with 4-Bromobenzonitrile (36.4 mg, 0.2 mmol, 1.0 equiv), sodium benzenesulfinate (65.6 mg, 0.4 mmol, 2.0 equiv), **3a** was obtained as white solid (41.3 mg, 85%). This product of interest is purified by silica gel flash chromatography (PE: EA = 5:1).

^{1}H NMR (400 MHz, $CDCl_3$) δ 8.05 (d, *J* = 8.4 Hz, 2H), 7.94 (d, *J* = 7.6 Hz, 2H), 7.79 (d, *J* = 8.0 Hz, 2H), 7.61 (d, *J* = 7.2 Hz, 1H), 7.54 (t, *J* = 7.6 Hz, 2H).

^{13}C NMR (101 MHz, $CDCl_3$) δ 145.9, 140.2, 134.1, 133.1, 129.7, 128.3, 128.0, 117.2, 116.9.

(3b) (4-(phenylsulfonyl)phenyl)ethan-1-one (CAS: 65085-83-8) [1]

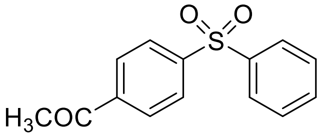

1-(4-(phenylsulfonyl)phenyl)ethan-1-one
Chemical Formula: $C_{14}H_{12}O_{3}S$
Exact Mass: 260.0507
Molecular Weight: 260.3070

Following the General Procedure with 1-(4-bromophenyl)ethan-1-one (39.8 mg, 0.2 mmol, 1.0 equiv), sodium benzenesulfinate (65.6 mg, 0.4 mmol, 2.0 equiv), **3b** was obtained as white solid (41.6 mg, 80%). This product of interest is purified by silica gel flash chromatography (PE: EA = 5:1).

^{1}H NMR (400 MHz, $CDCl_3$) δ 8.05 (s, 4H), 7.96 (d, *J* = 8.0 Hz, 2H), 7.59 (d, *J* = 6.0 Hz, 1H), 7.54 (d, *J* = 6.8 Hz, 2H), 2.62 (s, 3H).

^{13}C NMR (101 MHz, $CDCl_3$) δ 196.7, 145.4, 140.8, 140.4, 133.7, 129.5, 129.1, 128.0, 127.9, 26.9.

(3c) methyl 4-(phenylsulfonyl)benzoate (CAS: 38337-00-7) [1]

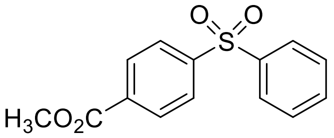

methyl 4-(phenylsulfonyl)benzoate
Chemical Formula: $C_{14}H_{12}O_{4}S$
Exact Mass: 276.0456
Molecular Weight: 276.3060

Following the General Procedure with methyl 4-bromobenzoate (42.8 mg, 0.2 mmol, 1.0 equiv), sodium benzenesulfinate (65.6 mg, 0.4 mmol, 2.0 equiv), **3c** was obtained as white solid (22.1 mg, 40%). This product of interest is purified by silica gel flash chromatography (PE: EA = 5:1).

^{1}H NMR (400 MHz, $CDCl_3$) δ 8.14 (d, *J* = 8.4 Hz, 2H), 8.00 (d, *J* = 8.5 Hz, 2H), 7.95 (d, *J* = 7.3 Hz, 2H), 7.58 (d, *J* = 7.2 Hz, 1H), 7.52 (t, *J* = 6.8 Hz, 2H), 3.92 (s, 3H).

^{13}C NMR (101 MHz, $CDCl_3$) δ 165.5, 145.5, 140.8, 134.3, 133.6, 130.5, 129.5, 127.8, 127.7, 52.7.

(3d) (4-(phenylsulfonyl)phenyl)propan-1-one (CAS: 69567-00-6)

Following the General Procedure with methyl 1-(4-bromophenyl)propan-1-one (45.6 mg, 0.2 mmol, 1.0 equiv), sodium benzenesulfinate (65.6 mg, 0.4 mmol, 2.0 equiv), **3d** was obtained as white solid (42.7 mg, 78%). This product of interest is purified by silica gel flash chromatography (PE: EA = 5:1).

^{1}H NMR (400 MHz, $CDCl_3$) δ 8.02 (d, *J* = 5.2 Hz, 4H), 7.94 (d, *J* = 8.4 Hz, 2H), 7.56 (d, *J* = 7.2 Hz, 1H), 7.50 (t, *J* = 7.6 Hz, 2H), 2.97 (d, *J* = 7.2 Hz, 2H), 1.18 (s, 3H).

^{13}C NMR (101 MHz, $CDCl_3$) δ 199.5, 145.2, 140.8, 140.3, 133.7, 129.5, 128.8, 128.0, 127.8, 32.3, 8.0.

HRMS (ESI) m/z calcd for $C_{15}H_{15}O_3S^+$ M+H $^+$ 275.3415, found 275.3415.

IR: 3070, 2978, 2935, 1697, 1582, 1448, 1155, 730, 600.

Melting point (° C): 57.9-59.1

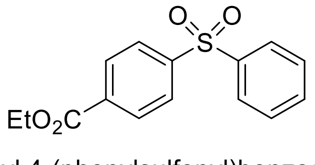

ethyl 4-(phenylsulfonyl)benzoate
Chemical Formula: $C_{15}H_{14}O_4S$
Exact Mass: 290.0613
Molecular Weight: 290.3330

(3e) ethyl 4-(phenylsulfonyl)benzoate (CAS: 101094-06-8)[1]

Following the General Procedure with ethyl 4-bromobenzoate (45.8 mg, 0.2 mmol, 1.0 equiv), sodium benzenesulfinate (65.6 mg, 0.4 mmol, 2.0 equiv), **3e** was obtained as white solid (24.4 mg, 42%). This product of interest is purified by silica gel flash chromatography (PE: EA = 5:1).

^{1}H NMR (400 MHz, $CDCl_3$) δ 8.15 (d, *J* = 8.0 Hz, 2H), 8.00 (d, *J* = 8.0 Hz, 2H), 7.95 (d, *J* = 8.0 Hz, 2H), 7.58 (d, *J* = 7.2 Hz, 1H), 7.52 (d, *J* = 7.6 Hz, 2H), 4.38 (d, *J* = 7.2 Hz, 2H), 1.38 (s, 3H).

^{13}C NMR (101 MHz, $CDCl_3$) δ 165.0, 145.4, 140.9, 134.7, 133.6, 130.4, 129.5, 129.3, 127.8, 127.7, 61.7, 14.2.

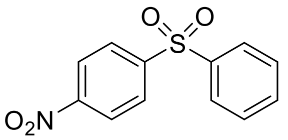

1-nitro-4-(phenylsulfonyl)benzene
Chemical Formula: $C_{12}H_9NO_4S$
Exact Mass: 263.0252
Molecular Weight: 263.2670

(3f) nitro-4-(phenylsulfonyl)benzene (CAS: 1146-39-0)[1]

Following the General Procedure with methyl 1-bromo-4-nitrobenzene (40.0 mg, 0.2 mmol, 1.0 equiv), sodium benzenesulfinate (65.6 mg, 0.4 mmol, 2.0 equiv), **3f** was obtained as white solid (38.3 mg, 73%). This product of interest is purified by silica gel flash chromatography (PE: EA = 5:1).

^{1}H NMR (400 MHz, $CDCl_3$) δ 8.33 (d, *J* = 8.8 Hz, 2H), 8.13 (d, *J* = 6.7 Hz, 2H), 7.97 (d, *J* = 7.9 Hz, 2H), 7.62 (d, *J* = 6.4 Hz, 1H), 7.56 (d, *J* = 6.9 Hz, 2H).

^{13}C NMR (101 MHz, $CDCl_3$) δ 150.4, 147.4, 140.0, 134.2, 129.7, 129.0, 128.1, 124.6.

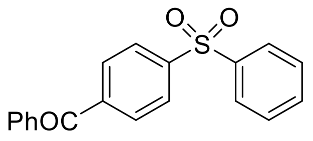

phenyl(4-(phenylsulfonyl)phenyl)methanone
Chemical Formula: $C_{19}H_{14}O_3S$
Exact Mass: 322.0664
Molecular Weight: 322.3780

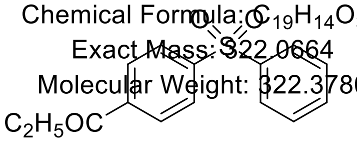

1-(4-(phenylsulfonyl)phenyl)propan-1-one
Chemical Formula: $C_{15}H_{14}O_3S$
Exact Mass: 274.0664
Molecular Weight: 274.3340

(3g) phenyl(4-(phenylsulfonyl)phenyl)methanone (CAS: 54687-39-7) [2]

Following the General Procedure with (4-bromophenyl)(phenyl)methanone (52.0 mg, 0.2 mmol, 1.0 equiv), sodium benzenesulfinate (65.6 mg, 0.4 mmol, 2.0 equiv), **3g** was obtained as white solid (32.8 mg, 51%). This product of interest

is purified by silica gel flash chromatography (PE: EA = 5:1).

^{1}H NMR (400 MHz, $CDCl_3$) δ 8.06 (d, *J* = 6.4 Hz, 2H), 7.99 (d, *J* = 7.6 Hz, 2H), 7.88 (d, *J* = 6.8 Hz, 2H), 7.77 (d, *J* = 7.6 Hz, 2H), 7.66 – 7.59 (m, 2H), 7.56 (d, *J* = 6.8 Hz, 2H), 7.49 (s, 2H).

^{13}C NMR (101 MHz, $CDCl_3$) δ 195.2, 144.7, 141.7, 140.9, 136.4, 133.7, 133.3, 130.5, 130.1, 129.5, 128.6, 127.9, 127.7.

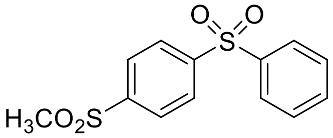

1-(methylsulfonyl)-4-(phenylsulfonyl)benzene
Chemical Formula: $C_{13}H_{12}O_4S_2$
Exact Mass: 296.0177
Molecular Weight: 296.3550

(3h) (methylsulfonyl)-4-(phenylsulfonyl)benzene (CAS: 3112-84-3) [3]

Following the General Procedure with 1-bromo-4-(methylsulfonyl)benzene (46.4 mg, 0.2 mmol, 1.0 equiv), sodium benzenesulfinate (65.6 mg, 0.4 mmol, 2.0 equiv), **3h** was obtained as white solid (26.6 mg, 45%). This product of interest is purified by silica gel flash chromatography (PE: EA = 5:1).

^{1}H NMR (400 MHz, $CDCl_3$) δ 8.14 (d, *J* = 8.4 Hz, 2H), 8.08 (d, *J* = 8.4 Hz, 2H), 7.97 (d, *J* = 7.2 Hz, 2H), 7.63 (d, *J* = 6.8 Hz, 1H), 7.57 (d, *J* = 8.0 Hz, 2H), 3.07 (s, 3H).

^{13}C NMR (101 MHz, $CDCl_3$) δ 146.8, 144.8, 140.2, 134.1, 129.7, 128.7, 128.5, 128.0, 44.3.

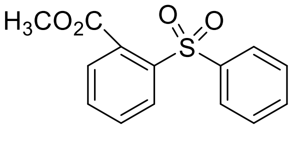

methyl 2-(phenylsulfonyl)benzoate
Chemical Formula: $C_{14}H_{12}O_4S$
Exact Mass: 276.0456
Molecular Weight: 276.3060

(3i) methyl 2-(phenylsulfonyl)benzoate (CAS: 67373-14-2) [1]

Following the General Procedure with methyl 2-iodobenzoate (52.4 mg, 0.2 mmol, 1.0 equiv), sodium benzenesulfinate (65.6 mg, 0.4 mmol, 2.0 equiv), **3i** was obtained as white solid (35.3 mg, 64%). This product of interest is purified by silica gel flash chromatography (PE: EA = 5:1).

^{1}H NMR (400 MHz, $CDCl_3$) δ 8.15 (m, 1H), 7.97 (d, *J* = 7.2 Hz, 2H), 7.62 (m, 2H), 7.57 (m, 2H), 7.51 (t, *J* = 7.4 Hz, 2H), 3.92 (s, 3H).

^{13}C NMR (101 MHz, $CDCl_3$) δ 167.7, 141.5, 139.0, 133.3, 130.9, 130.2, 129.2, 129.0, 127.8, 53.0.

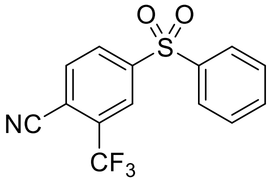

4-(phenylsulfonyl)-2-(trifluoromethyl)benzonitrile
Chemical Formula: $C_{14}H_8F_3NO_2S$
Exact Mass: 311.0228
Molecular Weight: 311.2782

(3j) 4-(phenylsulfonyl)-2-(trifluoromethyl)benzonitrile (CAS: 2383030-86-0) [1]

Following the General Procedure with 4-bromo-2-(trifluoromethyl)benzonitrile (49.8 mg, 0.2 mmol, 1.0 equiv), sodium benzenesulfinate (65.6 mg, 0.4 mmol, 2.0 equiv), **3j** was obtained as white solid (51.0 mg, 82%). This product of interest is purified by silica gel flash chromatography (PE: EA = 5:1).

^{1}H NMR (400 MHz, $CDCl_3$) δ 8.34 (s, 1H), 8.25 (d, *J* = 8.0 Hz, 1H), 8.04 – 7.95 (m, 3H), 7.68 (t, *J* = 7.4 Hz, 1H), 7.59 (t, *J* = 7.6 Hz, 2H).

^{13}C NMR (101 MHz, $CDCl_3$) δ 146.6, 139.3, 135.9, 134.6, 134.2 (d, *J* = 34.0 Hz), 131.3, 130.0, 128.2, 125.8 (q, *J* = 4.7 Hz), 121.5(d, *J* = 275.8 Hz), 114.5, 114.0.

^{19}F NMR (376 MHz, $CDCl_3$) δ -62.10 (s).

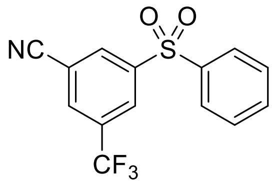

3-(phenylsulfonyl)-5-(trifluoromethyl)benzonitrile
Chemical Formula: $C_{14}H_8F_3NO_2S$
Exact Mass: 311.0228
Molecular Weight: 311.2782

(3k) 3-(phenylsulfonyl)-5-(trifluoromethyl)benzonitrile[1]
Following the General Procedure with 3-bromo-5-(trifluoromethyl)benzonitrile (40.0 mg, 0.2 mmol, 1.0 equiv), sodium benzenesulfinate (65.6 mg, 0.4 mmol, 2.0 equiv), **3k** was obtained as white solid (34.8 mg, 56%). This product of interest is purified by silica gel flash chromatography (PE: EA = 5:1).

^{1}H NMR (400 MHz, $CDCl_3$) δ 8.40 (d, J = 10.4 Hz, 2H), 8.08 (s, 1H), 7.99 (d, J = 8.4 Hz, 2H), 7.68 (m, 1H), 7.60 (m, 2H).

^{13}C NMR (101 MHz, $CDCl_3$) δ 145.1, 139.3, 134.6, 134.3, 133.5 (d, J = 35.0 Hz), 133.0 (d, J = 3.6 Hz), 130.2, 128.3 (q, J = 3.6), 128.2, 127.7, 122.0 (d, J = 274.8 Hz), 115.7, 115.2.

^{19}F NMR (376 MHz, $CDCl_3$) δ -63.06 (s).

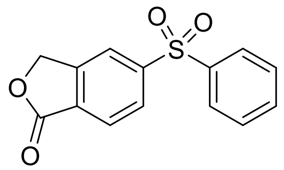

5-(phenylsulfonyl)isobenzofuran-1(3*H*)-one
Chemical Formula: $C_{14}H_{10}O_4S$
Exact Mass: 274.0300
Molecular Weight: 274.2900

(3l) 5-(phenylsulfonyl)isobenzofuran-1(3H)-one (CAS: 2232133-50-3)[3]
Following the General Procedure with 5-bromoisobenzofuran-1(3H)-one (42.4 mg, 0.2 mmol, 1.0 equiv), sodium benzenesulfinate (65.6 mg, 0.4 mmol, 2.0 equiv), **3l** was obtained as white solid (27.4 mg, 50%). This product of interest is purified by silica gel flash chromatography (PE: EA = 5:1)

^{1}H NMR (400 MHz, $CDCl_3$) δ 8.14 (s, 1H), 8.09 (d, J = 8.0 Hz, 1H), 8.03 (d, J = 8.0 Hz, 1H), 7.99 – 7.96 (m, 2H), 7.64 (m, 1H), 7.55 (t, J = 7.8 Hz, 2H), 5.39 (s, 2H).

^{13}C NMR (101 MHz, $CDCl_3$) δ 169.1, 147.3, 147.2, 140.3, 134.0, 129.8, 129.7, 128.6, 128.0, 126.9, 122.0, 69.6.

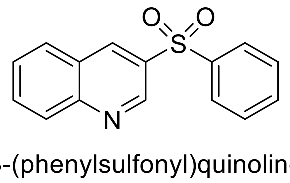

3-(phenylsulfonyl)quinoline
Chemical Formula: $C_{15}H_{11}NO_2S$
Exact Mass: 269.0510
Molecular Weight: 269.3180

(3m) 3-(phenylsulfonyl)quinoline (CAS: 117620-35-6)[1]
Following the General Procedure with methyl 1-bromo-4-nitrobenzene (40.0 mg, 0.2 mmol, 1.0 equiv), sodium benzenesulfinate (65.6 mg, 0.4 mmol, 2.0 equiv), **3m** was obtained as white solid (22.6 mg, 42%). This product of interest is purified by silica gel flash chromatography (PE: EA = 5:1)

^{1}H NMR (400 MHz, $CDCl_3$) δ 9.28 (d, J = 2.0 Hz, 1H), 8.83 (d, J = 2.0 Hz, 1H), 8.16 (d, J = 8.4 Hz, 1H), 8.07 – 8.01 (m, 2H), 7.97 (d, J = 8.4 Hz, 1H), 7.87 (m, 1H), 7.68 (m, 1H), 7.63 – 7.57 (m, 1H), 7.54 (m, 2H).

^{13}C NMR (101 MHz, $CDCl_3$) δ 149.4, 147.1, 141.0, 137.0, 134.7, 133.8, 132.8, 129.6, 129.2, 128.4, 127.8, 126.4.

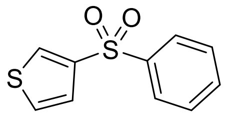

3-(phenylsulfonyl)thiophene
Chemical Formula: $C_{10}H_8O_2S_2$
Exact Mass: 223.9966
Molecular Weight: 224.2920

(3n) 3-(phenylsulfonyl)thiophene (CAS: 89770-30-9) [4]

Following the General Procedure with 3-iodothiophene (40.0 mg, 0.2 mmol, 1.0 equiv), sodium benzenesulfinate (44.6 mg, 0.4 mmol, 2.0 equiv), **3n** was obtained as white solid (25.0 mg, 56%). This product of interest is purified by silica gel flash chromatography (PE: EA = 5:1)

^{1}H NMR (400 MHz, $CDCl_3$) δ 8.13 – 8.08 (m, 1H), 7.97 (d, *J* = 7.2 Hz, 2H), 7.59 (t, *J* = 6.8 Hz, 1H), 7.52 (t, *J* = 7.4 Hz, 2H), 7.41 – 7.36 (m, 1H), 7.36 – 7.32 (m, 1H).

^{13}C NMR (101 MHz, $CDCl_3$) δ 142.0, 141.6, 133.3, 131.6, 129.3, 128.4, 127.5, 125.9.

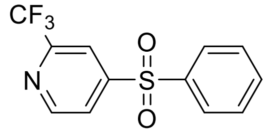

4-(phenylsulfonyl)-2-(trifluoromethyl)pyridine
Chemical Formula: $C_{12}H_8F_3NO_2S$
Exact Mass: 287.0228
Molecular Weight: 287.2562

(3o) 4-(phenylsulfonyl)-2-(trifluoromethyl)pyridine [5] **(CAS: 2412989-04-7)**

Following the General Procedure with 4-bromo-2-(trifluoromethyl)pyridine (44.9 mg, 0.2 mmol, 1.0 equiv), sodium benzenesulfinate (65.6 mg, 0.4 mmol, 2.0 equiv), **3o** was obtained as white solid (46.5 mg, 81%). This product of interest is purified by silica gel flash chromatography (PE: EA = 5:1)

^{1}H NMR (400 MHz, $CDCl_3$) δ 8.93 (d, *J* = 4.8 Hz, 1H), 8.13 (s, 1H), 7.99 (d, *J* = 8.0 Hz, 3H), 7.74 – 7.63 (m, 1H), 7.63 – 7.53 (m, 2H).

^{13}C NMR (101 MHz, $CDCl_3$) δ 152.2, 151.7, 150.0 (d, *J* = 36.1 Hz), 138.8, 134.7, 130.0, 128.3, 123.7, 120.7 (d, *J* = 275.8 Hz), 117.79 (d, *J* = 2.7 Hz).

^{19}F NMR (376 MHz, $CDCl_3$) δ -68.06 (s).

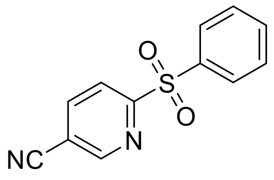

6-(phenylsulfonyl)nicotinonitrile
Chemical Formula: $C_{12}H_8N_2O_2S$
Exact Mass: 244.0306
Molecular Weight: 244.2680

(3p) 6-(phenylsulfonyl)nicotinonitrile (205514-29-0)

Following the General Procedure with 2-Bromo-4-cyanopyridine (36.6 mg, 0.2 mmol, 1.0 equiv), sodium benzenesulfinate (65.6 mg, 0.4 mmol, 2.0 equiv), **3p** was obtained as white solid (20.5 mg, 42%). This product of interest is purified by silica gel flash chromatography (PE: EA = 5:1)

^{1}H NMR (400 MHz, $CDCl_3$) δ 8.89 (d, *J* = 1.1 Hz, 1H), 8.34 (d, *J* = 8.0 Hz, 1H), 8.23 (dd, *J* = 8.4, 2.0 Hz, 1H), 8.06 (d, *J* = 7.2 Hz, 2H), 7.68 (t, *J* = 6.8 Hz, 1H), 7.58 (t, *J* = 7.6 Hz, 2H).

^{13}C NMR (101 MHz, $CDCl_3$) δ 161.7, 152.7, 141.9, 137.5, 134.6, 129.4, 129.4, 121.9, 115.1, 113.0.

HRMS (ESI) m/z calcd for $C_{12}H_9N_2O_2S^+$ M+H $^+$ 245.0371, found 245.0371.

IR: 3065, 2243, 1452, 325, 1161, 628.

Melting point (° C): 119.1-120.1

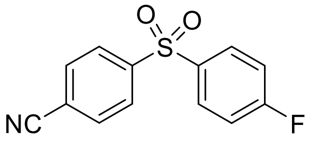

4-((4-fluorophenyl)sulfonyl)benzonitrile
Chemical Formula: $C_{13}H_8FNO_2S$
Exact Mass: 261.0260
Molecular Weight: 261.2704

(3q) 4-((4-fluorophenyl)sulfonyl)benzonitrile[1] (CAS: 1268049-80-4)

Following the General Procedure with 4-Bromobenzonitrile (36.4 mg, 0.2 mmol, 1.0 equiv), 4-Flurobenzenesulfinic acid sodium salt (91.0 mg, 0.4 mmol, 2.0 equiv), **3q** was obtained as white solid (20.8 mg, 40%). This product of interest is purified by silica gel flash chromatography (PE: EA = 5:1)

^{1}H NMR (400 MHz, $CDCl_3$) δ 8.05 (d, *J* = 8.4 Hz, 2H), 8.02 – 7.95 (m, 2H), 7.81 (d, *J* = 8.0 Hz, 2H), 7.24 (dd, *J* = 16.0, 8.0 Hz, 2H).

^{13}C NMR (101 MHz, $CDCl_3$) δ 166.0 (d, *J* = 258.7 Hz), 145.7, 136.2, 133.2, 130.9 (d, *J* = 9.8 Hz), 128.2, 117.2, 117.1 (d, *J* = 3.7 Hz), 117.0.

^{19}F NMR (376 MHz, $CDCl_3$) δ -102.34 (s).

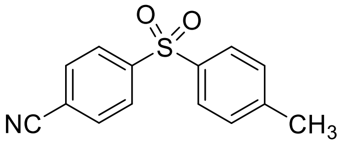

4-tosylbenzonitrile
Chemical Formula: $C_{14}H_{11}NO_2S$
Exact Mass: 257.0510
Molecular Weight: 257.3070

(3r) 4-tosylbenzonitrile[1] (CAS：38111-56-7)

Following the General Procedure with 4-Bromobenzonitrile (36.4 mg, 0.2 mmol, 1.0 equiv), 4-methyl-benzenesulfinicacisodiumsalt (71.2 mg, 0.4 mmol, 2.0 equiv), **3r** was obtained as white solid (27.8 mg, 54%). This product of interest is purified by silica gel flash chromatography (PE: EA = 5:1)

^{1}H NMR (400 MHz, $CDCl_3$) δ 8.04 (d, *J* = 6.8 Hz, 2H), 7.83 (d, *J* = 6.8 Hz, 2H), 7.81 – 7.76 (m, 2H), 7.34 (d, *J* = 7.6 Hz, 2H), 2.42 (s, 3H).

^{13}C NMR (101 MHz, $CDCl_3$) δ 146.3, 145.3, 137.1, 133.1, 130.3, 128.1, 128.0, 117.2, 116.7, 21.7.

3. References

1. Jiang, S.; Zhang, Z.-T.; Young, D. J.; Chai, L.-L.; Wu, Q.; Li, H.-X., Visible-light mediated cross-coupling of aryl halides with sodium sulfinates via carbonyl-photoredox/nickel dual catalysis. *Organic Chemistry Frontiers* **2022,** *9* (5), 1437-1444.

2. Nandi, G. C., An efficient Cu-catalyzed microwave-assisted synthesis of diaryl sulfones. *SyntheticCommunications* **2017,** *47* (4), 319-323.

3. Cabrera-Afonso, M. a. J. s.; Lu, Z.-P.; Kelly, C. B.; Lang, S. B.; Dykstra, R.; Gutierrez,O.; Molander, G. A., Engaging sulfinate salts via Ni/photoredox dual catalysis enables facile Csp2â€"SO2R coupling. *Chemical Science* **2018,** *9* (12), 3186-3191.

4. Liu, N.-W.; Liang, S.; Margraf, N.; Shaaban, S.; Luciano, V.; Drost, M.; Manolikakes, G., Nickel-Catalyzed Synthesis of Diaryl Sulfones from Aryl Halides and sodium Sulfinates. *European Journal of Organic Chemistry* **2018,** *2018* (10), 1208-1210.

5. Zhu, D. L.; Wu, Q.; Li, H. Y.; Li, H. X.; Lang, J. P., Hantzsch Ester as a Visible-Light Photoredox Catalyst for Transition-Metal-Free Coupling of Arylhalides and Arylsulfinates. *Chemistry* **2020,** *26* (16), 3484-3488.

4. Copies of NMR spectra

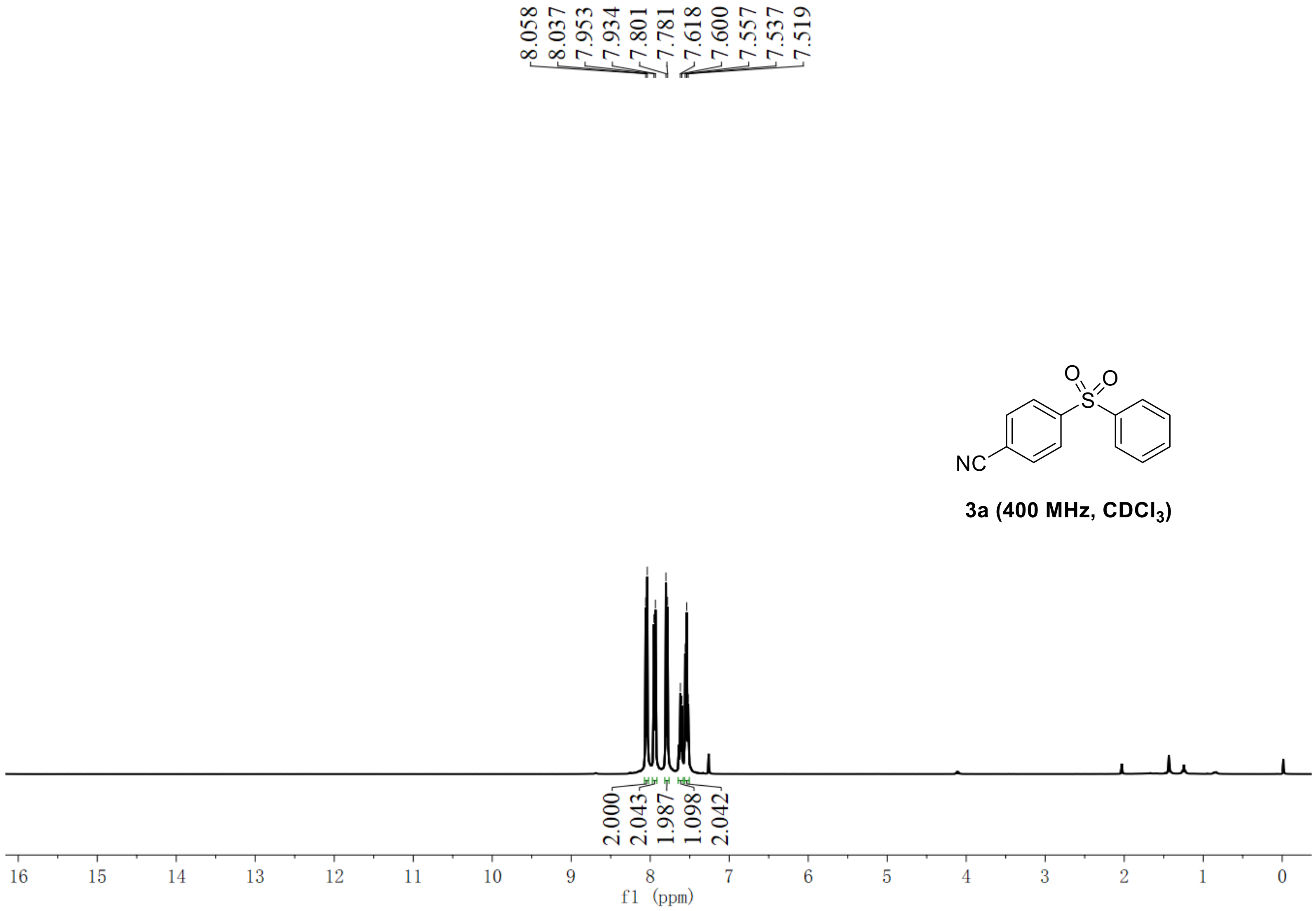

3a (400 MHz, $CDCl_3$)

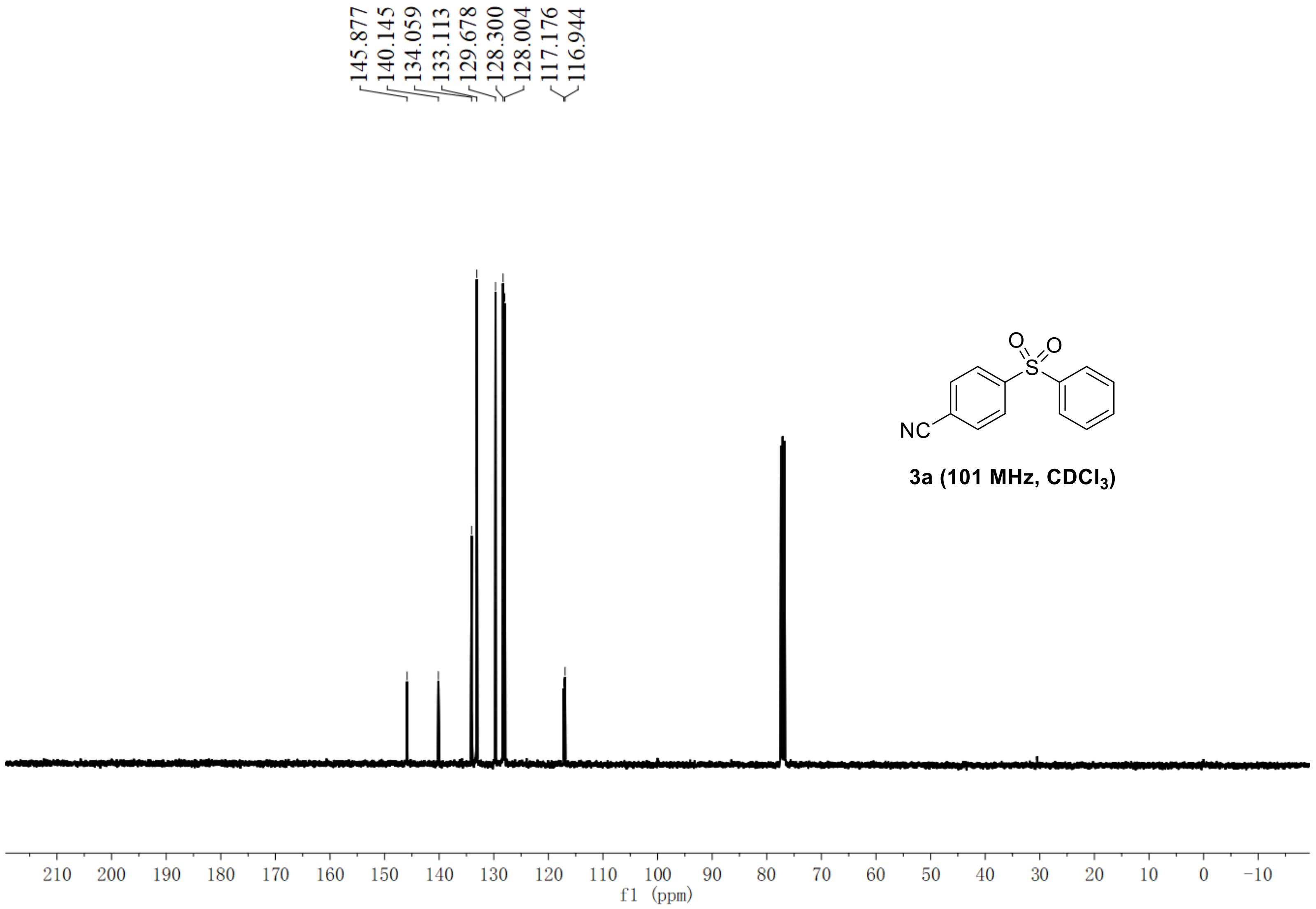
145.877
140.145
134.059
133.113
129.678
128.300
128.004
117.176
116.944
NC
O
S
O
3a (101 MHz, CDCl3)
210 200 190 180 170 160 150 140 130 120 110 100 90 80 70 60 50 40 30 20 10 0 -10
f1 (ppm)

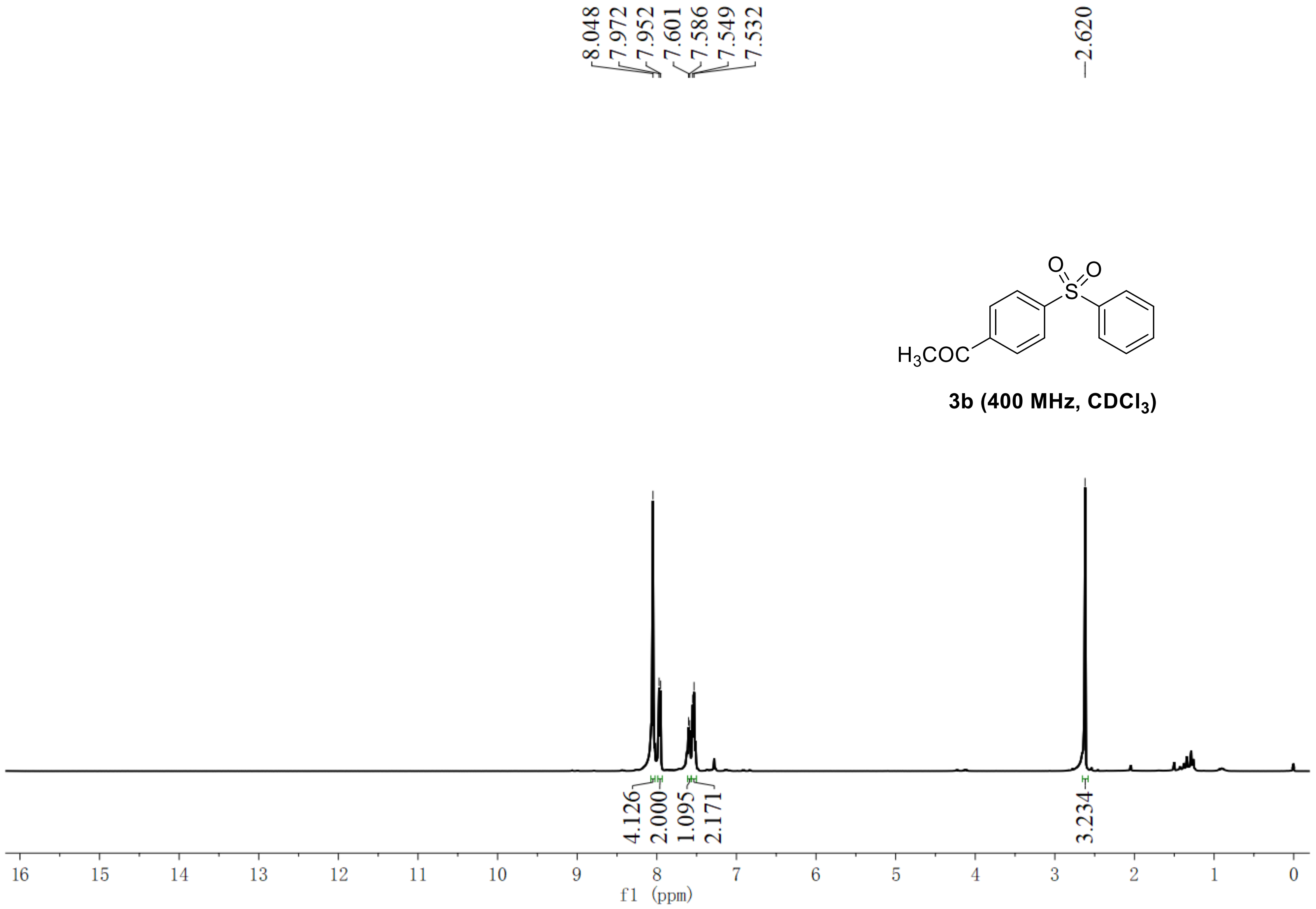

3b (400 MHz, $CDCl_3$)

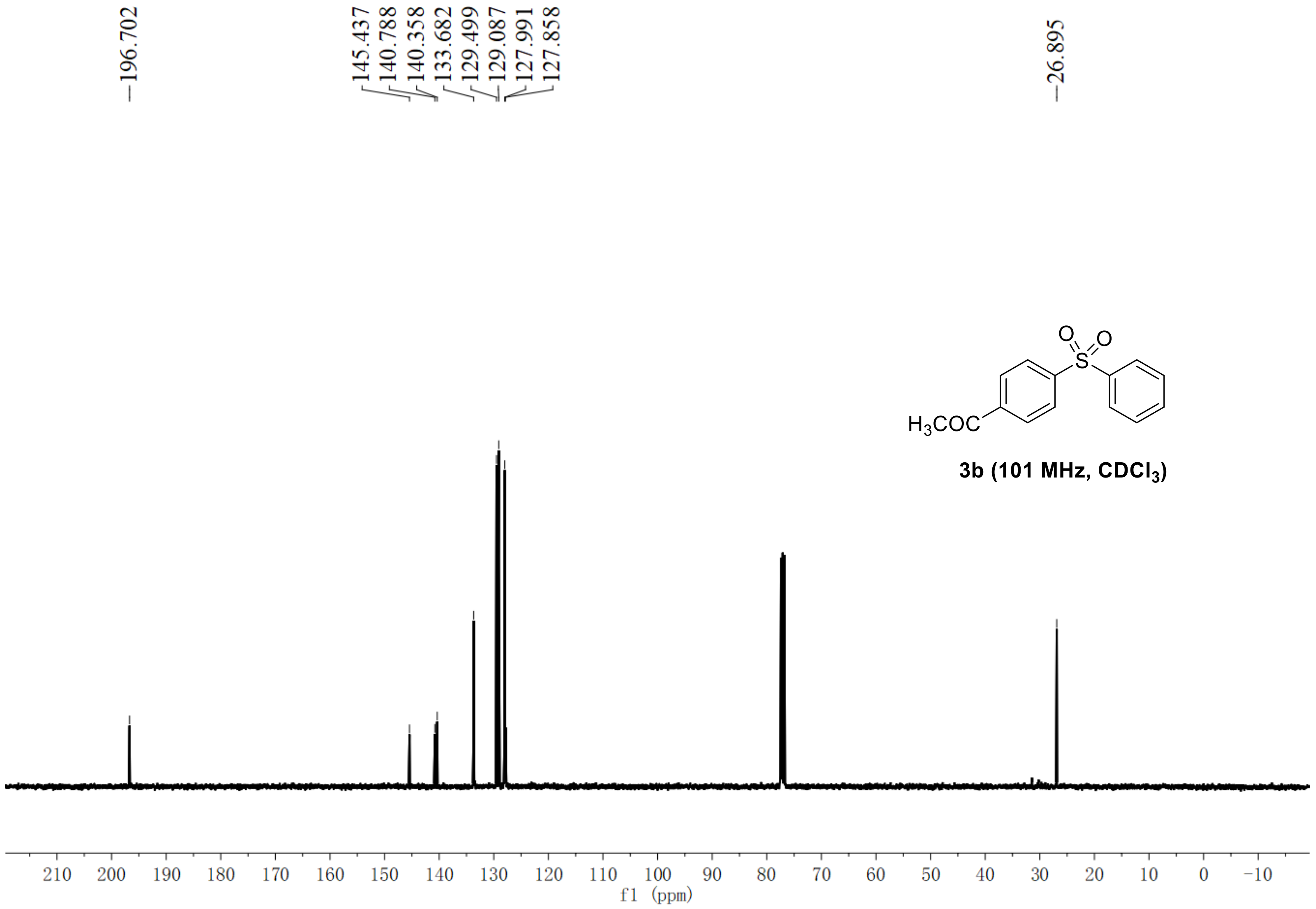

3b (101 MHz, $CDCl_3$)

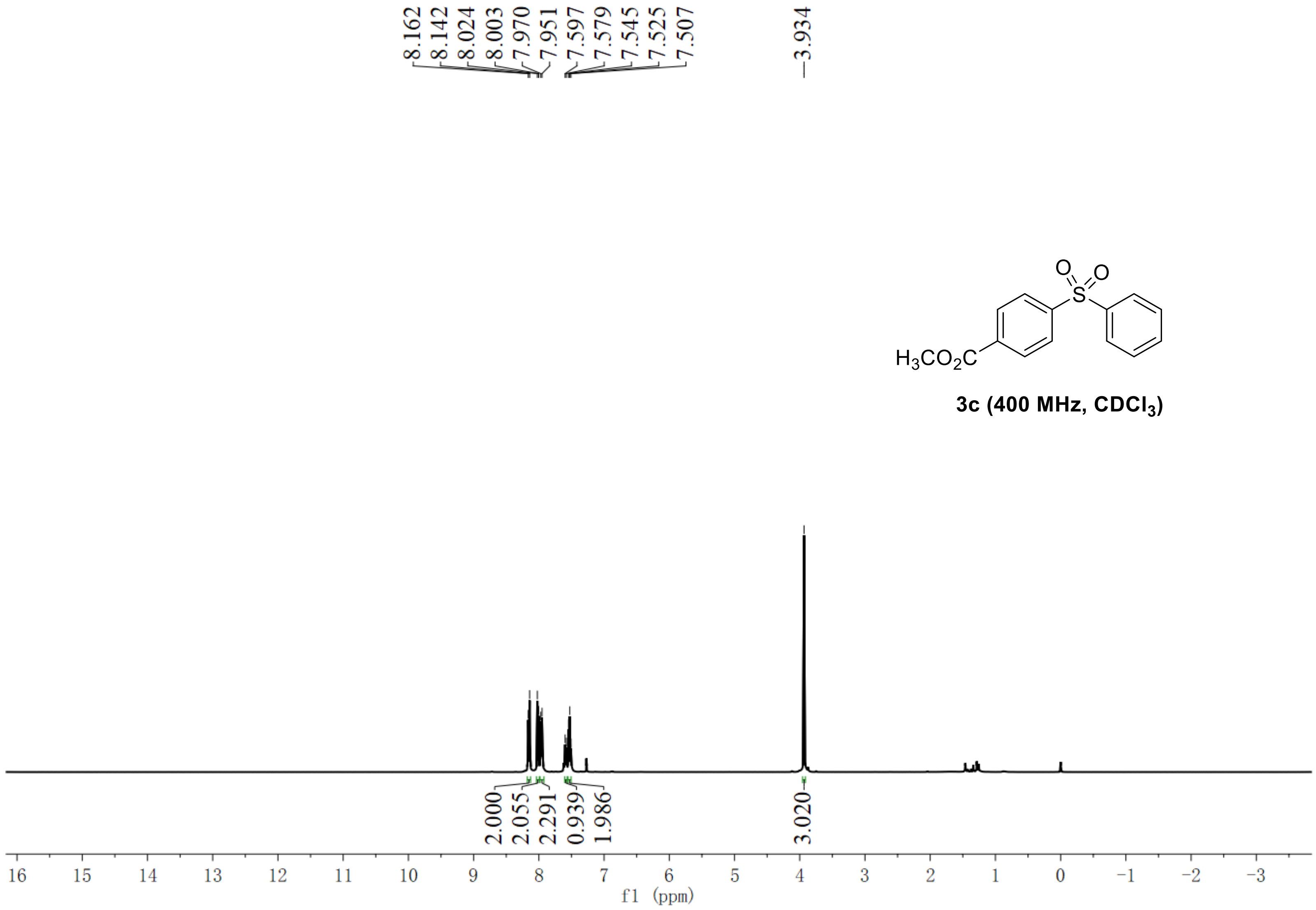

3c (400 MHz, $CDCl_3$)

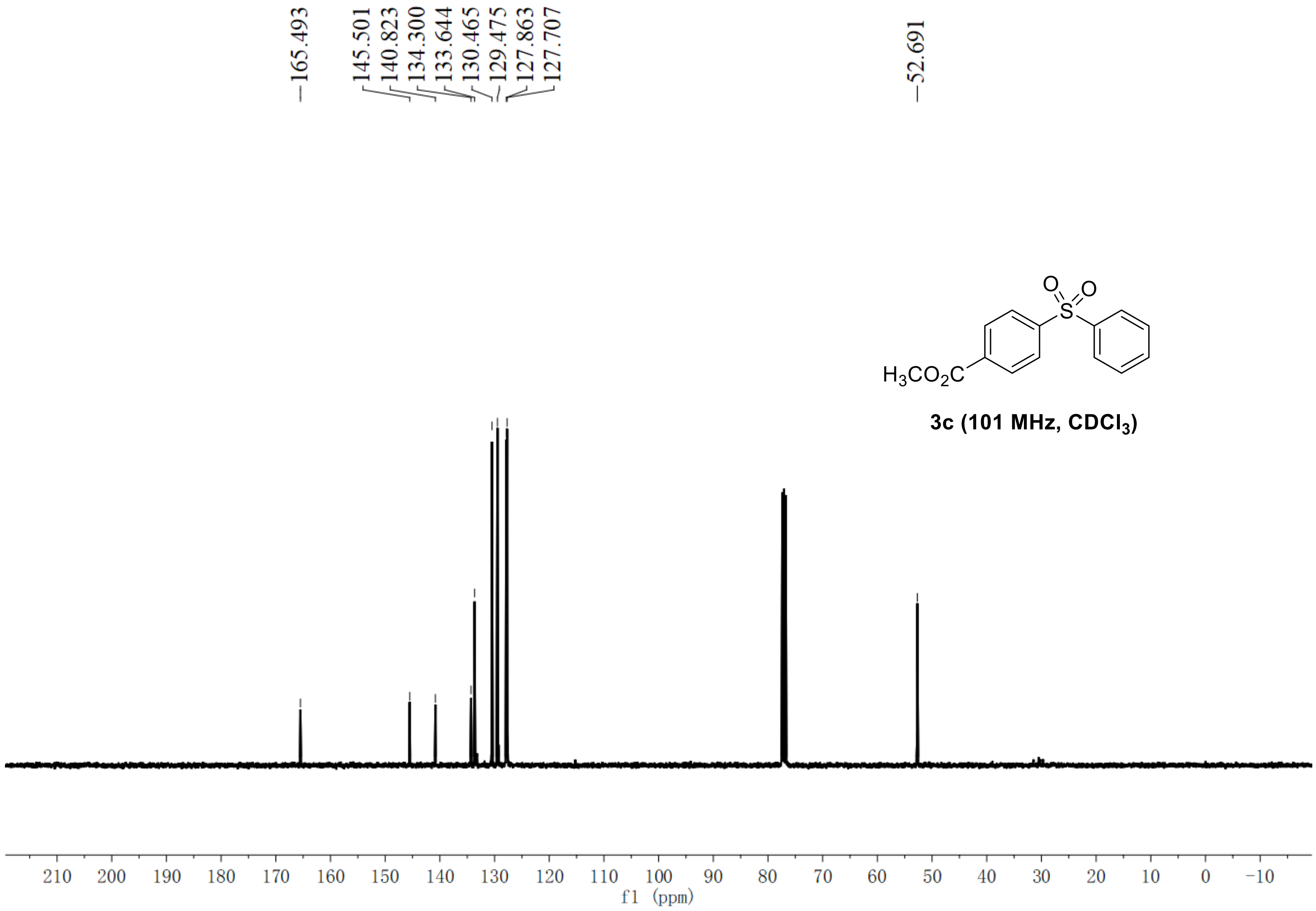

165.493
145.501
140.823
134.300
133.644
130.465
129.475
127.863
127.707
52.691
O
S
O
H_3CO_2C
3c (101 MHz, $CDCl_3$)
210 200 190 180 170 160 150 140 130 120 110 100 90 80 70 60 50 40 30 20 10 0 -10
f1 (ppm)

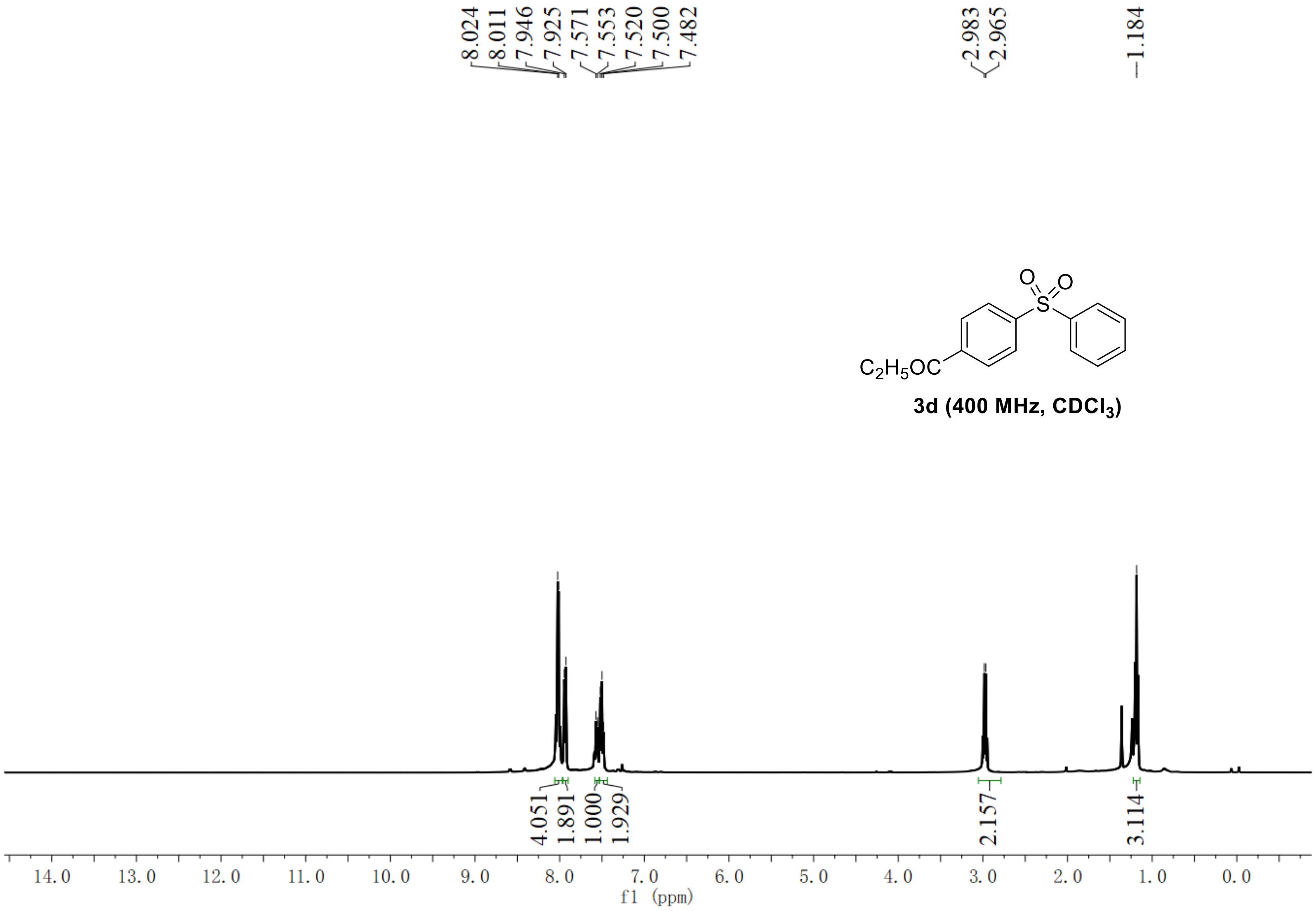

3d (400 MHz, $CDCl_3$)

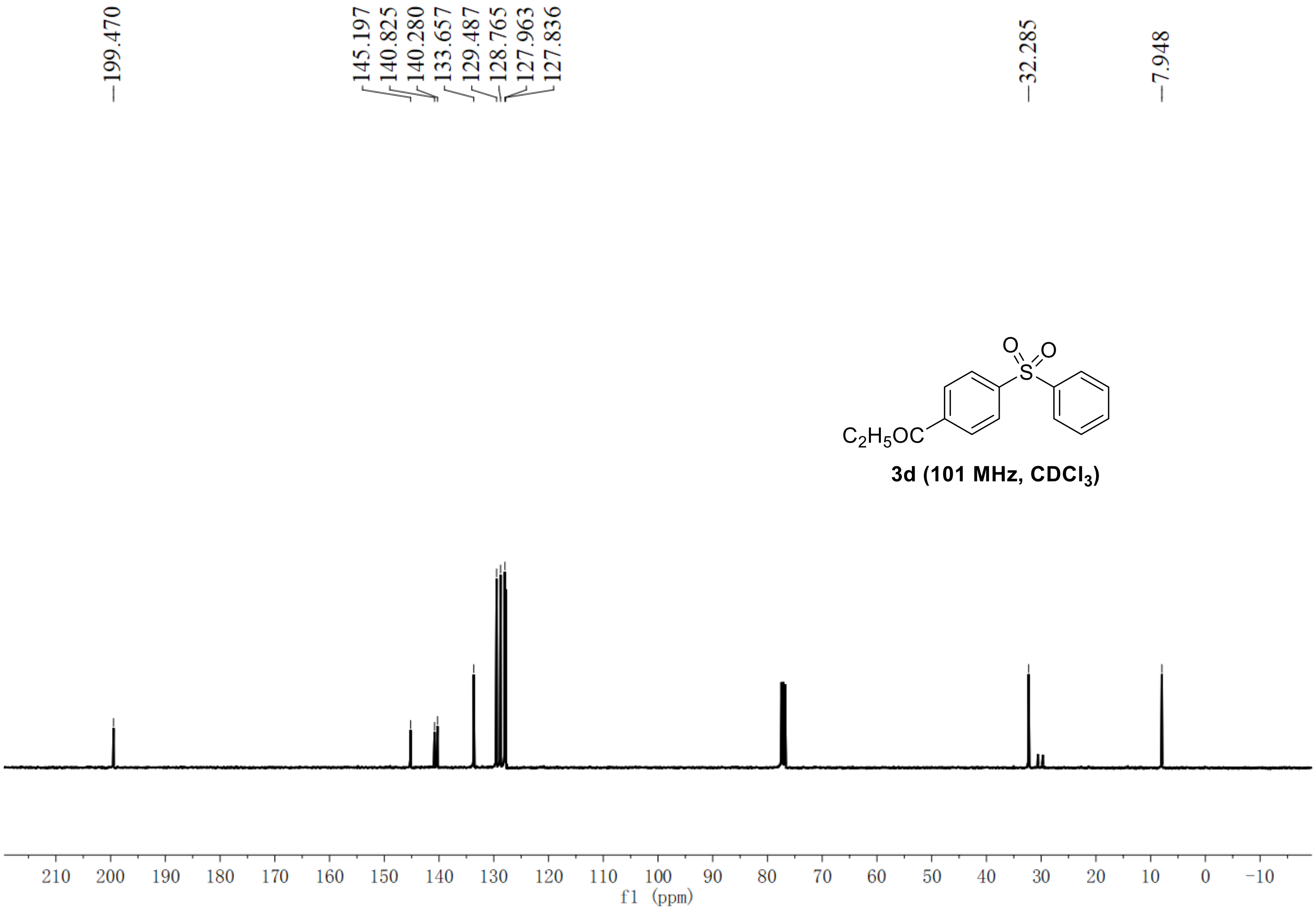
199.470
145.197
140.825
140.280
133.657
129.487
128.765
127.963
127.836
32.285
7.948
C_2H_5OC
O
S
O
3d (101 MHz, $CDCl_3$)
210 200 190 180 170 160 150 140 130 120 110 100 90 80 70 60 50 40 30 20 10 0 −10
f1 (ppm)

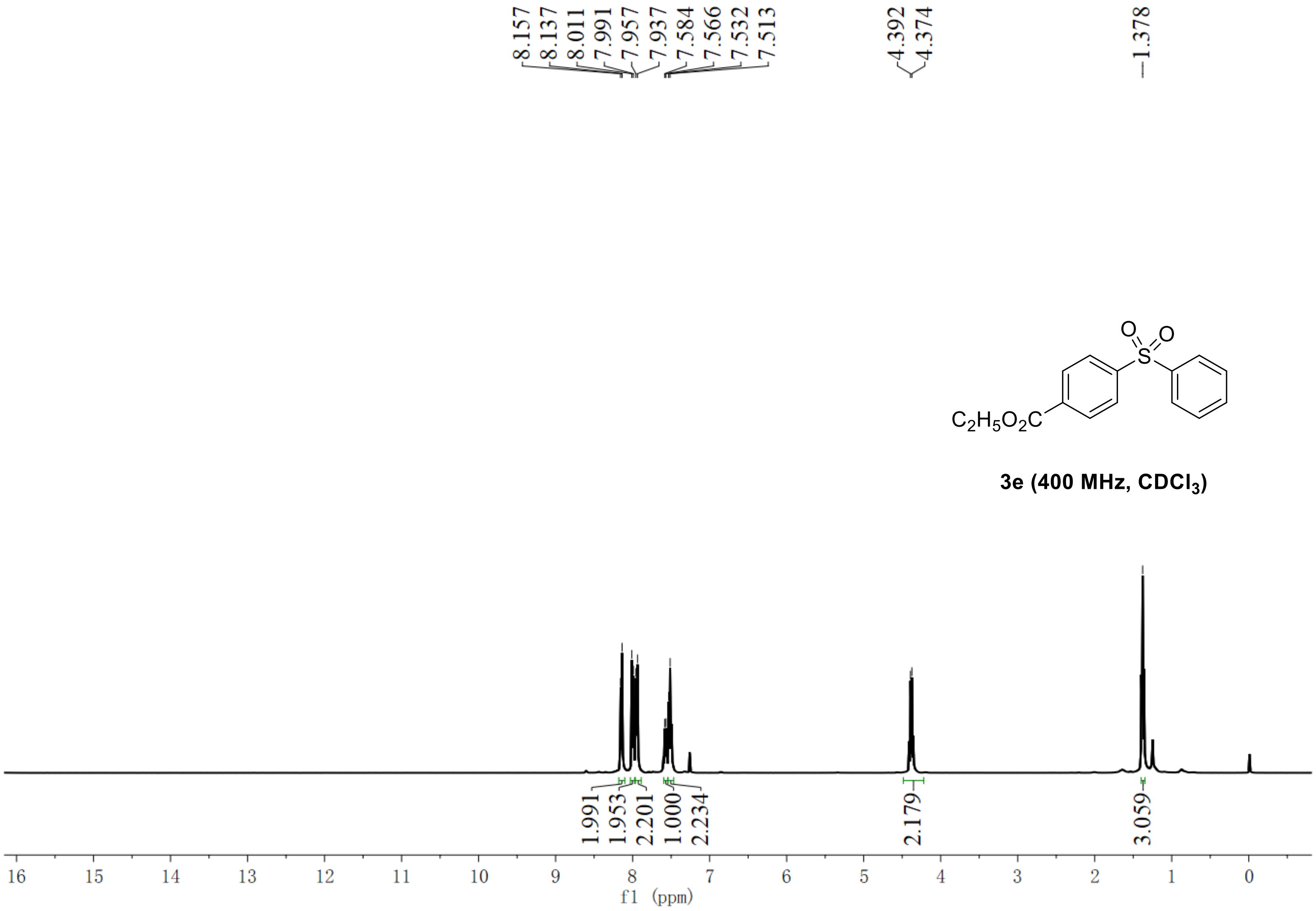

3e (400 MHz, $CDCl_3$)

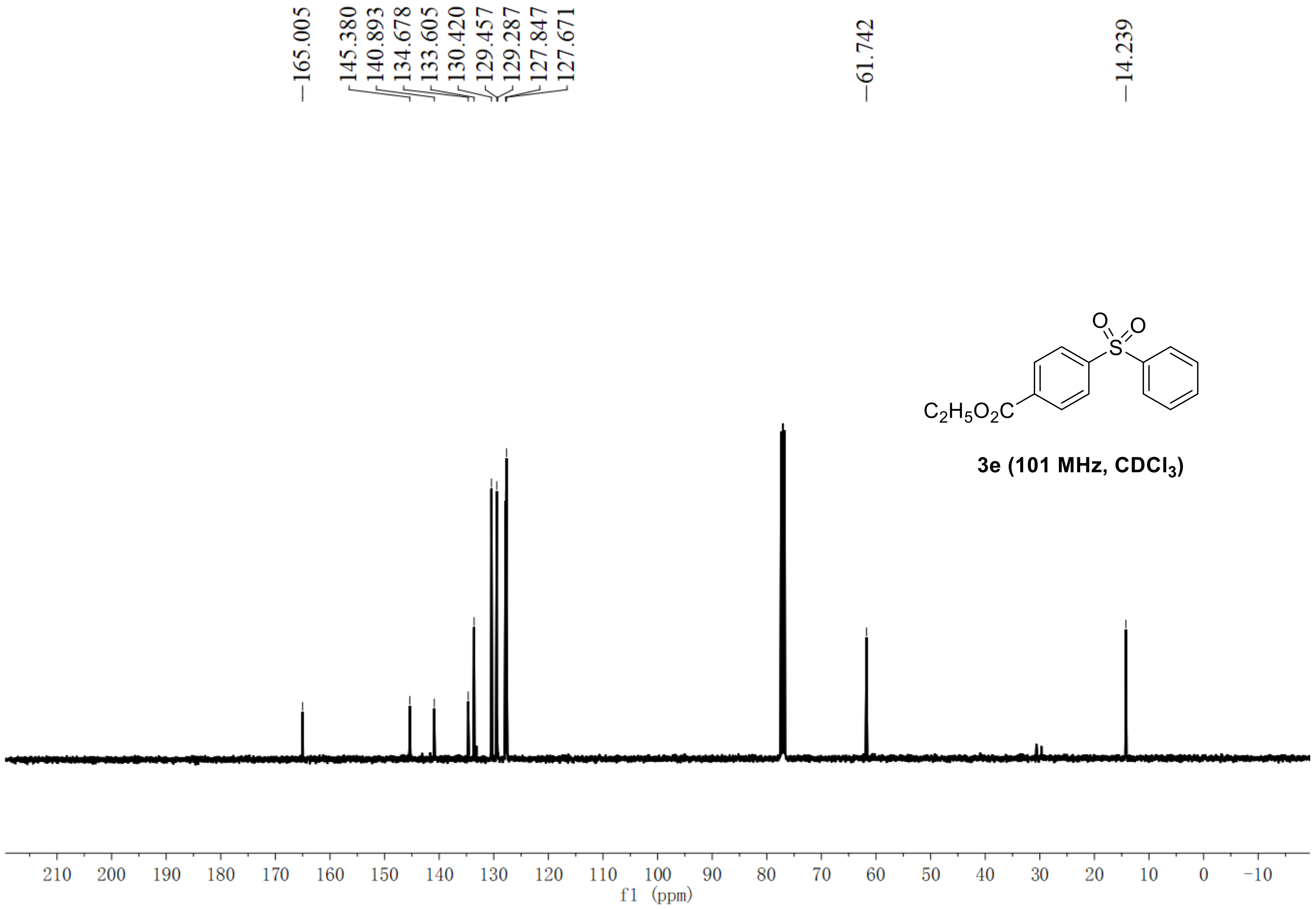

3e (101 MHz, $CDCl_3$)

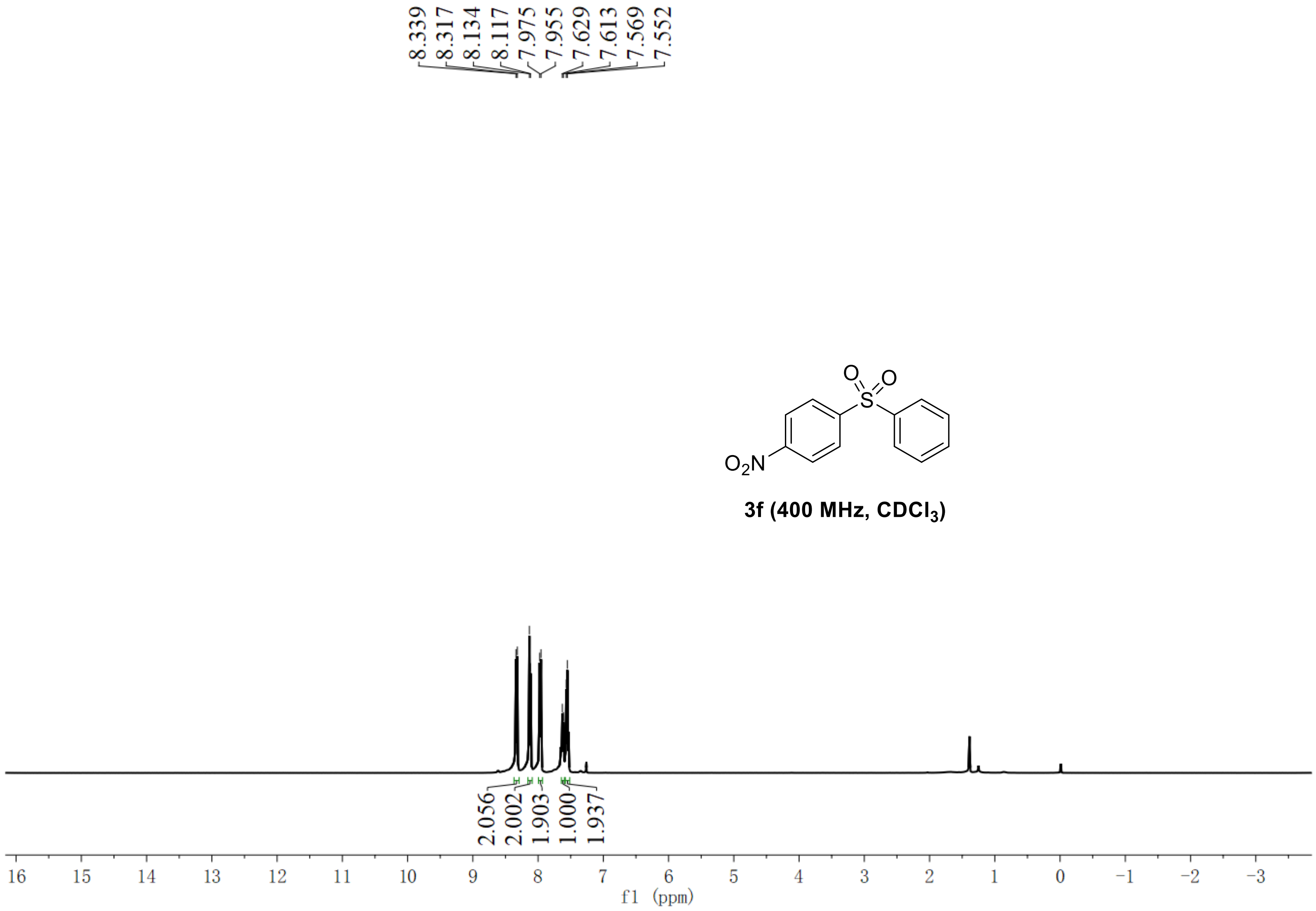

3f (400 MHz, $CDCl_3$)

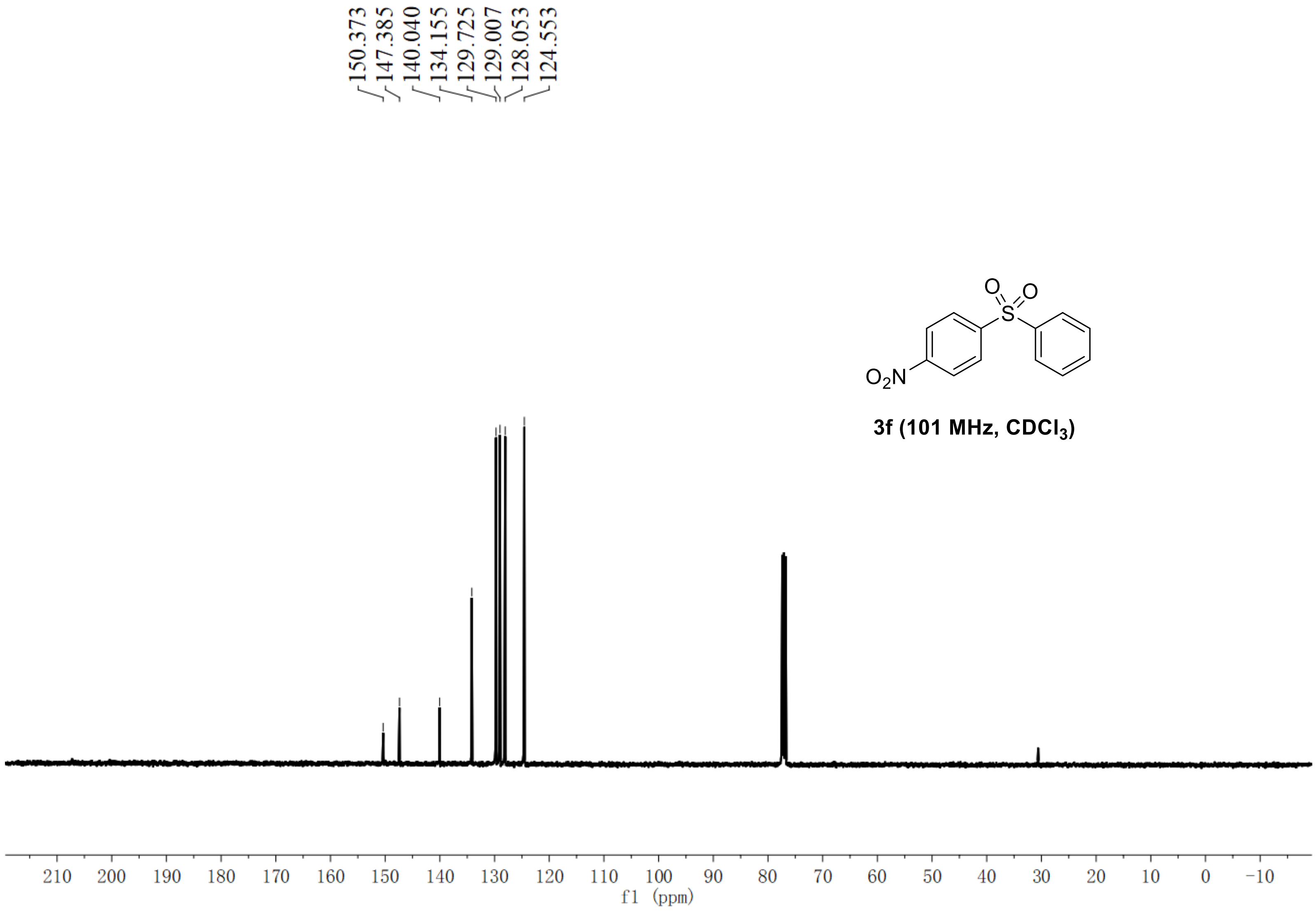
150.373
147.385
140.040
134.155
129.725
129.007
128.053
124.553
O
S
O
O_2N
3f (101 MHz, $CDCl_3$)
210 200 190 180 170 160 150 140 130 120 110 100 90 80 70 60 50 40 30 20 10 0 −10
f1 (ppm)

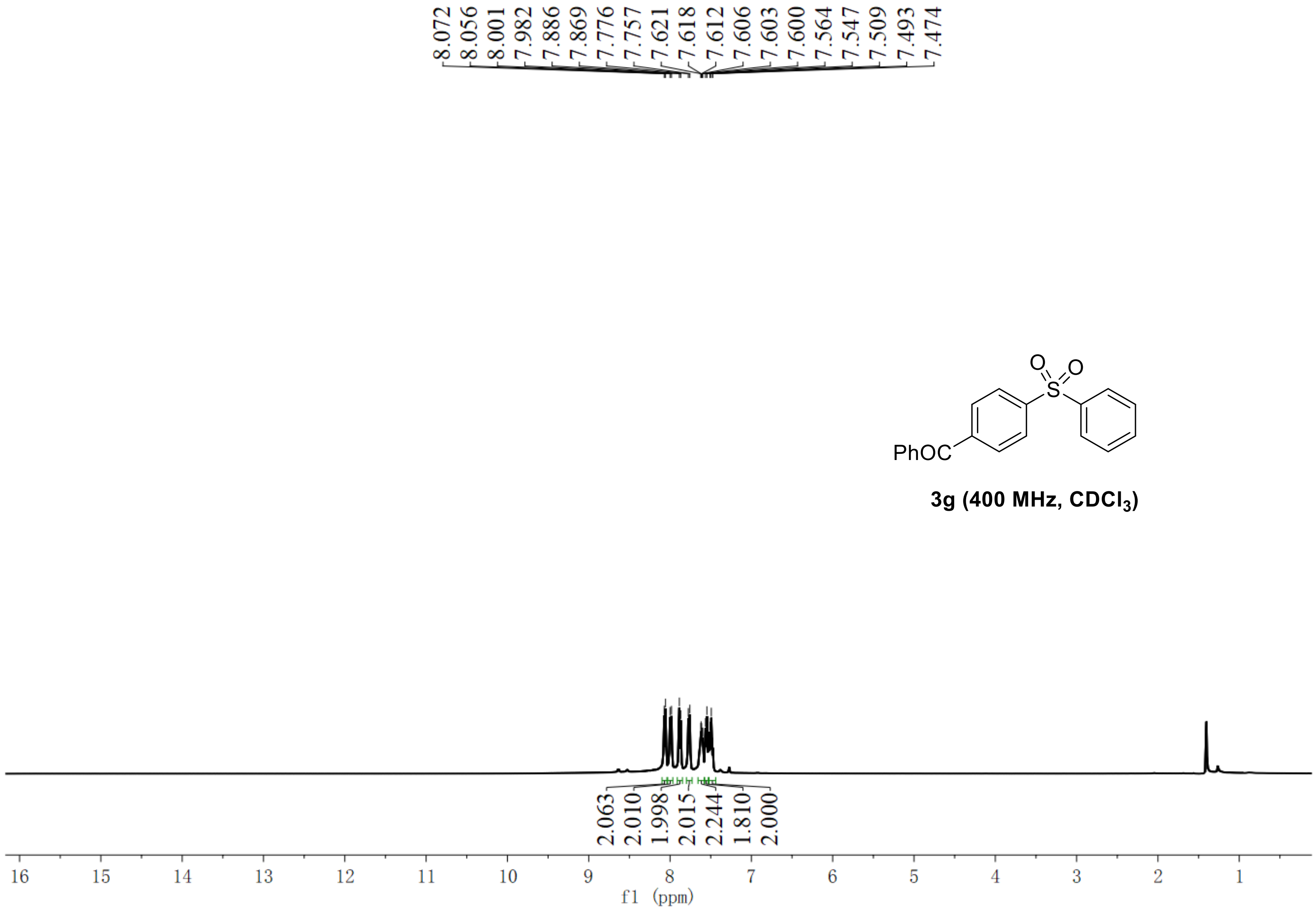

3g (400 MHz, $CDCl_3$)

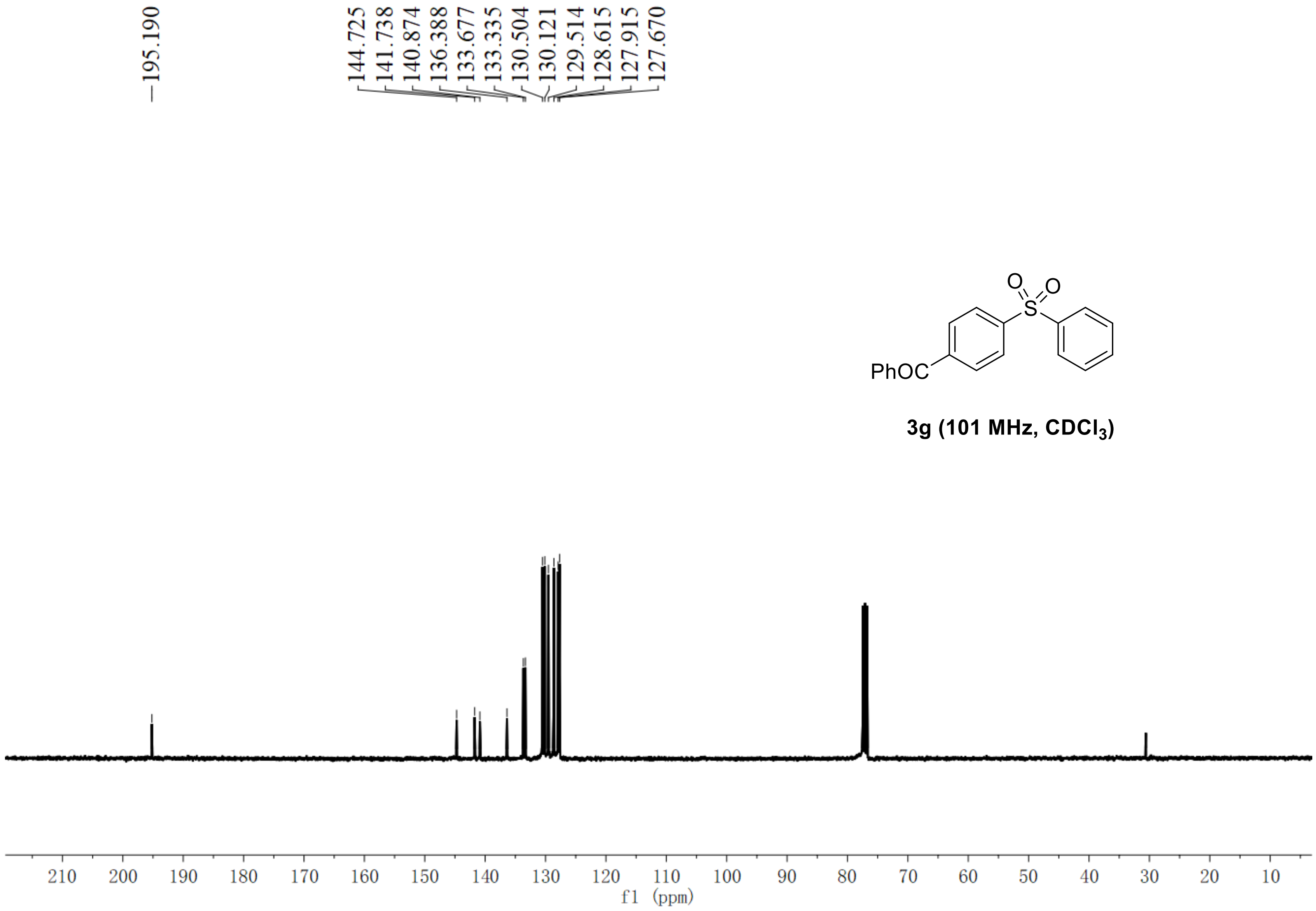

3g (101 MHz, $CDCl_3$)

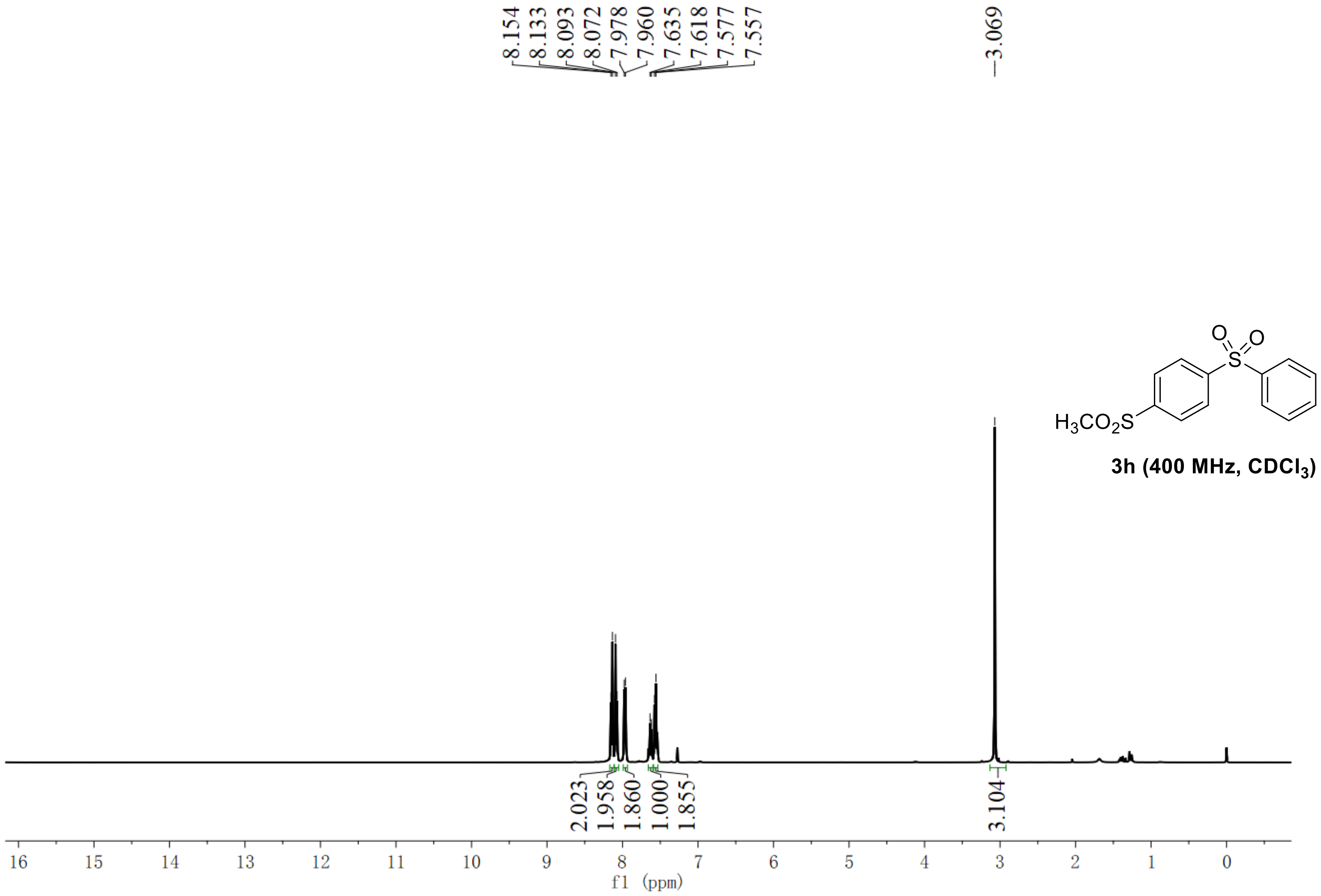
8.154
8.133
8.093
8.072
7.978
7.960
7.635
7.618
7.577
7.557
3.069
H_3CO_2S
O
O
S
3h (400 MHz, $CDCl_3$)
2.023
1.958
1.860
1.000
1.855
3.104
16 15 14 13 12 11 10 9 8 7 6 5 4 3 2 1 0
f1 (ppm)

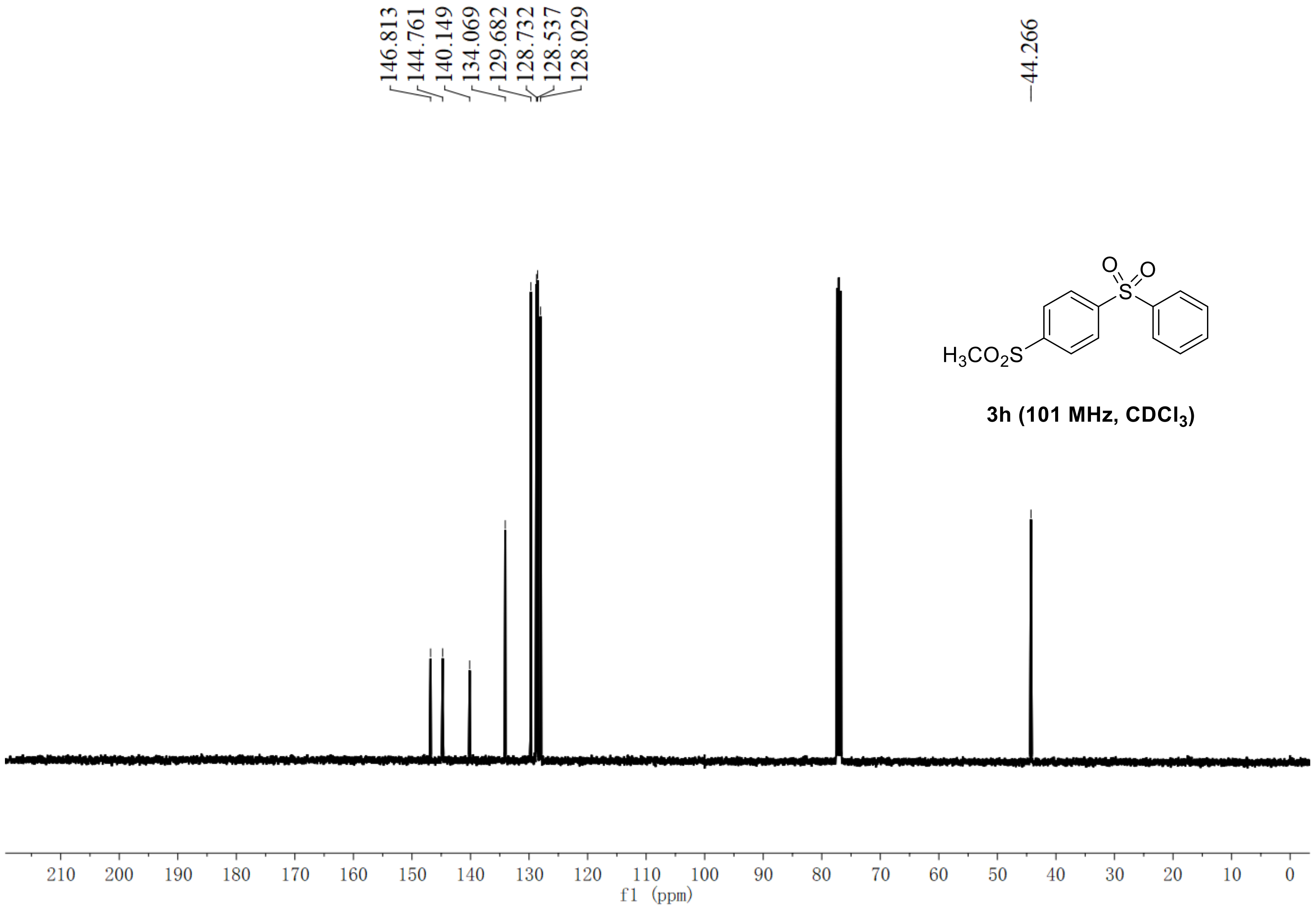
146.813
144.761
140.149
134.069
129.682
128.732
128.537
128.029
44.266
H3CO2S
O
O
S
3h (101 MHz, CDCl3)
210 200 190 180 170 160 150 140 130 120 110 100 90 80 70 60 50 40 30 20 10 0
f1 (ppm)

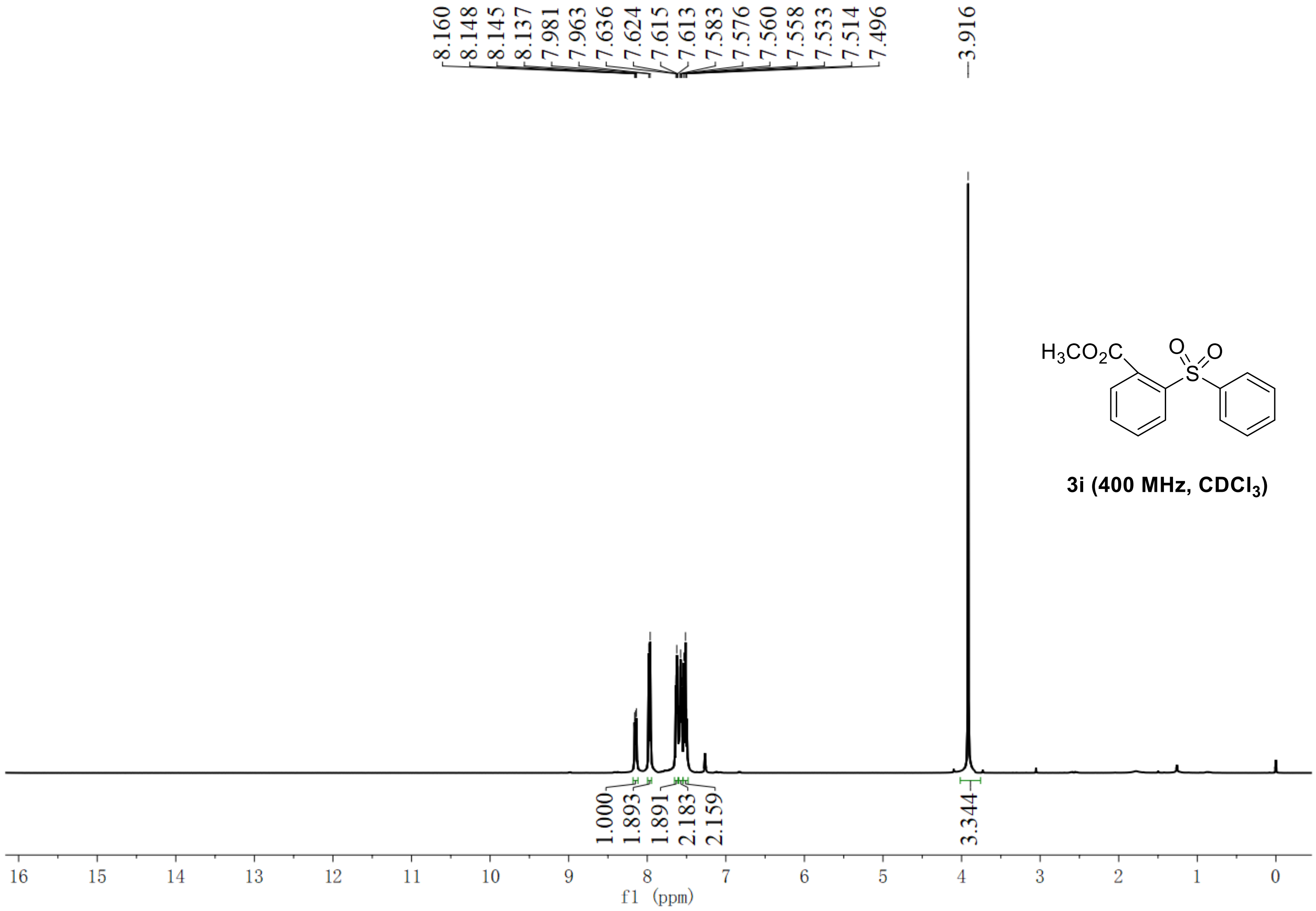

3i (400 MHz, $CDCl_3$)

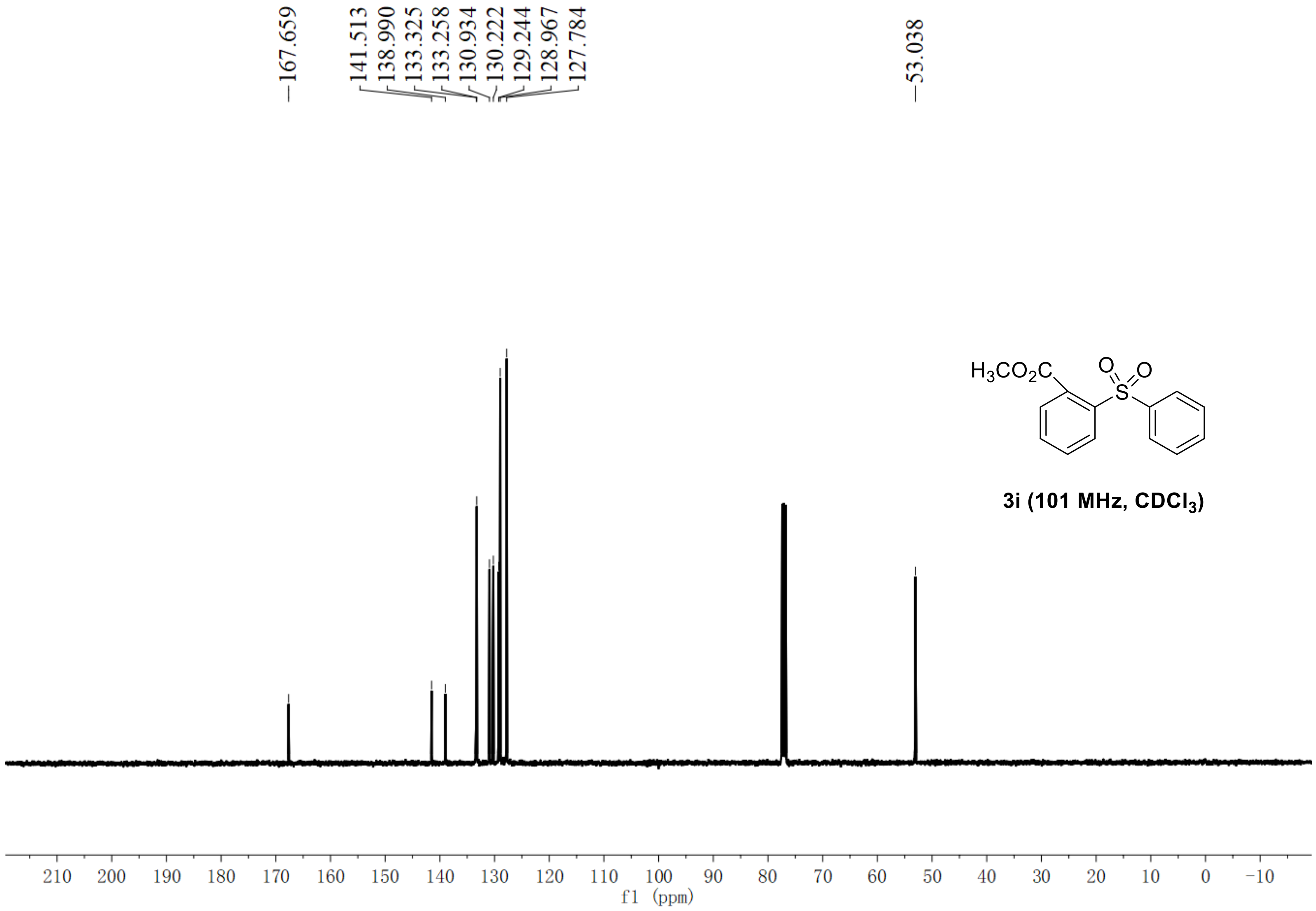

167.659
141.513
138.990
133.325
133.258
130.934
130.222
129.244
128.967
127.784
53.038
H3CO2C
O
S
O
3i (101 MHz, CDCl3)
210
200
190
180
170
160
150
140
130
120
110
100
90
80
70
60
50
40
30
20
10
0
−10
f1 (ppm)

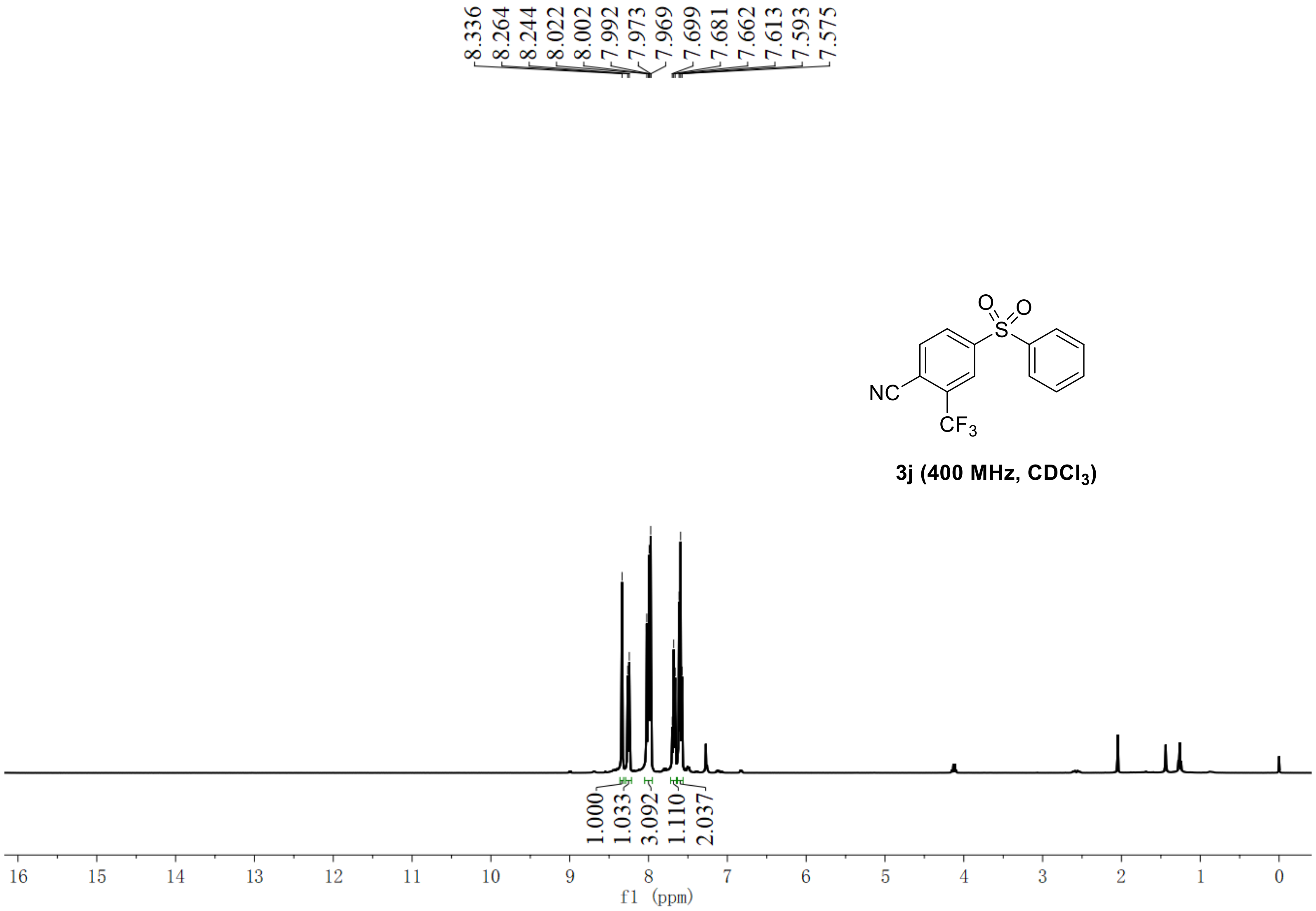

3j (400 MHz, $CDCl_3$)

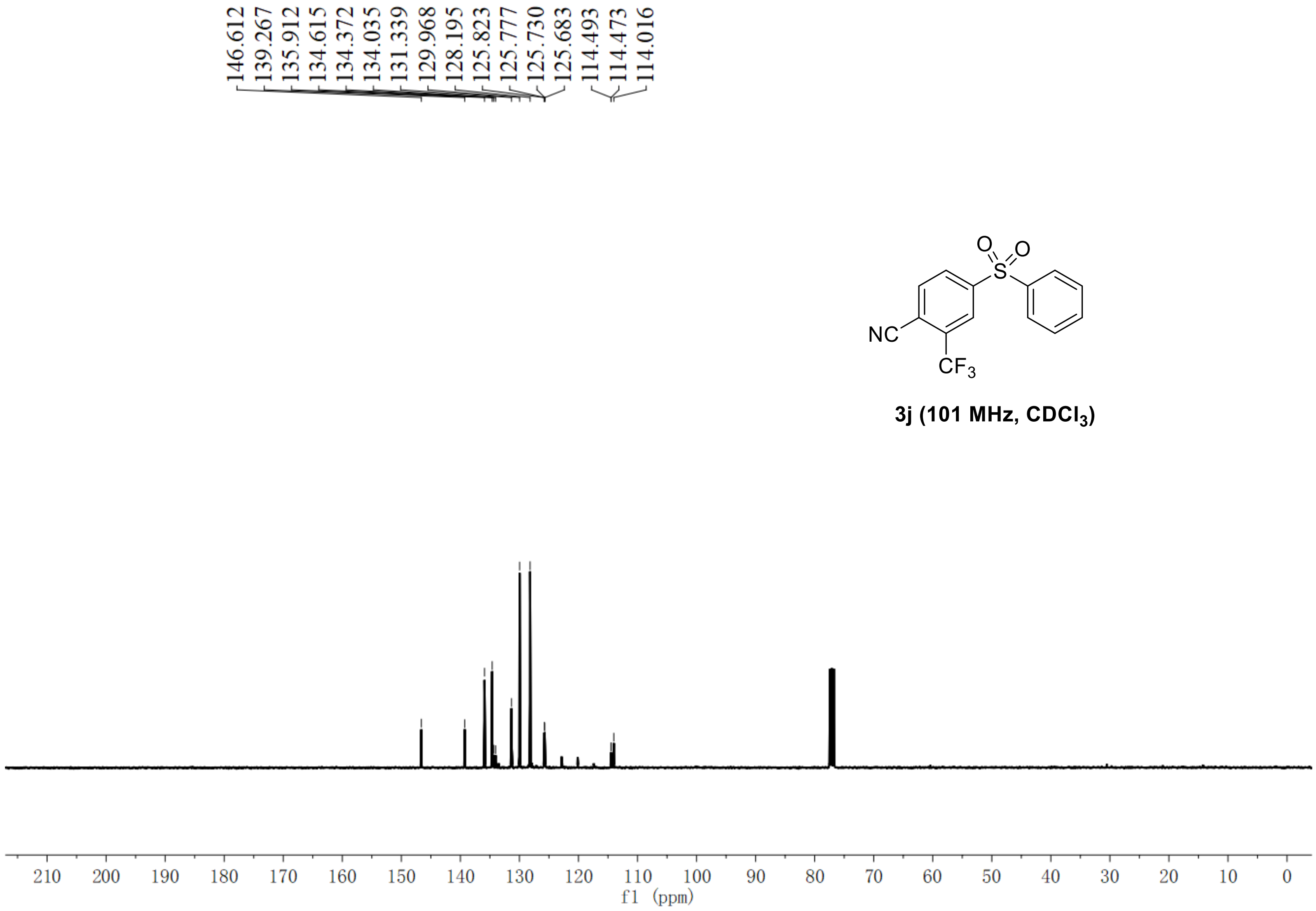

3j (101 MHz, $CDCl_3$)

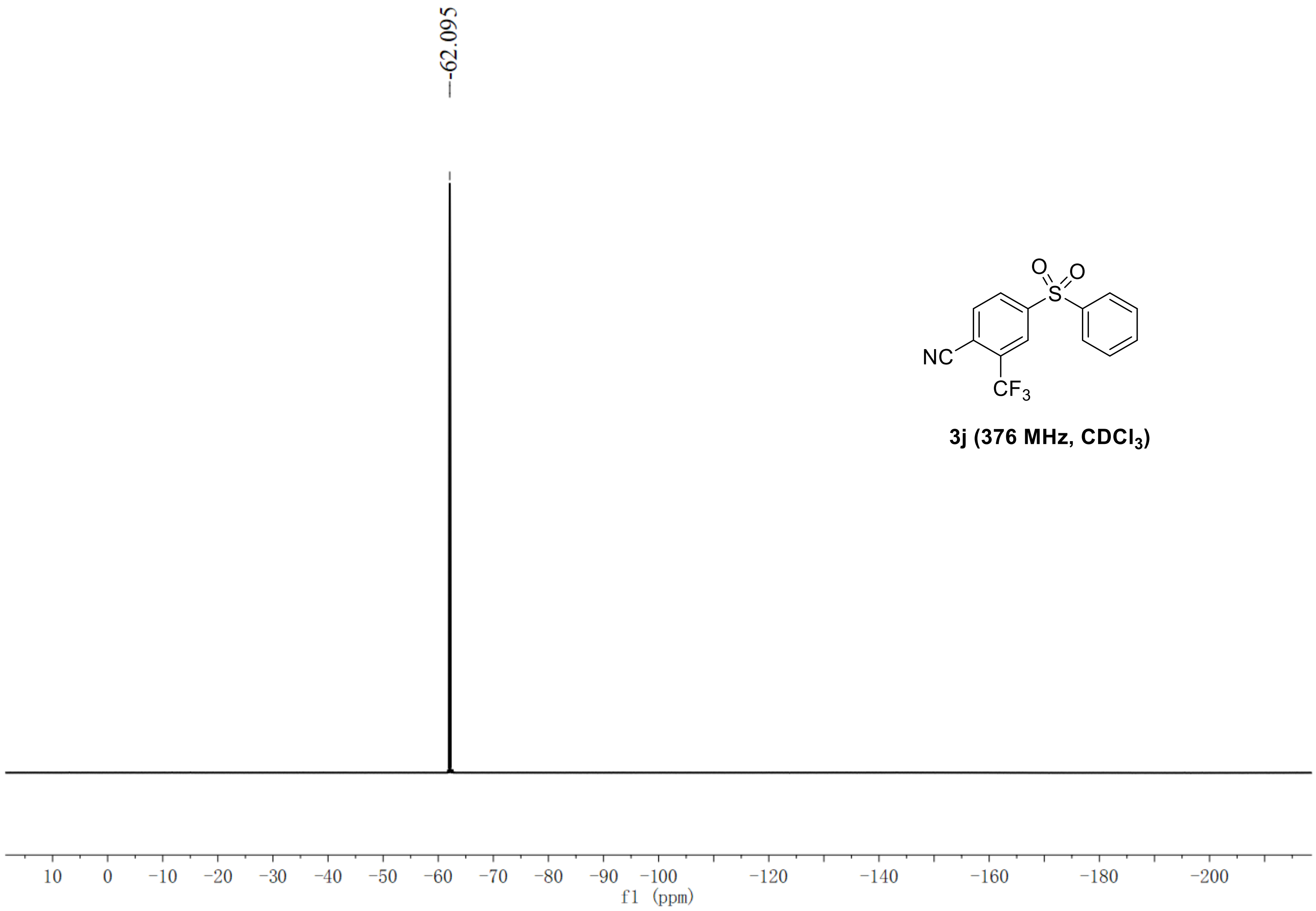

3j (376 MHz, $CDCl_3$)

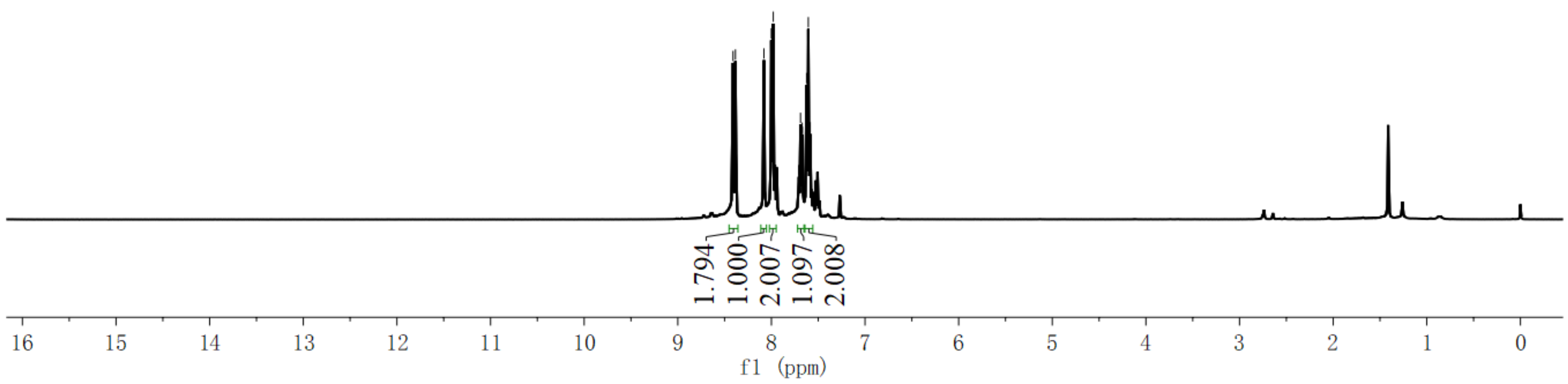

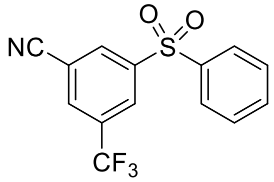

3k (400 MHz, $CDCl_3$)

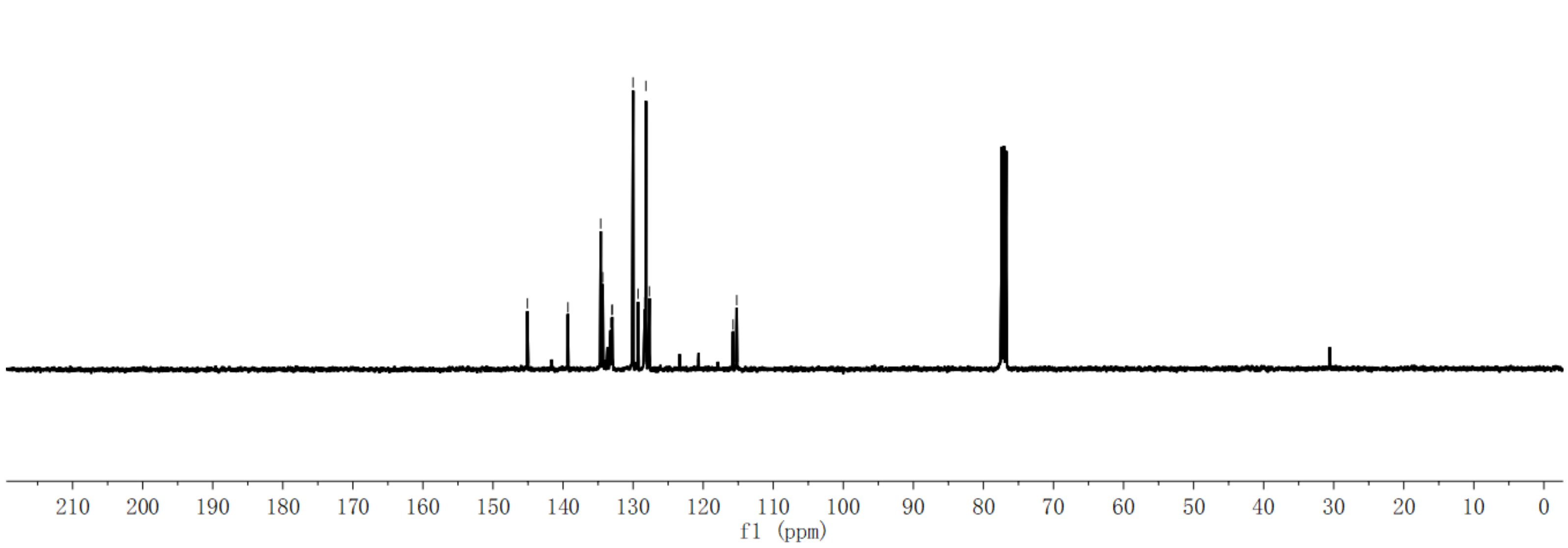

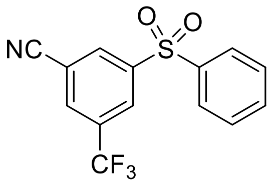

3k (101 MHz, $CDCl_3$)

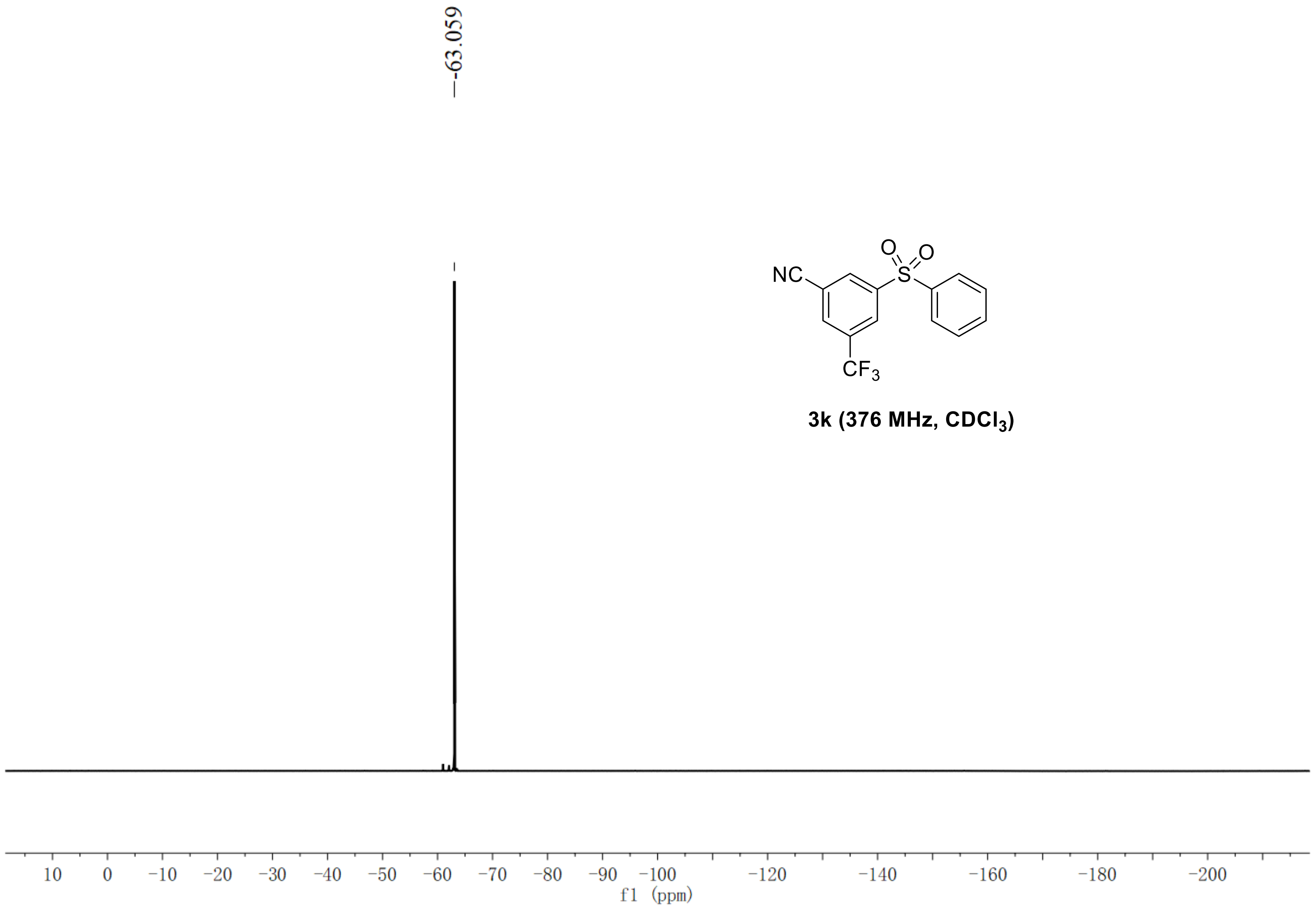
-63.059
NC
O
S
O
CF3
3k (376 MHz, CDCl3)
10
0
−10
−20
−30
−40
−50
−60
−70
−80
−90
−100
−120
−140
−160
−180
−200
f1 (ppm)

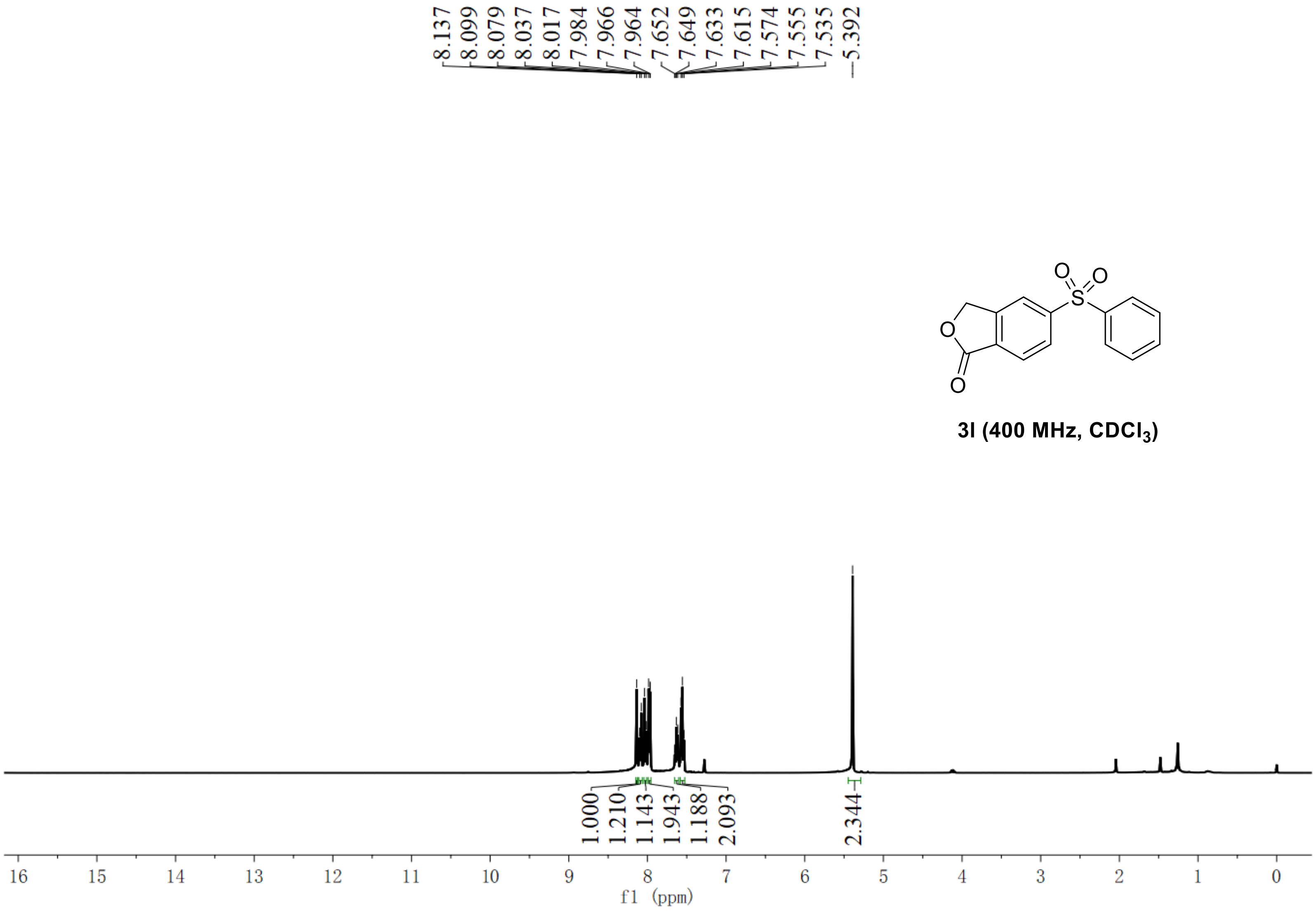

3l (400 MHz, $CDCl_3$)

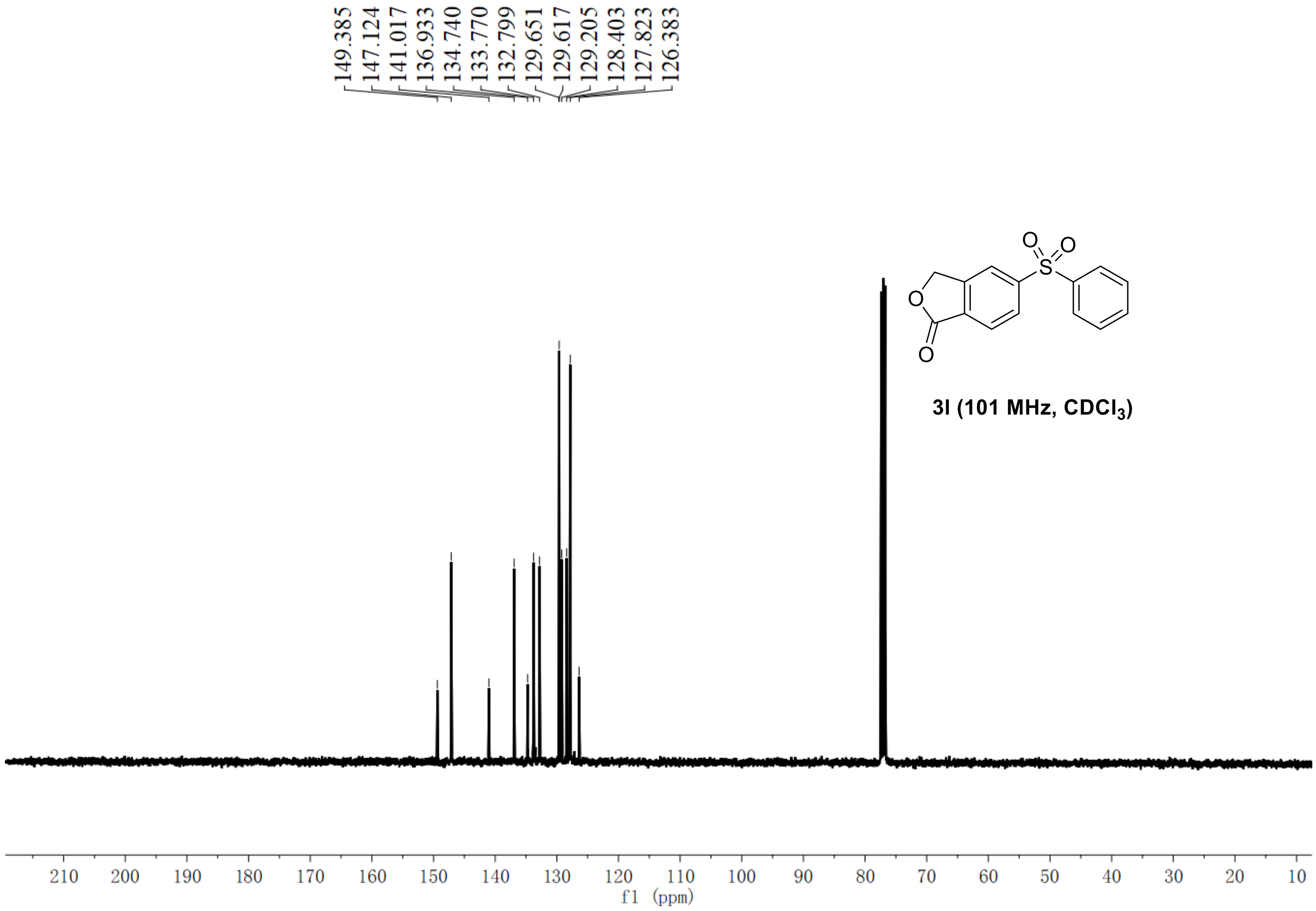

149.385
147.124
141.017
136.933
134.740
133.770
132.799
129.651
129.617
129.205
128.403
127.823
126.383
O
O
S
O
O
O
3l (101 MHz, CDCl3)
210
200
190
180
170
160
150
140
130
120
110
100
90
80
70
60
50
40
30
20
10
f1 (ppm)

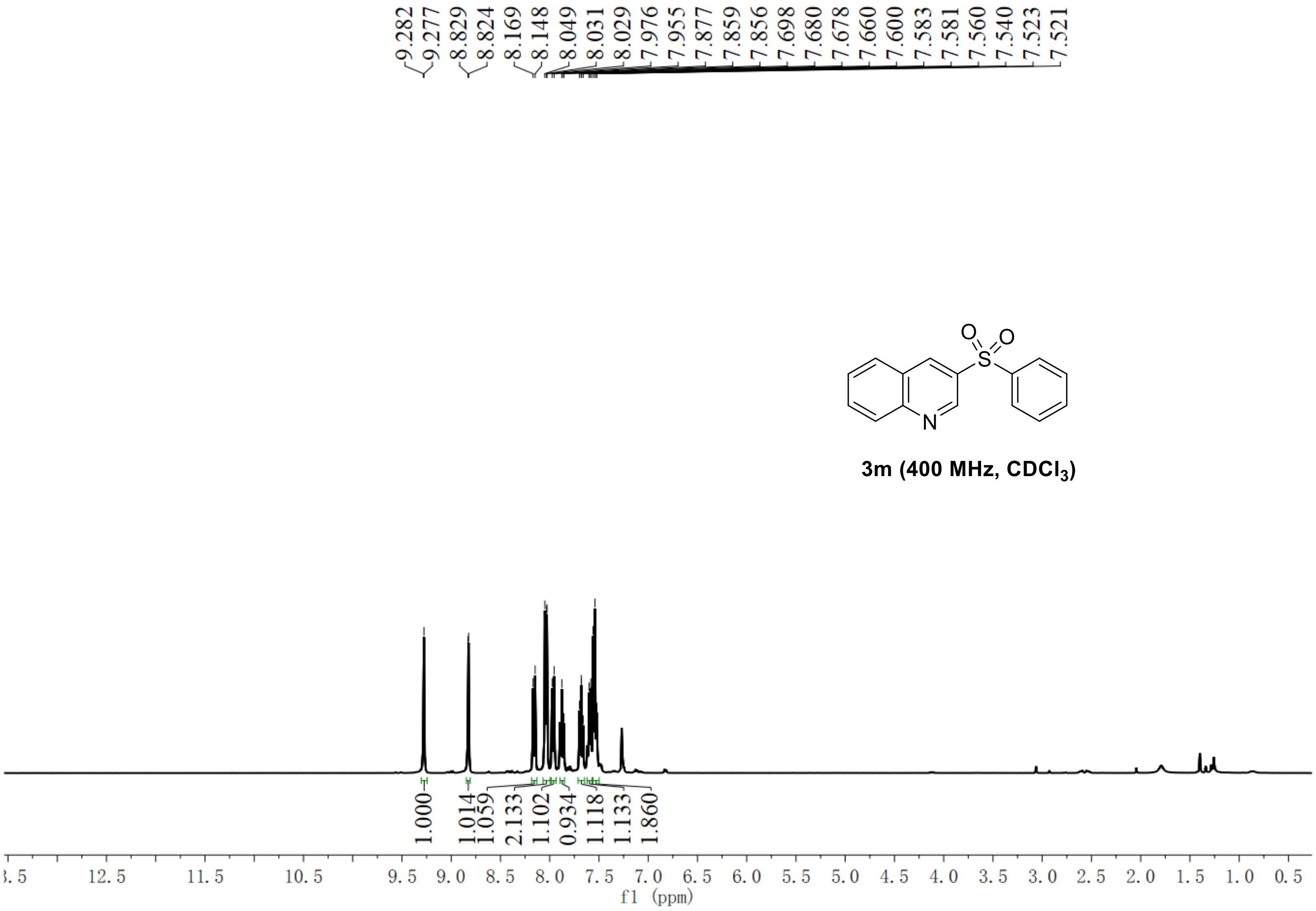

3m (400 MHz, $CDCl_3$)

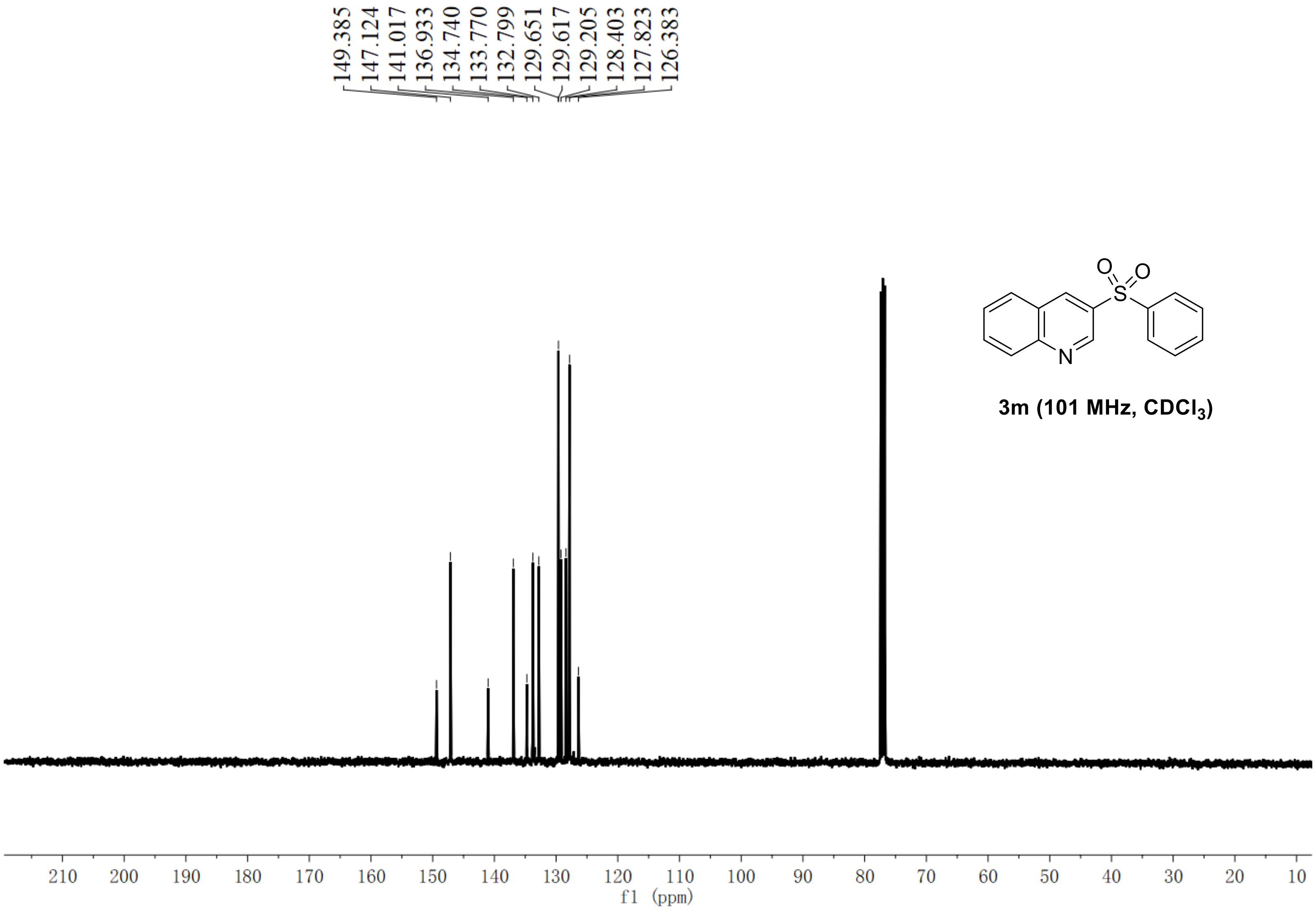
149.385
147.124
141.017
136.933
134.740
133.770
132.799
129.651
129.617
129.205
128.403
127.823
126.383
O
S
O
N
3m (101 MHz, $CDCl_3$)
210 200 190 180 170 160 150 140 130 120 110 100 90 80 70 60 50 40 30 20 10
f1 (ppm)

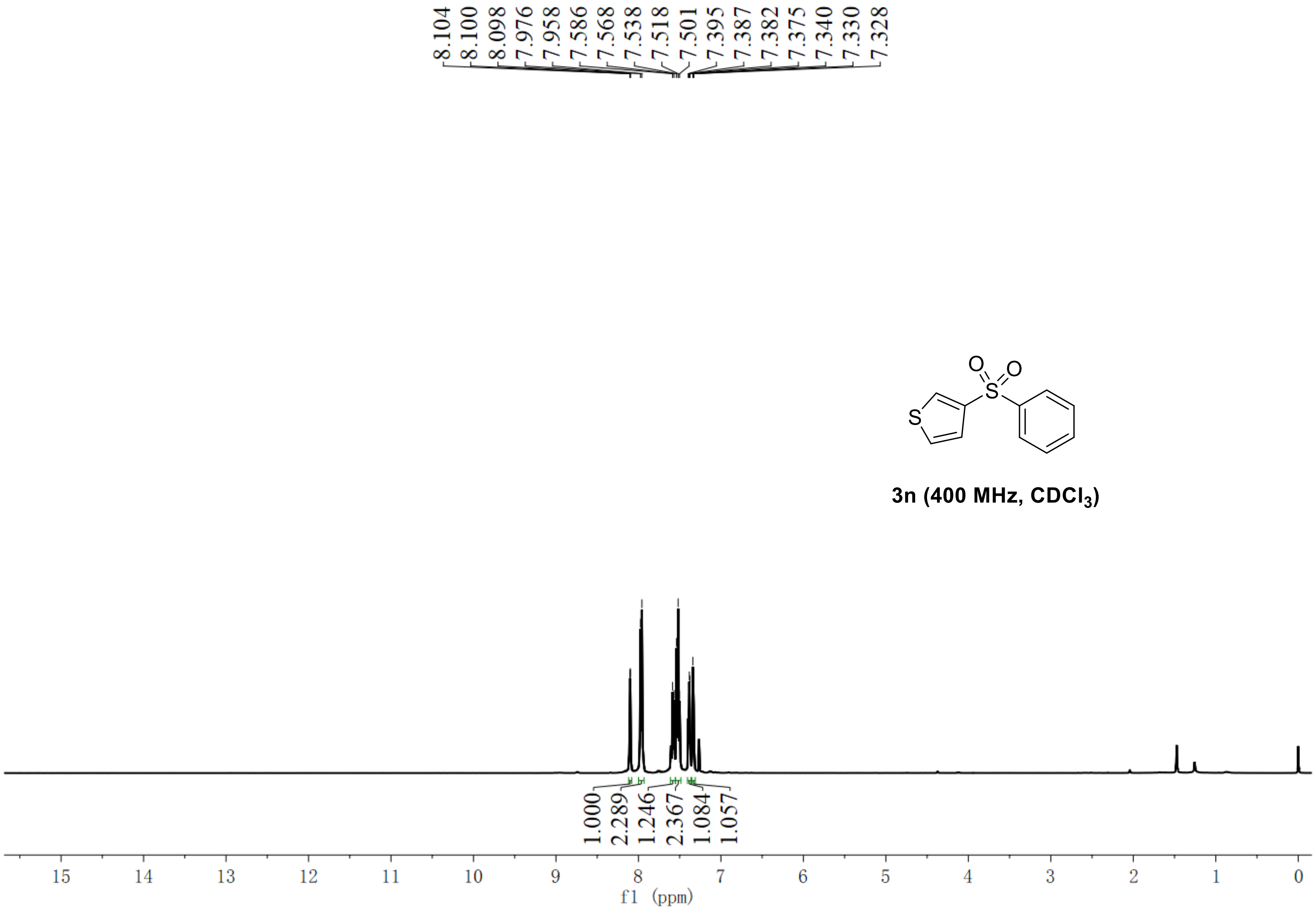
8.104
8.100
8.098
7.976
7.958
7.586
7.568
7.538
7.518
7.501
7.395
7.387
7.382
7.375
7.340
7.330
7.328
3n (400 MHz, CDCl3)
1.000
2.289
1.246
2.367
1.084
1.057
15
14
13
12
11
10
9
8
7
6
5
4
3
2
1
0
f1 (ppm)

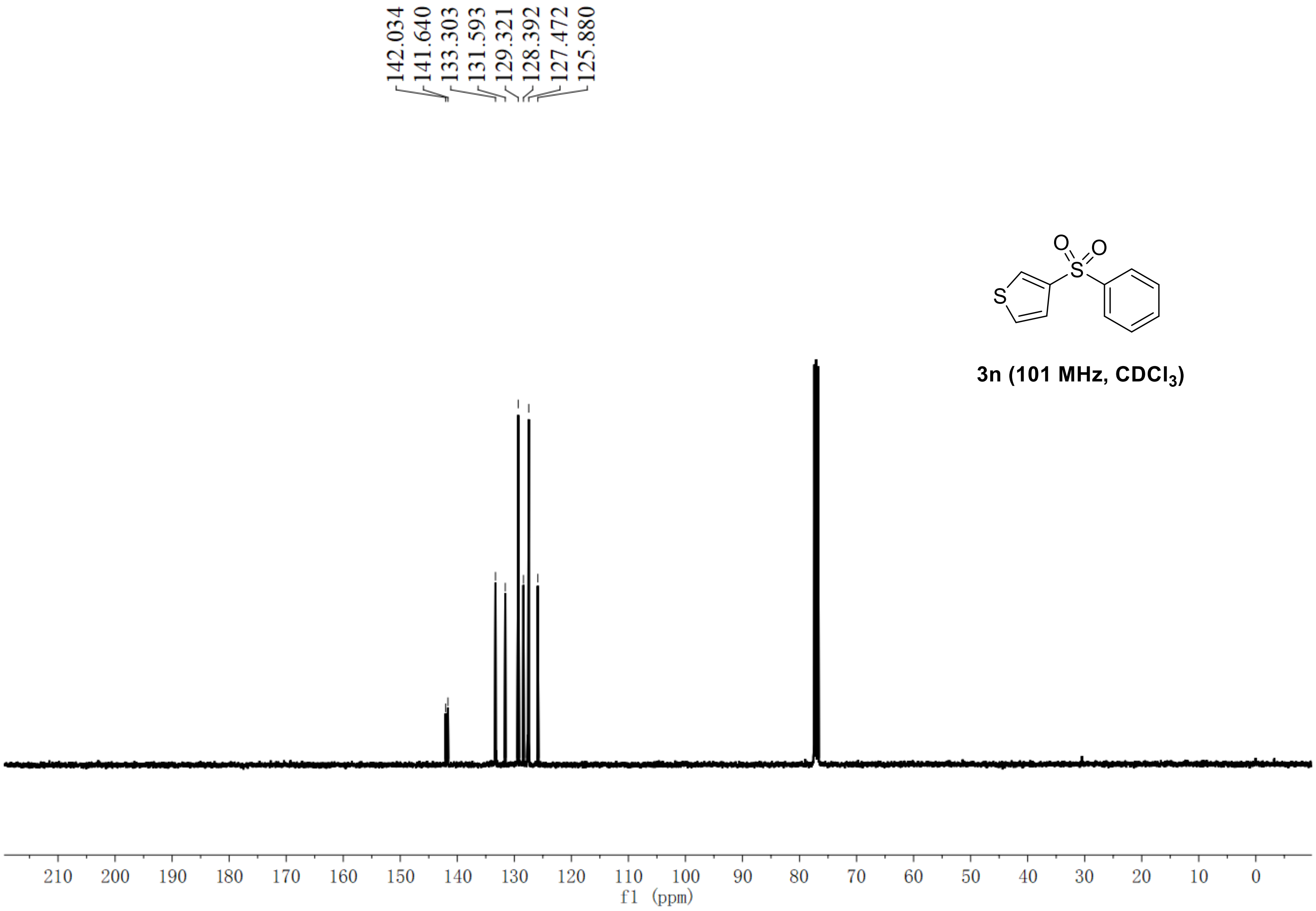

3n (101 MHz, $CDCl_3$)

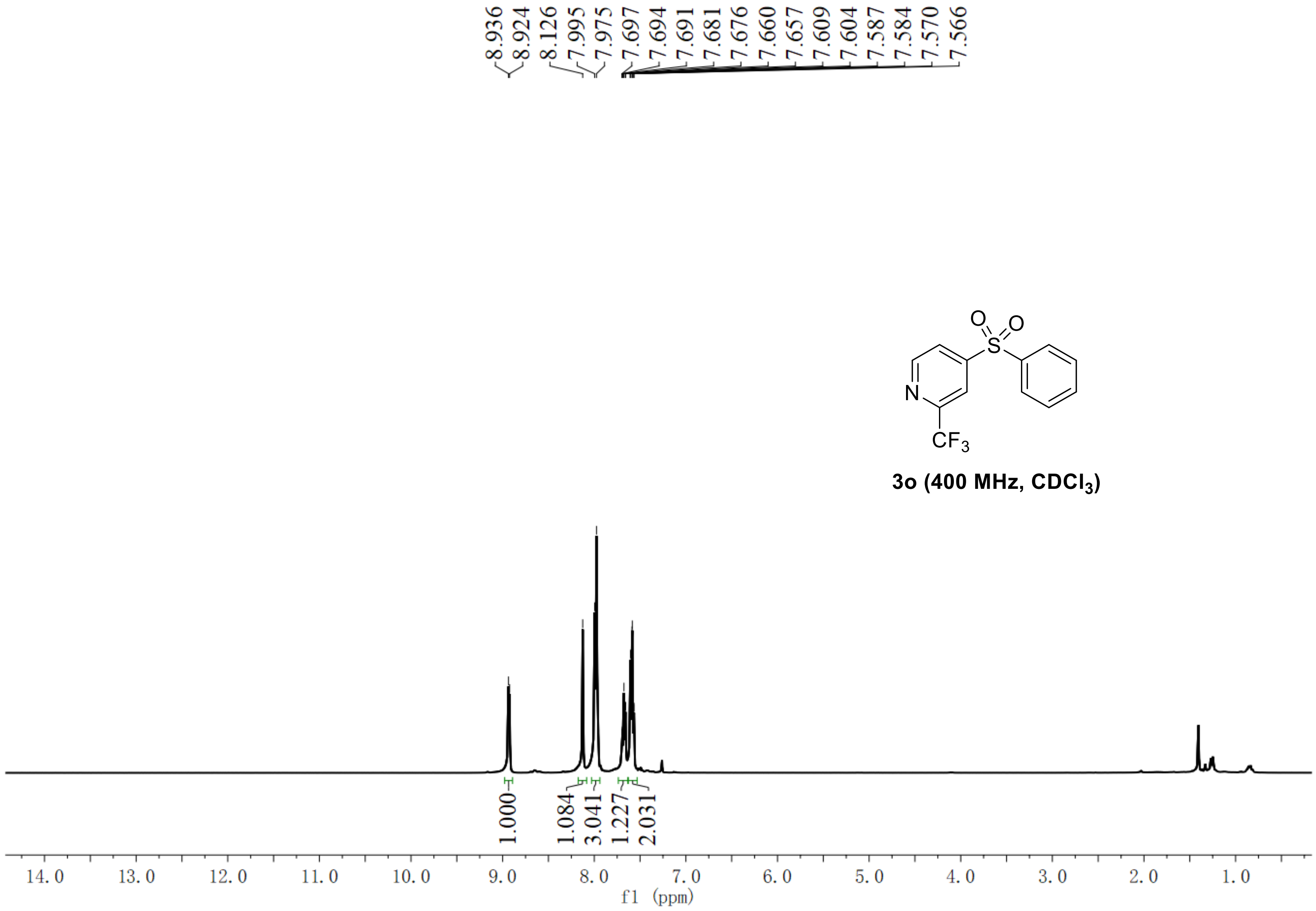

3o (400 MHz, $CDCl_3$)

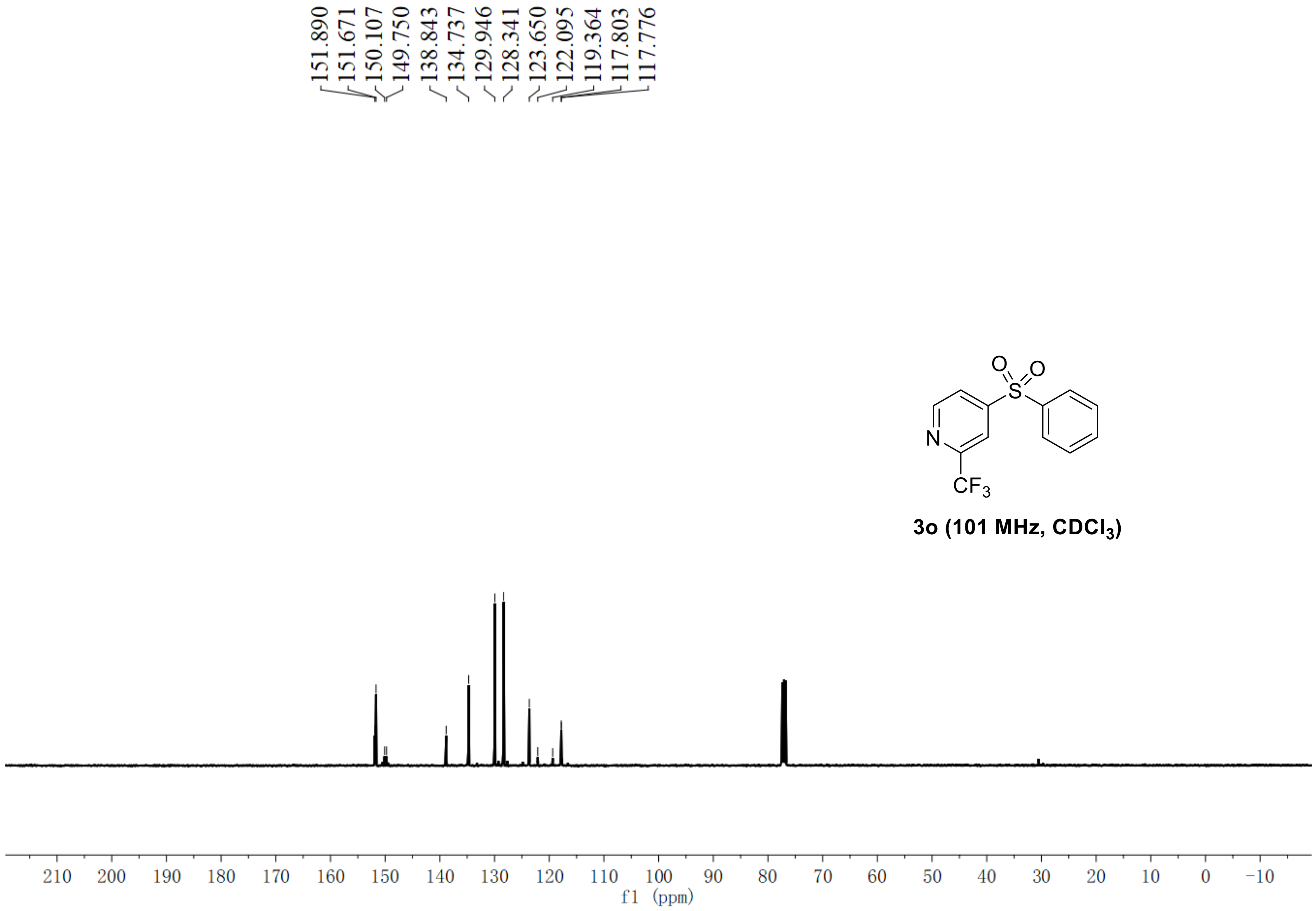

3o (101 MHz, $CDCl_3$)

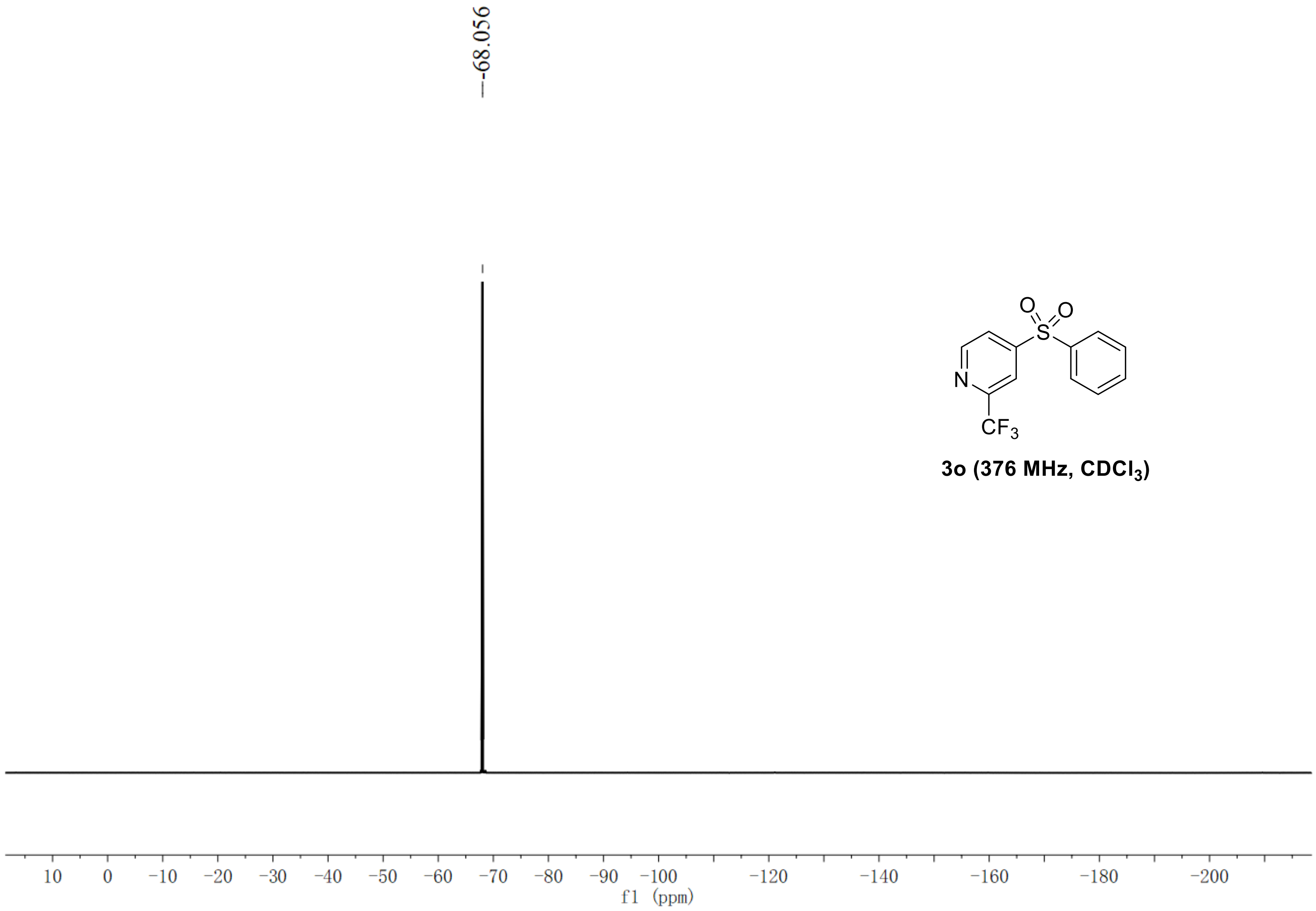

3o (376 MHz, $CDCl_3$)

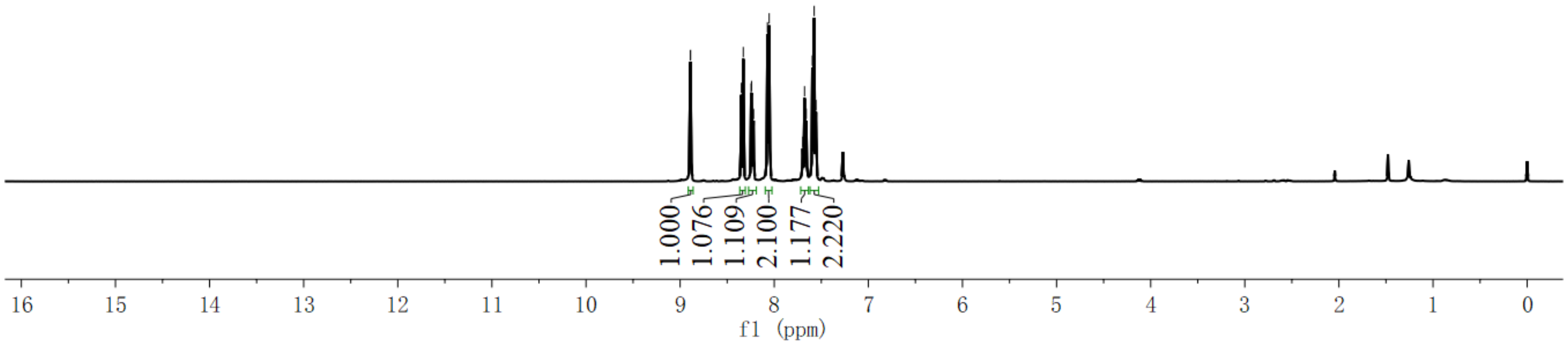

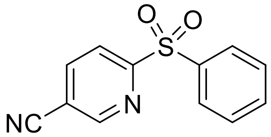

3p (400 MHz, $CDCl_3$)

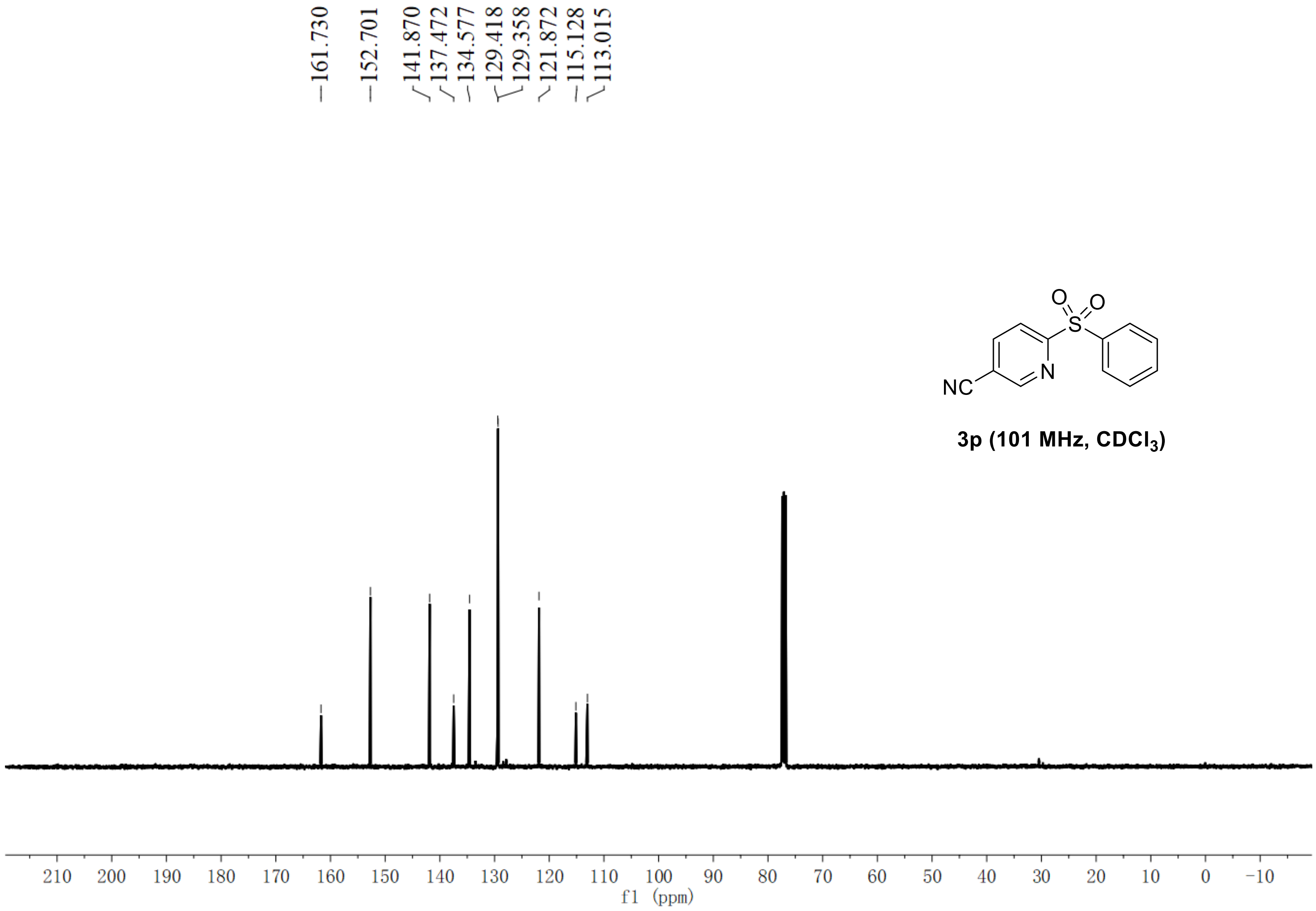

3p (101 MHz, $CDCl_3$)

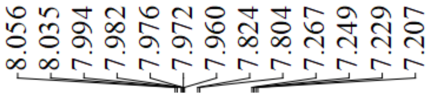

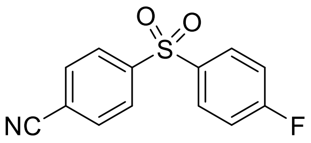

3q (400 MHz, $CDCl_3$)

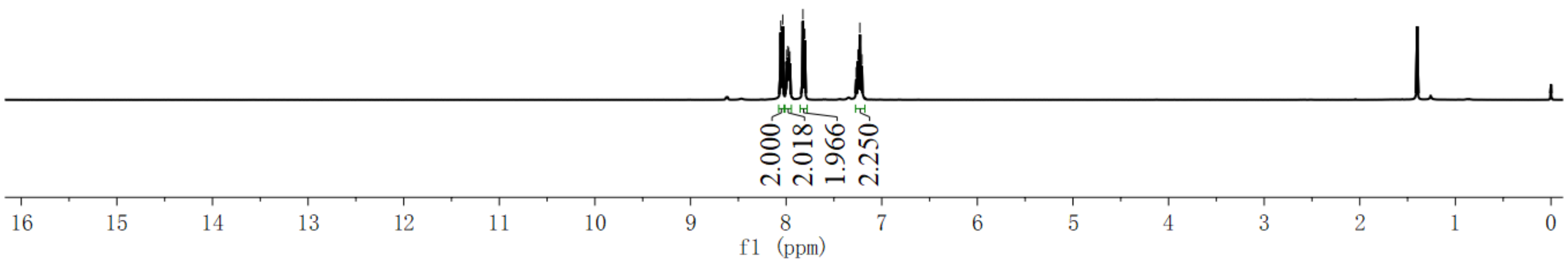

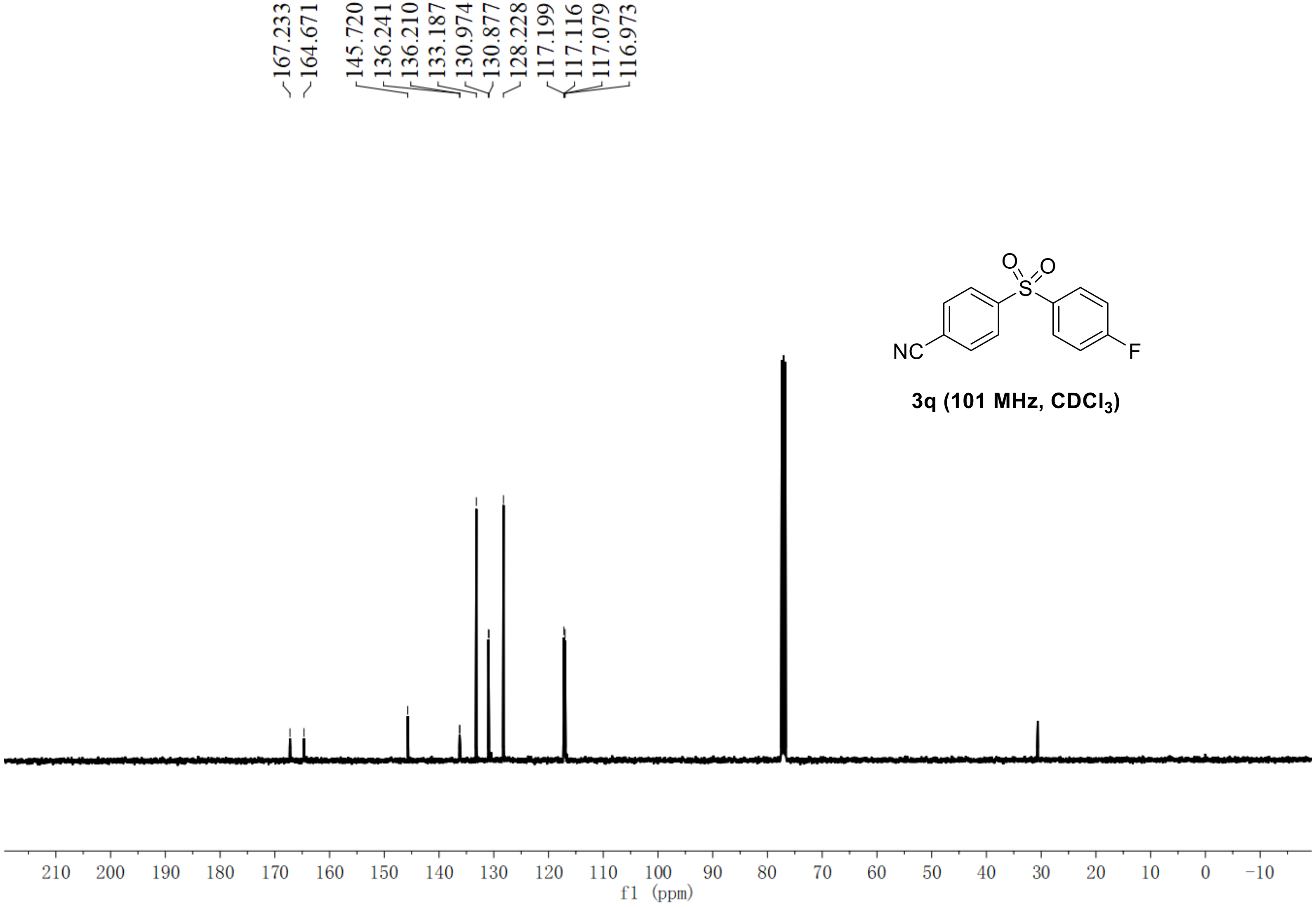

3q (101 MHz, $CDCl_3$)

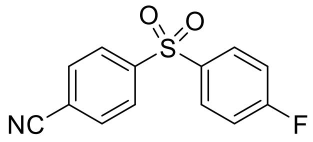

3q (376 MHz, CDCl$_3$)

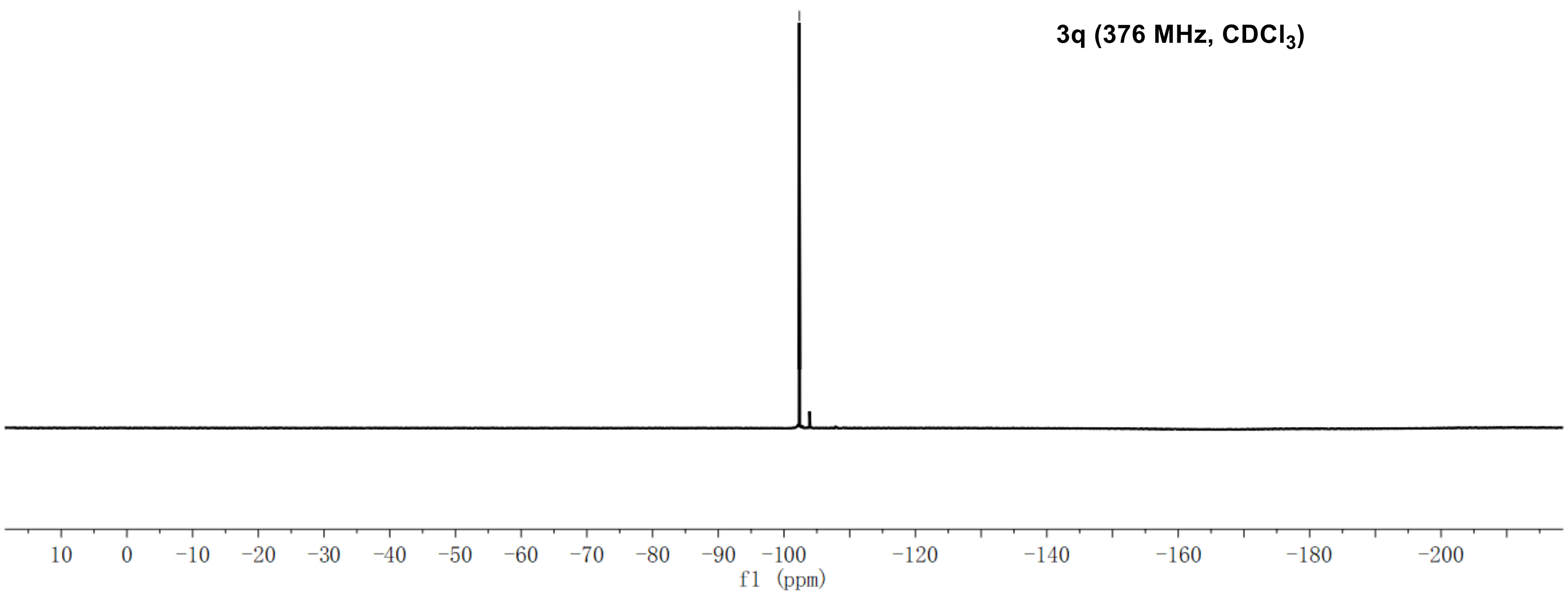

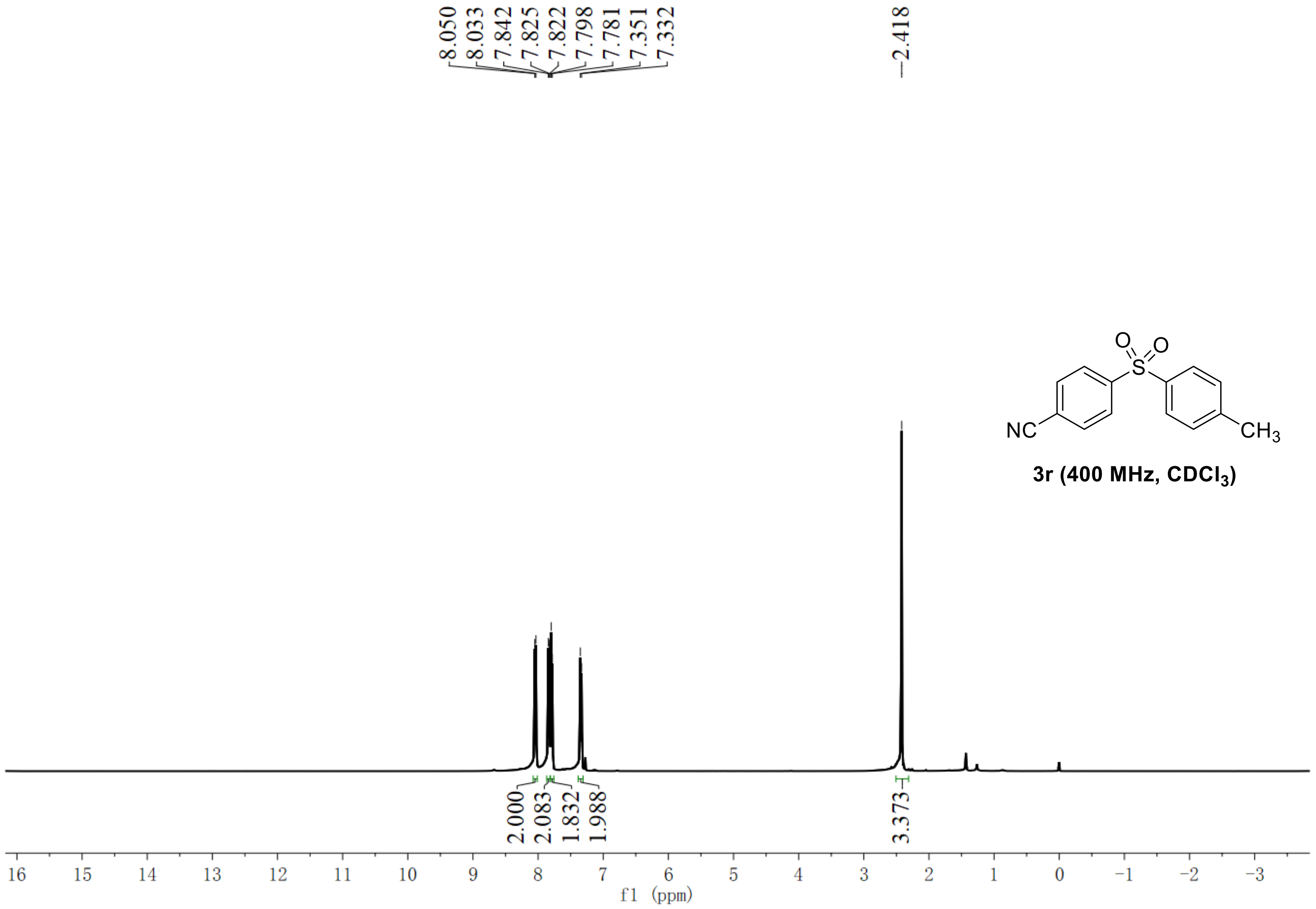
8.050
8.033
7.842
7.825
7.822
7.798
7.781
7.351
7.332
2.418
NC
CH3
3r (400 MHz, CDCl3)
2.000
2.083
1.832
1.988
3.373
16 15 14 13 12 11 10 9 8 7 6 5 4 3 2 1 0 −1 −2 −3
f1 (ppm)

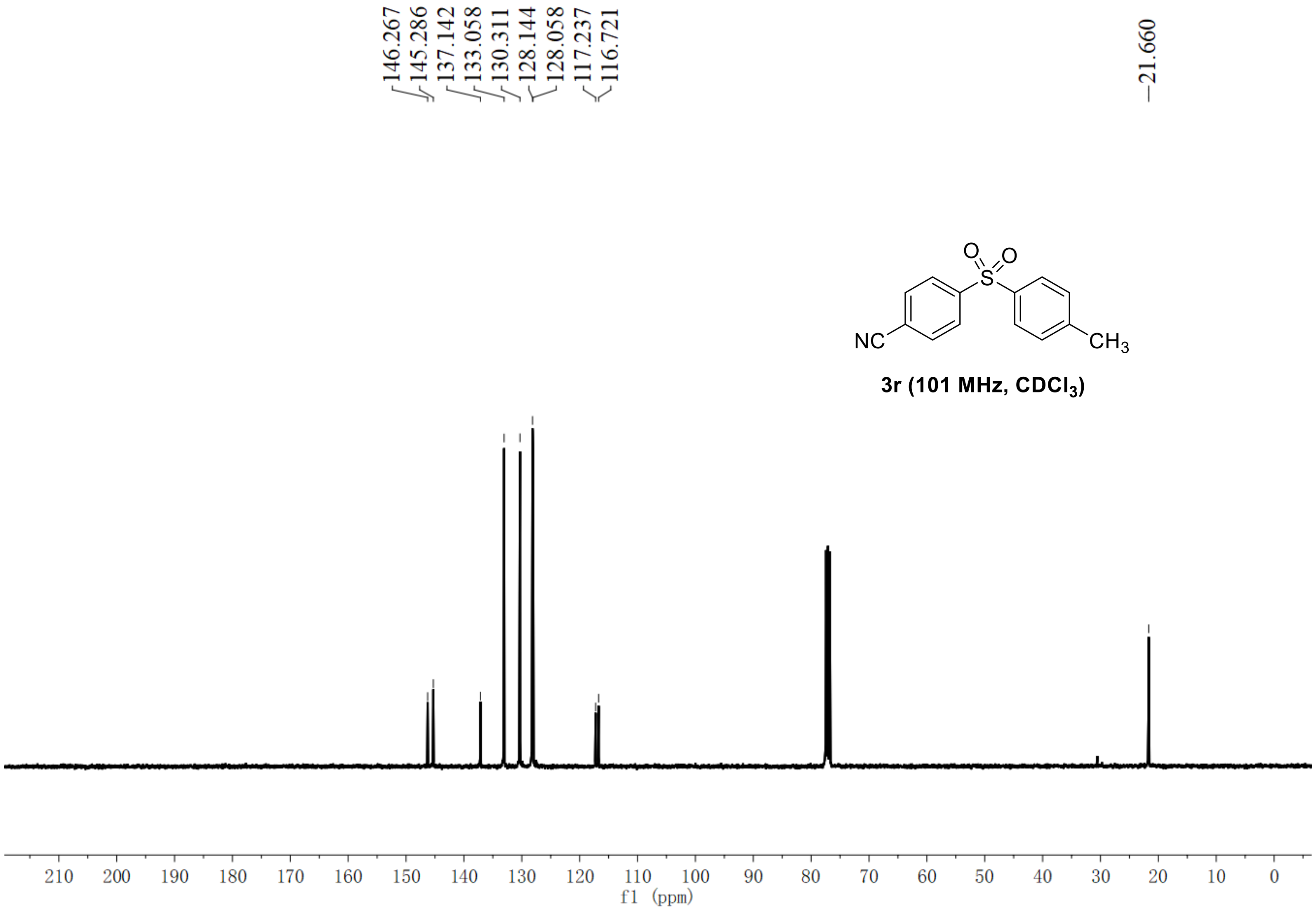

3r (101 MHz, $CDCl_3$)
